# Master and servant: LINC00152 – a STAT3-induced long noncoding RNA regulates STAT3 in a positive feedback in human multiple myeloma

**DOI:** 10.1186/s12920-020-0692-3

**Published:** 2020-02-10

**Authors:** Stefanie Binder, Ivonne Zipfel, Maik Friedrich, Diana Riedel, Stefanie Ende, Christoph Kämpf, Karolin Wiedemann, Tilo Buschmann, Sven-Holger Puppel, Kristin Reiche, Peter F. Stadler, Friedemann Horn

**Affiliations:** 1 Institute of Clinical Immunology, Faculty of Medicine, University of Leipzig, Leipzig, Germany; 2 Fraunhofer Institute for Cell Therapy and Immunology, Department of Diagnostics, Leipzig, Germany; 3 Bioinformatics Group, Department of Computer Science, and Interdisciplinary Center for Bioinformatics, University of Leipzig, Leipzig, Germany; 4 German Centre for Integrative Biodiversity Research – iDiv, Halle-Jena-Leipzig, Germany; 5 Max Planck Institute for Mathematics in the Sciences, Leipzig, Germany; 6 Department of Theoretical Chemistry, University of Vienna, Wien, Austria; 7 Center for RNA in Technology and Health, University of Copenhagen, København, Denmark; 8 Santa Fe Institute, Santa Fe, USA

**Keywords:** STAT3, LINC00152, CYTOR, ncRNAs, IL-6 signaling, Epigenetics, Cancer

## Abstract

**Background:**

The survival of INA-6 human multiple myeloma cells is strictly dependent upon the Interleukin-6-activated transcription factor STAT3. Although transcriptional analyses have revealed many genes regulated by STAT3, to date no protein-coding STAT3 target gene is known to mediate survival in INA-6 cells. Therefore, the aim here was to identify and analyze non-protein-coding STAT3 target genes. In addition to the oncogenic microRNA-21, we previously described five long noncoding RNAs (lncRNAs) induced by STAT3, named STAiRs. Here, we focus on STAT3-induced RNA 18 (STAiR18), an mRNA-like, long ncRNA that is duplicated in the human lineage. One STAiR18 locus is annotated as the already well described LINC00152/CYTOR, however, the other harbors the MIR4435-2HG gene and is, up to now, barely described.

**Methods:**

CAPTURE-RNA-sequencing was used to analyze STAiR18 transcript architecture. To identify the STAiR18 and STAT3 phenotype, siRNA-based knockdowns were performed and microarrays were applied to identify their target genes. RNA-binding partners of STAiR18 were determined by Chromatin-Isolation-by-RNA-Purification (ChIRP) and subsequent sequencing. STAT3 expression in dependence of STAiR18 was investigated by immunoblots, chromatin- and RNA-immunoprecipitations.

**Results:**

As identified by CAPTURE-RNA sequencing, a complex splice pattern originates from both STAiR18 loci, generating different transcripts. Knockdown of the most abundant STAiR18 isoforms dramatically decreased INA-6 cell vitality, suggesting a functional role in myeloma cells. Additionally, STAiR18 and STAT3 knockdowns yielded overlapping changes of transcription patterns in INA-6 cells, suggesting a close functional interplay between the two factors. Moreover, Chromatin isolation by RNA purification (ChIRP), followed by genome-wide RNA sequencing showed that STAiR18 associates specifically with the STAT3 primary transcript. Furthermore, the knockdown of STAiR18 reduced STAT3 levels on both the RNA and protein levels, suggesting a positive feedback between both molecules. Furthermore, STAiR18 knockdown changes the histone methylation status of the STAT3 locus, which explains the positive feedback and indicates that STAiR18 is an epigenetic modulator.

**Conclusion:**

Hence, STAiR18 is an important regulator of myeloma cell survival and is strongly associated with the oncogenic function of STAT3. The close functional interplay between STAT3 and STAiR18 suggests a novel principle of regulatory interactions between long ncRNAs and signaling pathways.

## Background

Interleukin-6 (IL-6) is a hallmark of a wide range of biological functions, including immune regulation, hematopoiesis, inflammation and tumor development [1]. IL-6 operates as a pro-inflammatory and anti-apoptotic stimulus through an intracellular signaling cascade [2]. Binding of IL-6 to its plasma membrane receptor activates receptor-associated Janus kinases (JAKs), which in turn phosphorylate intracellular targets [3] including Signal Transducer and Activator of Transcription 3 (STAT3). Upon STAT3 phosphorylation it is shuttled to the nucleus, where it activates the transcription of target genes [4]. In multiple myeloma, IL-6-activated STAT3 plays a major oncogenic role through the regulation of cell survival and proliferation. As reported earlier, the IL-6-dependent human myeloma cell line INA-6 responds with a remarkably rapid and complete apoptosis to cytokine withdrawal [5]. Previously, we identified microRNA-21 as a STAT3 target with anti-apoptotic function in INA-6 cells [6], however, it is not the sole survival mediator in these cells. Furthermore, in a genome-wide transcription study in INA-6 cells we demonstrated that in addition to protein-coding mRNAs, IL-6 regulates the transcription of a large number of long noncoding RNAs (lncRNAs) [7]. In a previous publication [8], we characterized five of these IL-6-induced lncRNAs (STAiR1, STAiR2, STAiR6, STAiR15 and STAiR18) in more detail, verifying them as STAT3 targets, and therefore named them STAT3-induced ncRNAs (STAiRs). Out of this pool of STAiRs, we focused on STAiR18 for the following reasons: In contrast to the unprocessed macroRNAs STAiR1, − 2 and − 6, STAiR15 and − 18 were spliced and therefore suited better to carry out functional analyses by siRNA-based knockdown strategies. Moreover, at the time we identified the STAiRs, STAiR15 (alias MIAT) was already described in great detail [9, 10] in contrast to STAiR18. Most importantly, only STAiR18 showed a global overexpression in various cancer types [8]. This observation has since been confirmed by further publications [11–13]. Therefore, our preliminary data supported the view that STAiR18 contributes to STAT3-dependent tumorigenesis in multiple myeloma as well as in other cancer entities [8]. This observation has since been confirmed by further publications [11–13]. Therefore, our preliminary data supported the view that STAiR18 contributes to STAT3-dependent tumorigenesis in multiple myeloma as well as in other cancer entities [8]. In this study, we aimed to characterize the molecular function of STAiR18 in INA-6 cells in order to clarify whether it has an impact on myeloma cell survival. The goal was to elucidate the role of STAiR18 within the STAT3 signaling cascade.

## Methods

### Cell culture

INA-6 cells were kindly provided by the Gramatzki group (Kiel, Germany), who removed these cells originally from an 80 year old multiple myeloma patient and brought them into cell culture. Cells were maintained in RPMI1640 + GlutaMAX™ (LIFE Technologies, Carlsbad, California, USA), supplemented with 10% fetal calf serum (Lonza, Basel, Switzerland) and 1% penicillin/streptomycin (LIFE Technologies), and 1 ng IL-6 per ml medium. Where indicated, INA-6 cells were withdrawn from IL-6 for at least 12 h with an optional IL-6 restimulation.

### General experimental design

Each experiment was performed in a minimum of three independent biological replicates (*n* ≥ 3). Data are shown as means, and error bars represent standard deviation (SD). A two-sided and unpaired t-test was used to assess statistical significance.

### Apoptosis and cell vitality assays

Apoptosis rates were determined by either the Dead Cell Apoptosis Kit (LIFE Technologies) or the Caspase-Glo® 3/7 Assay (Promega, Fitchburg, Wisconsin, USA) according to the manufacturerˈs protocols. Cell vitality was examined by CellTiter-Glo® Luminescent Cell Viability Assay (Promega). Analysis was carried out by either FACSCalibur™ (BD Biosciences, Franklin Lakes, New Jersey, USA) together with the corresponding software CellQuest™ or LUMIstar Optima (BMG Labtech, Ortenberg, Germany) together with the corresponding software Optima, respectively.

### Isolation and analysis of RNA and DNA

RNA and DNA were isolated with TRIzol (LIFE Technologies) and Phenol-Chloroform-Isoamylalcohol (Sigma-Aldrich, St. Louis, Missouri, USA), respectively, following the manufacturerˈs protocols. RNA was DNase-digested using TURBO-DNA-free Kit (LIFE Technologies). Reverse transcription of RNA was conducted using the RevertAid First Strand cDNA synthesis kit (Thermo, Waltham, Massachusetts, USA). Analyses of DNA and cDNA were performed using Light Cycler® Fast Start DNA Master Plus SYBR Green kit (Roche) as described by the manufacturer using Light Cycler®. Primers are listed in the Additional file 1: Table S1.

### Analysis of transcript half life

INA-6 cells were treated with 5 μg/ml Actinomycin D (in DMSO) for up to 6 h. Cells were harvested after indicated time points and transcript half-life was assessed by qPCR in comparison to DMSO control.

### Analysis of transcript copy number

To determine the copy number of STAiR18 and cyclophillin B a sequential dilution of pcDNA 3.1 (+) harboring either STAiR18 or cypB inserts (see Additional file 1: Figure S1 and S2) was performed to generate a type curve by qPCR. The copy number of plasmids was calculated by the Avogadro constant (6.022 × 10^23^ mol^− 1^), the molar mass of a single base pair (650 g/mol), the plasmid size (5872 bp for pcDNA-STAiR18 and 6079 for pcDNA-cypB), its concentration (both 1 μg/μl) and volume (5 μl) used for qPCR by the following equation:

$$ \kern2.25em number\ of\ copies=\frac{\left( mass\ (g)\ x\  Avogadro\ constat\ \Big( mo{l}^{-1}\right)\Big)}{\left( plasmid\ size\ (bp)x\ 650\ \left(\frac{g}{mol}\right)\right)\ } $$

We used 3 × 10^3^ to 3 × 10^8^ copies of both plasmids to generate a type curve. Additionally, we isolated RNA from 1 × 10^6^ INA-6 cells and determined the copy number of STAiR18 and cypB transcripts by RT-qPCR using the same primer pairs as for the corresponding plasmid by means of this type curve.

### STAiR18- and STAT3-knockdown

Cells were transfected with 200 pmol stealth-siRNAs (listed in the Additional file 1: Table S2) per 5 × 10^6^ cells. Transfection was carried out using the NEON™-Kit and the microporator MP100 Digitalbio (LIFE Technologies) according to the manufacturer’s instructions. Three pulses of 1600 V and 10 ms were applied.

### Immunoblotting

Proteins were isolated using cell lysis buffer (50 mM Tris/HCl pH 7.2, 150 mM NaCl, 5 mM NaF, 0.25 mM EDTA, 1% Triton-X-100, 1% SDS, 1 mM NaVO_4_, 5 μg/ml pepstatin, 5 μg/ml leupeptin, 0.14 U/ml aprotinin). After a Bradford analysis, 30 μg of lysates were supplemented with Laemmli buffer, boiled, separated in a 10–15% SDS gel and blotted to a polyvinylidene fluoride membrane. Protein bands were visualized using primary and secondary antibodies (Additional file 1: Table S3), the Super Signal® West Dura detection reagent (Thermo), and a CCD camera (Raytest, Straubenhardt, Germany). Each immunoblot shown is a representative example out of a minimum of three independent biological replicates (*n* > 3). Where indicated, a quantitative analysis of band signals was performed using the software XStella.

### Chromatin- and RNA Immunoprecipitation

Chromatin-Immunoprecipitation (ChIP) was performed using the EZ ChIP kit (Upstate, Lake Placid, New York, USA) according to the manufacturerˈs instructions together with antibodies targeting H3K4me3, H3K27me3 (Cell Signaling, Cambridge, UK; #9727S and #9733S, respectively), H3K36me3 (Abcam, Cambridge, UK; ab9050), and an IgG negative control (Abcam; ab37415). For each IP approach, 5 μg antibody was used for 5 × 10^6^ cells. Each IP was performed in a minimum of three independent biological replicates (n > 3). Data are shown as means and error bars are given as standard deviations (SD).

### Genome-wide transcriptional analysis

Gene expression of an experiment performed in four independent biological replicates (*n* = 4) was analyzed using SurePrint G3 Human Gene Expression v2 8x60K Microarrays (Agilent Technologies, Santa Clara, USA, California) and the belonging OneColor Quick Amp Labeling kit according to the manufacturer’s instructions. Quality controlled libraries were hybridized to the array and signal detection occurred by microarray scanner (Agilent Technologies). Raw data files were processed by the GeneSpring 13.0 software (Agilent Technologies). A paired t-test without a multiple testing correction was executed for statistical analysis. Data were deposited in the GEO database [GEO:GSE71092].

### CAPTURE- and ChIRP-seq experiments

CAPTURE and ChIRP experiments with or without a subsequent Next Generation Sequencing were performed as described in Binders et al. 2017. The oligonucleotides used for RNA pulldowns are listed in Additional file 1: Table S4. The CAPTURE-seqs of STAiR18 and lacZ are stored in the GEO database [GEO:GSM2496675] and [GEO:GSM2496676]. ChIRP-seqs of STAiR18 and lacZ are stored in [GEO:GSM2496682] and [GEO:GSM2496683].

## Results

### STAiR18 is transcribed from a duplicated genomic locus

**Fig. 1:**
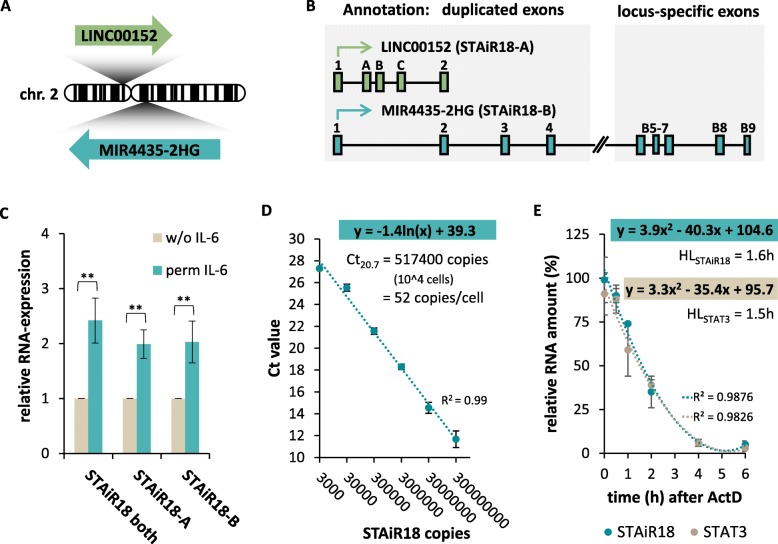
STAiR18 is duplicated in the human genome and expressed like an mRNA. **a** Schematic representation of duplicated STAiR18 on chromosome 2 with the LINC00152 locus on the plus and the MIR4435-2HG locus on the minus strand. **b** Transcripts derived from the LINC00152 (STAiR18-A) and MIR4435-2HG (STAiR18-B) loci show different exonic patterns. The duplication comprises 200 kb of the 5ˈ-region containing exon 1–4 of STAiR18, exons 5–9 are unique (hg19). **c** Locus-specific expression of STAiR18 in IL-6-treated or withdrawn INA-6 cells measured by qPCR using either locus-specific primers (STAiR18-A and STAiR18-B) or a primer pair detecting transcripts from both loci. Values were normalized to U6 RNA (*n* = 3). **d** Determination of STAiR18 copy number per cell. A serial dilution of pcDNA plasmid containing STAiR18 and reverse transcribed RNA from 1 × 10^4^ INA-6 cells were subjected to qPCR using the STAiR18 (both) primer pair. A logarithmic regression was added, enabling the determination of STAiR18 copy number by Ct value (*n* = 3). **e** Determination of STAiR18 Half-Life (HL) by treating INA-6 cells with ActinomycinD (ActD) for indicated durations, followed by RNA isolation, RT and qPCR using intron-spanning primers for STAiR18, STAT3 and U6. Values were adjusted to U6 RNA and normalized to the DMSO control (*n* = 3). HL was determined by a polynomic regression

Survival of INA-6 cells strictly depends on IL-6, which in turn activates the transcription factor STAT3 (see Additional file 1: Figure S3). However, former transcriptome studies revealed that protein coding STAT3 target genes are not involved in survival regulation in these cells. Therefore, the focus was set on identifying long noncoding RNAs induced by STAT3 in INA-6 cells in our previous work. One of those RNAs called STAiRs, STAiR18, was shown to be expressed from the MIR4435-2HG locus on chromosome 2 [8] and will be analyzed here more in detail. Furthermore, by examining the sequence conservation, we discovered the STAiR18 locus to be present in all mammalian genomes. Interestingly, this locus showed a large degree of duplication on chromosome 2 exclusively for the human genome (see Fig. 1a and Table 1). While the original STAiR18, annotated as MIR4435-2HG, is located on the chromosome 2 minus strand, the duplicated STAiR18 locus was detected on the chromosome 2 plus strand and is annotated as LINC00152 alias CYTOR. Public databases like the UCSC Genome Browser also suggest a number of spliced transcripts originating from both loci (Fig. 1b). Closer inspection of mammalian genomes further revealed that the duplication event took place in the human lineage approximately half-way between the Modern Human and the Human/Chimpanzee ancestor (see Additional file 1: Table S5 and S6 for details).

**Table 1:** Coordinates of the STAiR18 duplication (hg19)

lncRNA name	Annotated name / alias	hg19 coordinates	Length	Strand
STAiR18-A	LINC00152 / CYTOR	chr2:87,754,946-87,821,037	66 kb	Plus
STAiR18-B	MIR4435-2HG / LOC541471	chr2:111,953,444-112,252,738	300 kb	Minus

Initially, the IL-6-induced expression of STAiR18 was verified by qPCR using a primer pair spanning exon 1 and 2 to detect transcripts from both loci (Fig. 1c). In a previous publication [8], we further demonstrated that the induction of STAiR18 depends on STAT3 as STAT3 knockdown reduced STAiR18 expression in INA-6 multiple myeloma cells. It is worth mentioning, however, that this observation does not allow to decide whether STAiR18 is an immediate or indirect target of STAT3 regulation. Moreover, we estimated the STAiR18 copy number per cell by a plasmid standard dilution resulting in 52 copies per INA-6 cell (Fig. 1d). In contrast, with 168 copies, the strong IL-6-inducible gene cyclophillin B (cypB) was only three times more abundant (see Additional file 1: Figure S4). Since ncRNAs are expressed at lower levels than mRNAs, it is assumed that STAiR18 plays an important role within the cell [14]. To get an impression of STAiR18 RNA stability and turnover, STAiR18 half-life was analyzed by blocking its transcription with ActinomycinD (ActD). A STAiR18 half-life of 1.6 h was identified (Fig. 1e), which is similar to the half-life determined for STAT3 mRNA. To exclude physiological effects of ActD and DMSO on INA-6 cells, we confirmed cell vitality in parallel (see Additional file 1: Figure S5).

### Multiple splice isoforms originate from both STAiR18 loci

**Fig. 2:**
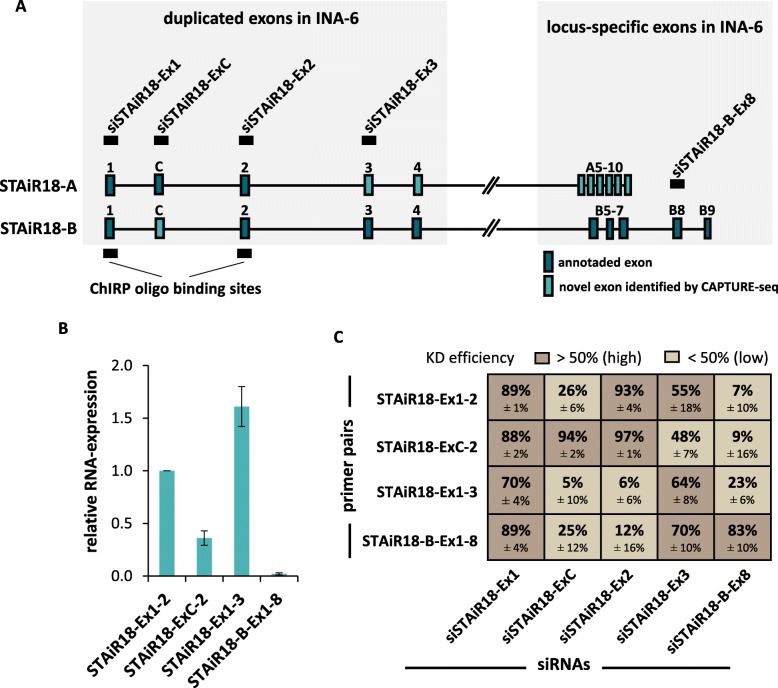
STAiR18 transcript architecture in INA-6 cells. **a** Schematic representation of STAiR18 transcript variants identified by CAPTURE-RNA-sequencing. Already annotated exons are shown in dark blue, novel exons in light blue. Exons targeted by the siRNAs and regions covered by the biotinylated oligonucleotides are marked at the top and bottom of the scheme, respectively. **b** Validation of abundant STAiR18 splice variants in INA-6 by qPCR using transcript-specific primers pairs. Values were normalized to U6 RNA (*n* = 4). **c** Knockdown (KD) of STAiR18 isoforms in INA-6 cells using siRNAs targeting STAiR18 exons (Figure 2a) and a negative control siRNA. KD efficiency was determined 48 h after KD by qPCR using intron spanning primer pairs. Values were normalized to U6 RNA (*n* = 3)

To identify multiple myeloma-specific STAiR18 transcripts, we used biotinylated DNA-oligonucleotide probes complementary to exon 1 and exon 2 to pull down preferentially spliced STAiR18 RNA transcripts from INA-6 cells and subjected them to next-generation sequencing (CAPTURE-seq). Of note, the probes captured the short, spliced RNAs with higher efficiency than the long macroRNA transcripts (data not shown). Hence, CAPTURE-RNA-sequencing data under-represented the levels of unspliced variants. However, when expression levels were compared by qPCR, a well-measurable amount of unspliced transcripts could be detected (data not shown). As shown in Additional file 1: Figure S6, almost all transcript variants already annotated were detected. Additionally, we identified as of yet unknown variants and verified their presence by qPCR analyses using intron-spanning primers, as demonstrated in Fig. 2a and b. Our CAPTURE-seq data furthermore suggested that both STAiR18 variants (A + B) are transcriptionally active. STAiR18-A and STAiR18-B share exons 1 to 4, which show strong sequence homology. RT-qPCR using locus-specific primers (Fig. 1c) as well as amplicon sequencing of the intron-spanning PCRs (data not shown) demonstrated a comparable STAiR18 expression from both loci. The successful usage of oligo-d(T) primers for reverse transcription further revealed that these transcripts are polyadenylated (see Additional file 1: Figure S7). By alternative splicing a wide variety of different isoforms are produced from both loci. The most abundant spliced transcripts comprise exon 1 plus either exon 2 or 3 (Fig. 2b). Furthermore, verified STAiR18 isoforms were confirmed by STAiR18 knockdown experiments. For example the transcript variant STAiR18-Ex1–3 was targeted by both siSTAiR18-Ex1 and siSTAiR18-Ex3 (siRNA binding sites are shown in Fig. 2a, and knockdown efficiencies in Fig. 2c). Additional to the four main transcript variants (see Fig. 2b), these knockdowns suggested the existence of three additional isoforms, which can be explained as follows: The isoform STAiR18-Ex1–2 was targeted by siSTAiR18-Ex1 and siSTAiR18-Ex2 as expected, but also by siSTAiR18-Ex3. This indicated the presence of an isoform STAiR18-Ex1–2-3. The same is true for STAiR18-ExC-2, which might also include the first exon as well as STAiR18-B-Ex1–8, which also contains exon 3.

### STAiR18 is a survival key player for myeloma cells

**Fig. 3:**
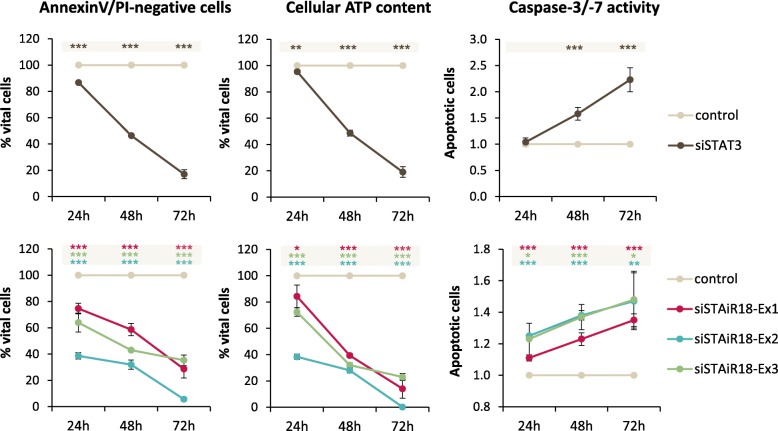
STAiR18 knockdown affects the vitality of INA-6 cells. Permanently IL-6-treated INA-6 cells were transfected with siRNAs targeting STAiR18 exons 1, 2 and 3, as well as a negative control siRNA. To compare the knockdown effects on cell vitality, a STAT3 knockdown was also conducted. Cell vitality was analyzed by determining Annexin-V- and PI-stained cell populations via flow cytometry (first panel), cellular ATP content using the CellTiter-Glo-kit (middle panel) and Caspase3-assay (third panel) after 24 h, 48 h and 72 h (*n* = 3). The indication of significances refers to the control

The STAiR18 phenotype in INA-6 cells was studied by knockdown experiments in combination with different vitality assays (see Fig. 3). We used siRNAs targeting STAiR18 exons 1, 2, and 3, which are common to both loci. Figure 2c shows a matrix of knockdown efficiencies for all siRNAs over primer pairs representing various splice variants.

The knockdown of STAT3 was carried out as a control as the cells are expected to undergo apoptosis. Targeting STAiR18 with different siRNAs comparably reduced cell vitality and induced apoptosis in INA-6 cells. In this respect, STAiR18 knockdown was as efficient as knockdown of STAT3. To exclude off-target effects, we used additional, distinct siRNA oligonucleotides targeting exons 1 and 2 in multiple positions. Both induced apoptosis to a comparable extent (Additional file 1: Figure S8). In conclusion, the major STAiR18 splice variants are essential for survival in INA-6 myeloma cells. This effect was also observed in other multiple myeloma cell lines (U266, MM1S, JK6E), indicating that STAiR18 plays a general role in mediating survival of myeloma cells (Additional file 1: Figure S9).

### STAiR18 and STAT3 share a set of regulated target genes

**Fig. 4:**
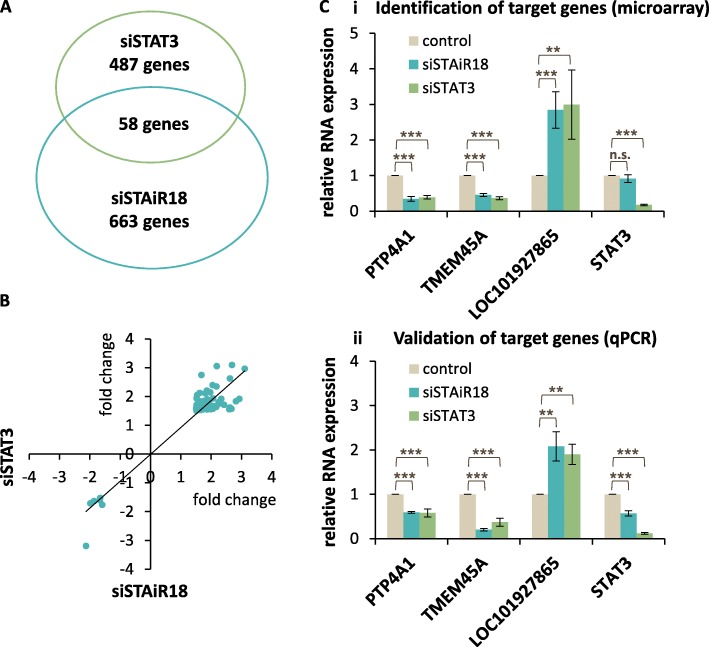
Identification of genes regulated by STAiR18 and STAT3. **a** Differentially regulated STAT3 and STAiR18 target genes determined after STAiR18-Ex1 and STAT3 KD (*n* = 4). RNA was isolated 40 h post-transfection and subjected to gene expression microarrays. A minimal fold-change of 1.5-fold and maximum *p*-value of 0.05 were applied as cutoff criteria, yielding 545 and 721 differentially regulated candidates upon STAT3 and STAiR18 knockdown, respectively. 58 of these candidates are regulated by both knockdowns. **b** The fold-changes of 58 genes differentially regulated by both the KD of STAT3 and STAiR18 were plotted against each other. **c** Validation of selected transcripts regulated by both KDs by qPCR using specific primer pairs. Values were normalized to U6 RNA (*n* = 4). The detected expression of equivalent genes identified by microarray is shown at the top panel (i) for comparison

In INA-6 cells the survival phenotype of STAiR18 is highly similar to that of STAT3. To further investigate this observation, cells were transfected with siRNAs targeting either STAiR18 exon 1 or STAT3 mRNA, and with an siRNA control. Subsequently, transcription patterns were analyzed by microarrays comprised by probes to all mRNAs as well as a substantial number of ncRNAs, in order to identify genes regulated by both STAT3 and STAiR18. Differentially expressed transcripts are given in Additional file 1: Tables S7 and S8, respectively, and all data sets were deposited in the GEO database (accession no. GSE71092). With a minimum fold-change of 1.5 and a maximum *p*-value of 0.05, we observed 545 and 721 transcripts to be differentially regulated by STAT3 and STAiR18 knockdown, respectively. Interestingly, 58 of these transcripts were regulated by both knockdowns (Fig. 4a and Additional file 1: Table S9). Up- and down-regulation of all 58 common transcripts occurred in the same direction by STAT3 and STAiR18 (Fig. 4b). The overlap was even higher when all genes significantly regulated by at least one knockdown were plotted against each other. As shown in Additional file 1: Figures S10A and B, almost all genes were regulated in the same direction. As a control, this phenomenon was not generally observed upon plotting all genes (Additional file 1: Figure S10C). For selected RNAs, the microarray results were verified by RT-qPCR (Fig. 4c). Of note, in this connection STAT3 mRNA was found to be down-regulated upon STAiR18 knockdown. Hence, this observation suggests a tight regulatory interplay between STAiR18 and STAT3 on a transcriptional level.

### STAiR18 interacts with STAT3 pre-mRNA

**Fig. 5:**
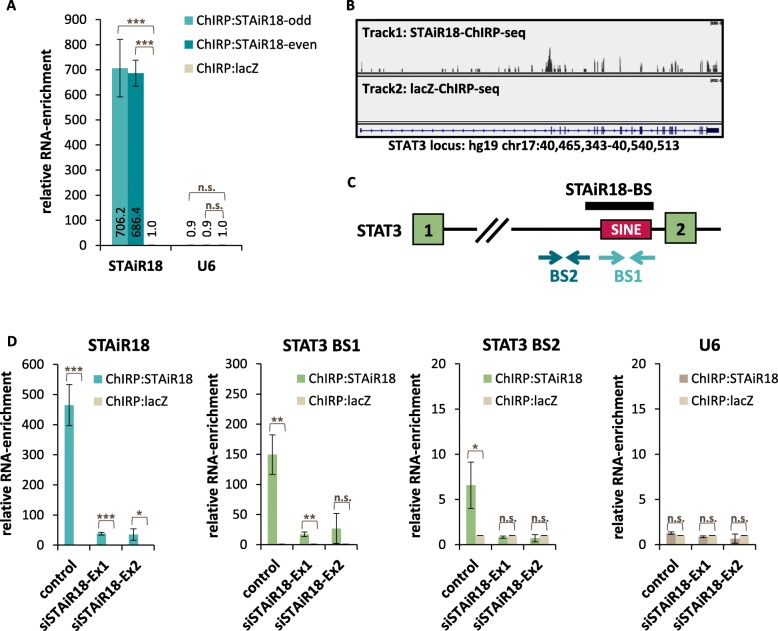
STAiR18 interacts with STAT3 mRNA. **a** STAiR18 ChIRP is highly specific and efficient. Pulldown of STAiR18-RNA from crosslinked INA-6 cells was realized by oligos covering exon 1 and 2. Oligos were then divided into two pools (odd and even numbered). For control, oligos targeting lacZ RNA were used. After pulldown, RNA was analyzed by qPCR (*n* = 4). **b** STAiR18 ChIRP followed by RNA-sequencing revealed an association of STAiR18 with STAT3 on RNA-level. Shown is the IGV window of the STAT3 locus after STAiR18 (Track1 with STAiR18 binding peak) and lacZ (Track2) ChIRP-seq. **c** The STAT3 gene harbors a SINE within the first intron corresponding to the binding site (BS) of STAiR18. Binding sites for primers marking the specific STAiR18-BS1 are shown in light blue, a primer pair for a upstream region BS2 is shown in dark blue. **d** Specific interaction of STAiR18 with STAT3 RNA was determined in INA-6 cells after KD of STAiR18 exon1 or 2, followed by a ChIRP experiment using oligonucleotides targeting STAiR18 or lacZ 24 h post-transfection. RNA was analyzed by qPCR using specific primers targeting the STAiR18-BS within the STAT3 intron. Primer pairs for STAiR18 were used as positive, for U6 as negative control. Values were normalized to lacZ (*n* = 3)

Given that STAiR18 seems to regulate STAT3 RNA, we asked whether both RNA molecules may interact with each other. Therefore, ChIRP was conducted using oligonucleotides targeting STAiR18 exon 1 and 2 in order to pull down STAiR18 together with RNAs bound to it. The successful enrichment of STAiR18 RNA by two oligo pools (separated into even and odd oligos) over lacZ negative control oligos is shown in Fig. 5a. Thus, these oligos (even and odd combined) were used to additionally enrich STAiR18-bound RNAs followed by an identification by RNA sequencing. Our ChIRP RNA-seq data indeed revealed an association of STAiR18 with a SINE element within the first intron of STAT3 primary mRNA (Fig. 5b + C and Additional file 1: Table S10). Verification of this association by RT-qPCR yielded a remarkably high enrichment using a primer pair specifically designed for the binding site within the SINE (BS1) compared to the lacZ control (Fig. 5d), suggesting a particularly tight interaction between the two RNAs. An upstream primer pair (BS2) still showed a specific enrichment but less efficiently. Of note, RNA complexes are sonicated and hence fragmented during the ChIRP process. Thus, only those regions of target RNAs that are involved directly or indirectly in complex formation are expected to be detected by this procedure. In all cases, a STAiR18 knockdown further confirmed the specificity of STAiR18-STAT3-RNA-interaction. Additionally, no U6 control RNA could be detected after STAiR18 ChIRP, proving the specificity of this pulldown.

### Positive regulatory feedback between STAT3 and STAiR18

**Fig. 6:**
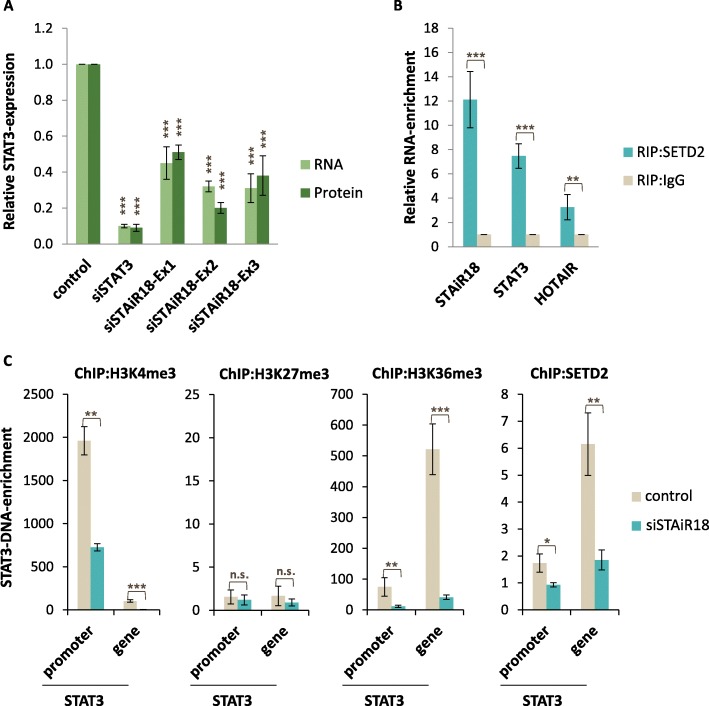
STAiR18-mediated regulation of STAT3. **a** STAT3 mRNA and protein levels depend on STAiR18. KDs of STAiR18 and STAT3 were conducted in INA-6 cells, followed by RNA- and protein-isolation 24 h and 48 h, respectively. RNA expression was carried out by qPCR (*n* = 4); protein expression by SDS-PAGE and immunoblotting using a densiometric analysis (*n* = 4). The indication of significances refers to the control. The corresponding Western Blots are displayed in Additional file 1: Figure S9. **b** SETD2 is associated with STAiR18 and STAT3 mRNA. INA-6 cells were crosslinked and a RIP was performed using a specific SETD2 antibody together with an IgG negative control. RNA was analyzed by qPCR. Values were normalized to IgG control (*n* = 3). **c** Active chromatin state of the STAT3-locus depends on STAiR18. STAiR18 KD (targeting exon2) followed by ChIP was performed in INA-6 cells using antibodies targeting H3K4me3, H3K27me3, or H3K36me3, and SETD2 24 h after transfection. IgG was used as a negative control. Enriched DNA was isolated and analyzed by qPCR with specific primers for the STAT3 promoter region and the STAT3 gene. Values were normalized to the corresponding IgG negative control (*n* = 4)

As shown in Fig. 4c (ii), STAT3 mRNA is down-regulated upon STAiR18 knockdown, suggesting a positive feedback between both molecules. To substantiate this further, we transfected siRNAs against STAiR18 exons 1, 2 and 3 into INA-6 cells and subsequently analyzed STAT3 mRNA and protein levels. We observed a significant reduction of both STAT3 mRNA and protein (Fig. 6a), albeit with slightly different efficiencies. STAT3 mRNA down-regulation was most effective upon targeting STAiR18 exons 2 and 3, which was confirmed by Western blot (see Additional file 1: Figure S11).

As the regulation of STAT3 by STAiR18 seems to occur at the level of transcription (6A) and an association of STAiR18 with nascent STAT3 mRNA within the first intron could be observed, we next asked whether STAiR18 might influence the epigenetic status of the STAT3 locus. Thus, ChIP experiments in INA-6 cells revealed that H3K4 trimethylation of the STAT3 promoter and H3K36 trimethylation in the transcribed region were significantly reduced after STAiR18 knockdown (Fig. 6c). H3K27 trimethylation was hardly detectable at the STAT3 locus. Given that H3K36 trimethylations are altered most within the STAT3 locus by STAiR18, we asked whether the histone methyltransferase SETD2, which is responsible for H3K36 trimethylations, may be involved in this process. First, a RIP experiment confirmed an association of SETD2 protein with nascent STAiR18 and STAT3 RNA, as expected (Fig. 6b). Moreover, the amount of STAT3 DNA enriched by SETD3 ChIP was reduced after STAiR18 knockdown, indicating that STAiR18 might facilitate the active chromatin state of the STAT3 locus via SETD2. This suggests that STAiR18 is essential for sustaining a transcriptionally active epigenetic status at the STAT3 locus.

**Fig. 7:**
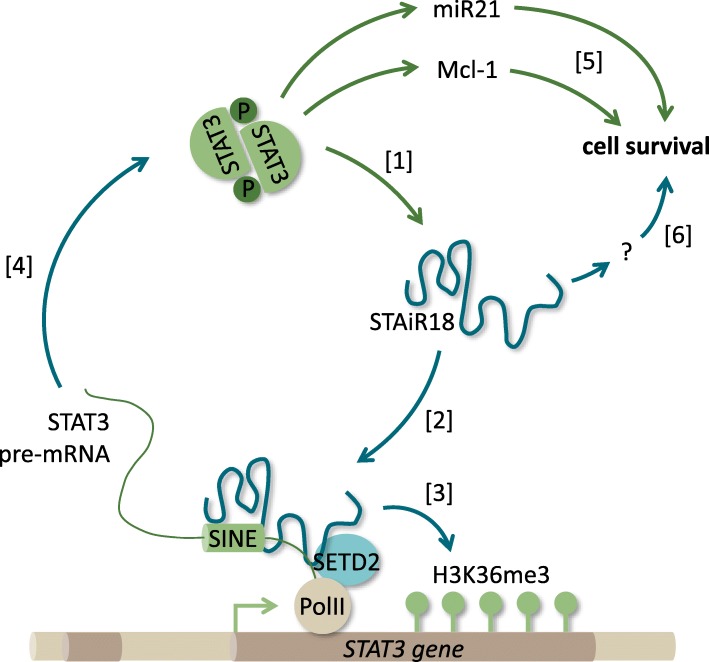
Schematic representation of STAiR18’s role in INA-6 cells. [1] STAT3 induces STAiR18 expression. [2] STAiR18 interacts with STAT3 primary RNA within a SINE element. [3] Additionally, STAiR18 is involved in maintaining H3K36-trimethylation of the STAT3 locus, probably via SETD2. [4] This results in a positive feedback, yielding elevated STAT3 levels. Both STAT3 and STAiR18 ensure survival of multiple myeloma cells. [5] STAT3 triggers survival in part via miR21 [6] and Mcl-1 [5]. [6] However, the mechanism of STAiR18-mediated myeloma cell survival remains to be defined

In summary, STAT3 and STAiR18 show a tight regulatory interaction on multiple levels. The activated transcription factor STAT3 induces STAiR18 expression in INA-6 myeloma cells. In turn, STAiR18 directly binds a short interspersed nuclear element (SINE) within the first intron of STAT3 pre-mRNA. Since STAiR18 is essential to induce or maintain H3K36 trimethylations as activating histone marks in the STAT3 locus, we suggest an interaction with the histone-modifying complex SETD2, which is indeed regulated by STAiR18 within the STAT3 locus. Furthermore, we demonstrated that STAT3 expression on RNA and protein levels is increased by STAiR18, constituting a positive feedback loop between both molecules. Therefore, the tight interplay of STAT3 and STAiR18 as well as the regulation of their downstream targets turns out to be essential for multiple myeloma cell survival. As a mediator of survival, STAT3 conveys this effect largely by the miR21 [6] and, less efficiently, by Mcl-1 [5]. However, there might be other mechanisms, by which STAT3 and STAiR18 promote myeloma cell survival and which remain to be elucidated (Fig. 7).

## Discussion

LINC00152, which we later termed STAiR18 due to its STAT3-dependent expression [8], was identified in 2013 via expression profiling in gastric cancer. Now, almost 80 publications are available describing this ncRNA to be overexpressed in different cancer types, predominantly in gastric, lung and colon cancer, thus implicating the major role of STAiR18 in tumor regulation as well as a general interest in this ncRNA. In these analyses, STAiR18 was described to regulate cell cycle arrest, apoptosis, EMT, migration and invasion, predominantly by its ability to sponge distinct microRNAs.

Here, we focused on STAiR18 in multiple myeloma and initially found the LINC00152 locus to be duplicated on chromosome 2 within the human genome (first published by Diederichs in HeLa 2017). Furthermore, the duplication of the STAiR18 locus appears particularly intriguing as it occurred very recently during the evolutionary development of *Homo sapiens* from its primate ancestors. Using locus-specific PCR primers, we demonstrated that both loci are active and seem to produce similar yet not identical splice patterns. It is intriguing to speculate that the highly conserved STAiR18 provides a mechanistic principle that can be dynamically modulated by more rapidly evolving additional exons during phylogeny, and that the duplication further extends this functional plasticity in the human species. Many splice variants are derived from the STAiR18 loci, as demonstrated by CAPTURE-RNA-sequencing. Exons 1 and 2 show phylogenetic conservation in all mammals, while conservation of other exons seems restricted to primates. This suggests an evolutionarily preserved functional role of STAiR18.

As mentioned above, investigations of other groups revealed that LINC00152 (STAiR18-A transcript variant) shows elevated expression or hypomethylation [15] in various cancer types, mainly gastric cancer, indicating its general role in tumorigenesis or tumor maintenance [16]. Knockdown of the two most abundant transcript variants containing exon 1 plus either exon 2 or exon 3 results in a rapid apoptosis in INA-6 cells, as similarly observed after STAT3 knockdown. The same type of response was observed in other multiple myeloma cell lines (U266, MM1S, JK6E). Reliability of knockdowns were ensured by different siRNAs to minimize off-target effects and further, cell vitality, viability and apoptosis were measured by independent tests to prove the survival phenotype of STAiR18. A recent publication also found LINC00152 to regulate proliferation and apoptosis in multiple myeloma, confirming our findings. In this publication, Tianhua et al. claim that LINC00152 acts as a sponge, hence a negative regulator of miR497 [17].

Here, we present a completely undescribed mechanism of LINC00152/STAiR18 in myeloma cells. Microarray studies upon STAT3 and STAiR18 knockdown revealed a highly overlapping set of target genes. This observation suggests that STAiR18 represents a downstream regulator mediating part of the transcriptional responses of STAT3. In fact, closer inspection of our microarray data revealed that those STAT3 targets, which are known to be induced by IL-6-triggered activation of the transcription factor, including SOCS3, JunB, GADD45β, Pim-1, and others, do not appear to be amongst the STAiR18 targets. In contrast, STAT3 and STAiR18 seem to primarily share target genes such as those that are regulated upon STAT3 knockdown but not via STAT3 tyrosine phosphorylation. In fact, STAT3 has been shown to regulate a number of target genes and functions without being tyrosine phosphorylated [18, 19]. Thus, STAiR18 might be responsible for mediating alternative STAT3 downstream pathways that either use unphosporylated STAT3 or the low, basic phosphorylation levels maintained by permanent cytokine stimulation, while strong cytokine signals use the classical STAT3 tyrosine phosphorylation pathway. In addition, cell vitality and gene expression analyses showed that the STAiR18 effects exceed the STAT3 effects. Therefore, we are convinced that STAiR18 assumes STAT3-independent functions. Nevertheless, functions of both, STAiR18 and STAT3, ensure survival of myeloma cells.

Amongst the genes directly regulated by STAiR18 and STAT3 we identified several targets involved in cell cycle progression and cancer cell survival, like the protein tyrosine phosphatase type 4 member 1 (PTP4A1) [20–22] and the transmembrane protein 45A (TMEM45A) [23, 24]. Interestingly, STAT3 itself was found to be a downregulated target upon STAiR18 knockdown, indicating a positive feedback loop between both molecules. STAiR18 knockdown not only reduced STAT3 expression at the mRNA and protein level, but it also activates or maintains a positive and openly transcribed chromatin status of the STAT3 locus in INA-6 cells. Our data demonstrate that within the STAT3 gene, H3K36 trimethylation was drastically reduced upon STAiR18 knockdown. To our knowledge, this is the first study demonstrating a role of LINC00152/STAiR18 in chromatin activation. Thus, it is intriguing to speculate that STAiR18, by binding to the nascent STAT3 mRNA, recruits histone modifying enzymes to the STAT3 locus, which are involved in maintaining an open and transcriptionally active chromatin state. We show here that STAiR18 interacts directly with a Short Interspersed Nuclear Element (SINE) located in the STAT3 pre-mRNA. These data are in line with previous reports demonstrating an interaction of ANRIL, another lncRNA, with SINEs. ANRIL regulates cell proliferation, adhesion, and apoptosis in arteriosclerosis by recruiting polycomb proteins to promoters of target genes containing SINEs [25]. This mechanism may be transferable to other STAiR18 target genes, like PTP4A1 and TMEM45A, for which we also observed a direct interaction with STAiR18 (Additional file 1: Figure S12) as well as a reduced expression after STAiR18 knockdown (Fig. 4c). Taken together, these findings substantiate a tight interplay of STAT3 and STAiR18, resulting in persistent survival of multiple myeloma cells.

## Conclusion

Survival of INA-6 multiple myeloma cells depends on IL-6-mediated STAT3 activation. STAT3 as a transcription factor induces the expression of the ncRNA STAiR18 (LINC00152, CYTOR), which in turn facilitates a positive feedback to STAT3. Moreover, both factors are strong survival key players in myeloma cells. In conclusion, the interplay of both molecules unravels an enthralling potential mechanism.

## Additional file

**Additional file 1: Figure S1.** Vector map pcDNA 3.1 (+)-STAiR18. **Figure S2.** Vector map pcDNA 3.1 (+)-cypB. **Figure S3.** Survival of INA-6 cells depends on IL-6-induced STAT3 activation. **Figure S4.** Determination of CyclophillinB copy number. **Figure S5.** INA-6 cell vitality after ActinomycinD treatment. **Figure S6.** Identification of STAiR18 isoforms by capture RNA-sequencing. **Figure S7.** STAiR18 polyadenylation. **Figure S8.** Survival of INA-6 myeloma cells depends on STAiR18. **Figure S9.** Survival of myeloma cells depends on STAiR18. **Figure S10.** Comparison of genes regulated by STAT3 and STAiR18 knockdown. **Figure S11.** STAT3 protein levels are STAiR18 regulated. **Figure S12.** STAiR18 associates with specific RNA and DNA targets. **Table S1.** (q) PCR primers. **Table S2.** Stealth siRNAs. **Table S3.** Antibodies. **Table S4.** ChIRP oligonucleotides. **Table S5.** Duplication of STAiR18 in the Neandertal and Denisova genomes. **Table S6.** Absence of STAiR18 duplication in other primate genomes. **Table S7.** Genes regulated by STAT3 knockdown. **Table S8.** Genes regulated by STAiR18 knockdown. **Table S9.** Genes regulated by STAT3 and STAiR18 knockdown. **Table S10.** RNA interaction partners of STAiR18.

## Data Availability

The data sets supporting the results of this article are available in the GEO database repository [GEO:GSE71092], [GEO:GSM2496675], [GEO:GSM2496676], [GEO:GSM2496682], [GEO:GSM2496683].

## Abbreviations

ActD: Actinomycin D
Alu: Arthrobacter luteus
BS: Binding site
ChIP: Chromatin Immunoprecipitation
ChIRP: Chromatin Isolation by RNA Purification
CypB: Cyclophillin B
CYTOR: Cytoskeleton regulator RNA
GADD45β: Growth arrest and DNA-damage-inducible beta
GEO: Gene Expression Omnibus
H3K27me3: Histone 3 – lysine 27 – trimethylation
H3K36me3: Histone 3 – lysine 36 – trimethylation
H3K4me3: Histone 3 – lysine 4 – trimethylation
IL-6: Interleukin-6
INA-6: Multiple myeloma cell line
JAK: Janus kinase
JunB: Transcription factor jun-B
lncRNA: Long noncoding RNA
miRNA: MicroRNA
ncRNA: Noncoding RNA
Pim-1: Serine/threonine-protein kinase Pim-1
PTP4A1: Protein tyrosine phosphatase type IVA 1
SETD2: SET domain containing 2
SINE: Short interspersed nuclear element
SOCS3: *Suppressor of cytokine signaling 3*
STAiR18: STAT3-induced ncRNA 18
STAT3: *Signal transducer and activator of transcription 3*
TMEM45A: Transmembrane protein 45A
U266: Multiple myeloma cell line
UCSC: University of California Santa Cruz
