# Supplementary material for: Master and servant: LINC00152 – a STAT3-induced long noncoding RNA regulates STAT3 in a positive feedback in human multiple myeloma
Source: BMC Med Genomics. 2020 Feb 10;13:22. doi: 10.1186/s12920-020-0692-3 (PMC7011539; doi:10.1186/s12920-020-0692-3)
Supplement: Supplementary file 1 — Additional file 1: Figure S1. Vector map pcDNA 3.1 (+)-STAiR18. Figure S2. Vector map pcDNA 3.1 (+)-cypB. Figure S3. Survival of INA-6 cells depends on IL-6-induced STAT3 activation. Figure S4. Determination of CyclophillinB copy number. Figure S5. INA-6 cell vitality after ActinomycinD treatment. Figure S6. Identification of STAiR18 isoforms by capture RNA-sequencing. Figure S7. STAiR18 polyadenylation. Figure S8. Survival of INA-6 myeloma cells depends on STAiR18. Figure S9. Survival of myeloma cells depends on STAiR18. Figure S10. Comparison of genes regulated by STAT3 and STAiR18 knockdown. Figure S11. STAT3 protein levels are STAiR18 regulated. Figure S12. STAiR18 associates with specific RNA and DNA targets. Table S1. (q) PCR primers. Table S2. Stealth siRNAs. Table S3. Antibodies. Table S4. ChIRP oligonucleotides. Table S5. Duplication of STAiR18 in the Neandertal and Denisova genomes. Table S6. Absence of STAiR18 duplication in other primate genomes. Table S7. Genes regulated by STAT3 knockdown. Table S8. Genes regulated by STAiR18 knockdown. Table S9. Genes regulated by STAT3 and STAiR18 knockdown. Table S10. RNA interaction partners of STAiR18. [file 12920_2020_692_MOESM1_ESM.docx]

Additional file 1

# Legend

1. **Supplemental Figures 2**

S. Figure 1: Vector map pcDNA 3.1 (+)-STAiR18 2

S. Figure 2: Vector map pcDNA 3.1 (+)-cypB 3

S. Figure 3: Survival of INA-6 cells depends on IL-6-induced STAT3 activation 4

S. Figure 4: Determination of CyclophillinB copy number 5

S. Figure 5: INA-6 cell vitality after ActinomycinD treatment 6

S. Figure 6: Identification of STAiR18 isoforms by capture RNA-sequencing 7

S. Figure 7: STAiR18 polyadenylation 8

S. Figure 8: Survival of INA-6 myeloma cells depends on STAiR18 9

S. Figure 9: Survival of myeloma cells depends on STAiR18 10

S. Figure 10: Comparison of genes regulated by STAT3 and STAiR18 knockdown 11

S. Figure 11: STAT3 protein levels are STAiR18 regulated 12

S. Figure 12: STAiR18 associates with specific RNA and DNA targets 13

1. **Supplemental Tables 14**

S. Table 1: (q) PCR primers 14

S. Table 2: Stealth siRNAs 15

S. Table 3: Antibodies 16

S. Table 4: ChIRP oligonucleotides 17

S. Table 5: Duplication of STAiR18 in the Neandertal and Denisova genomes 18

S. Table 6: Absence of STAiR18 duplication in other primate genomes 19

S. Table 7: Genes regulated by STAT3 knockdown 20

S. Table 8: Genes regulated by STAiR18 knockdown 33

S. Table 9: Genes regulated by STAT3 and STAiR18 knockdown 49

S. Table 10: RNA interaction partners of STAiR18 51

## Figure S1: Vector map pcDNA 3.1 (+)-STAiR18


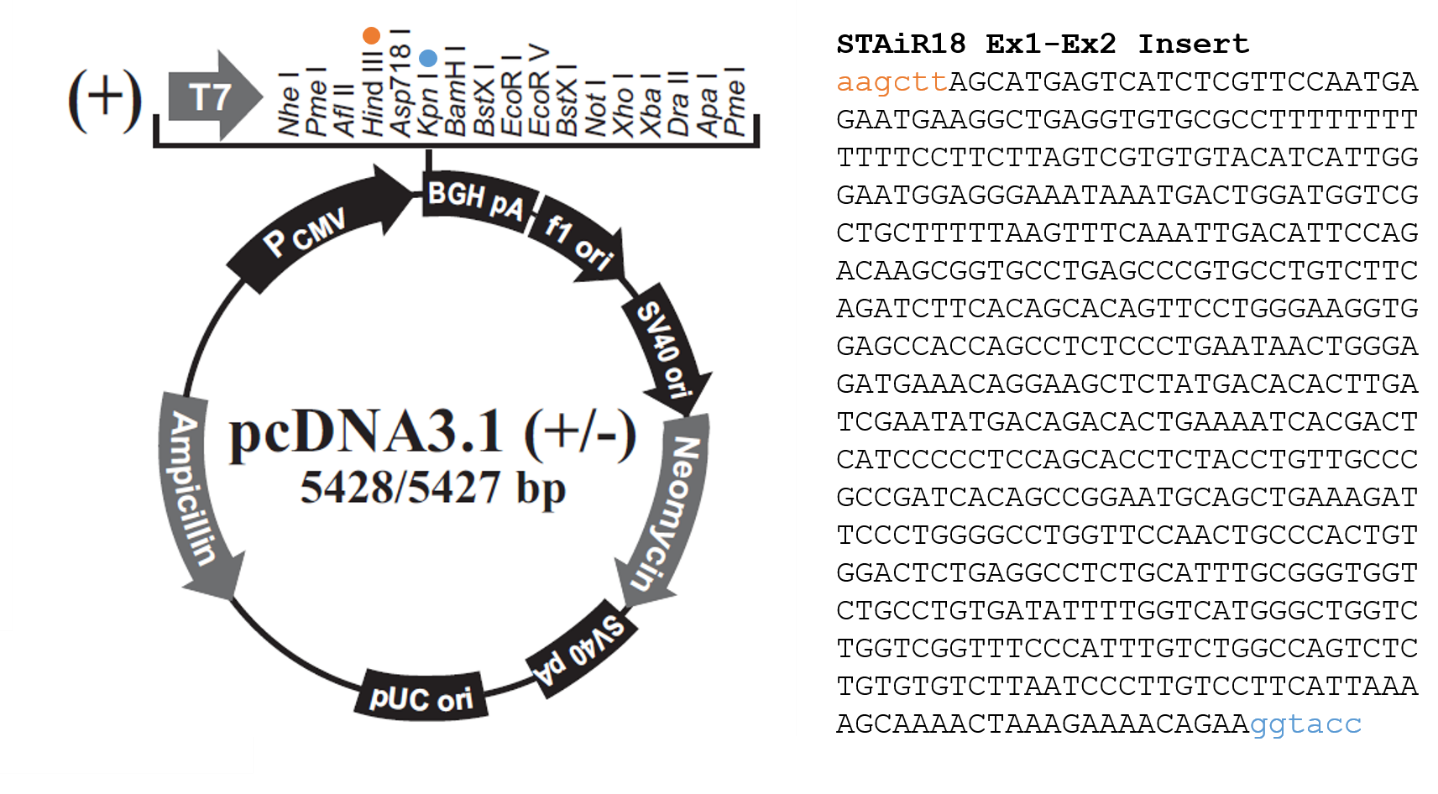


**Figure S1: pcDNA 3.1 (+)-STAiR18 vector map.** The STAiR18 insert consisting of exon 1 and exon 2 is shown right and restriction sites used to transfer the insert into pcDNA plasmid are marked in orange (HindIII) and blue (KpnI).

## Figure S2: Vector map pcDNA 3.1 (+)-cypB


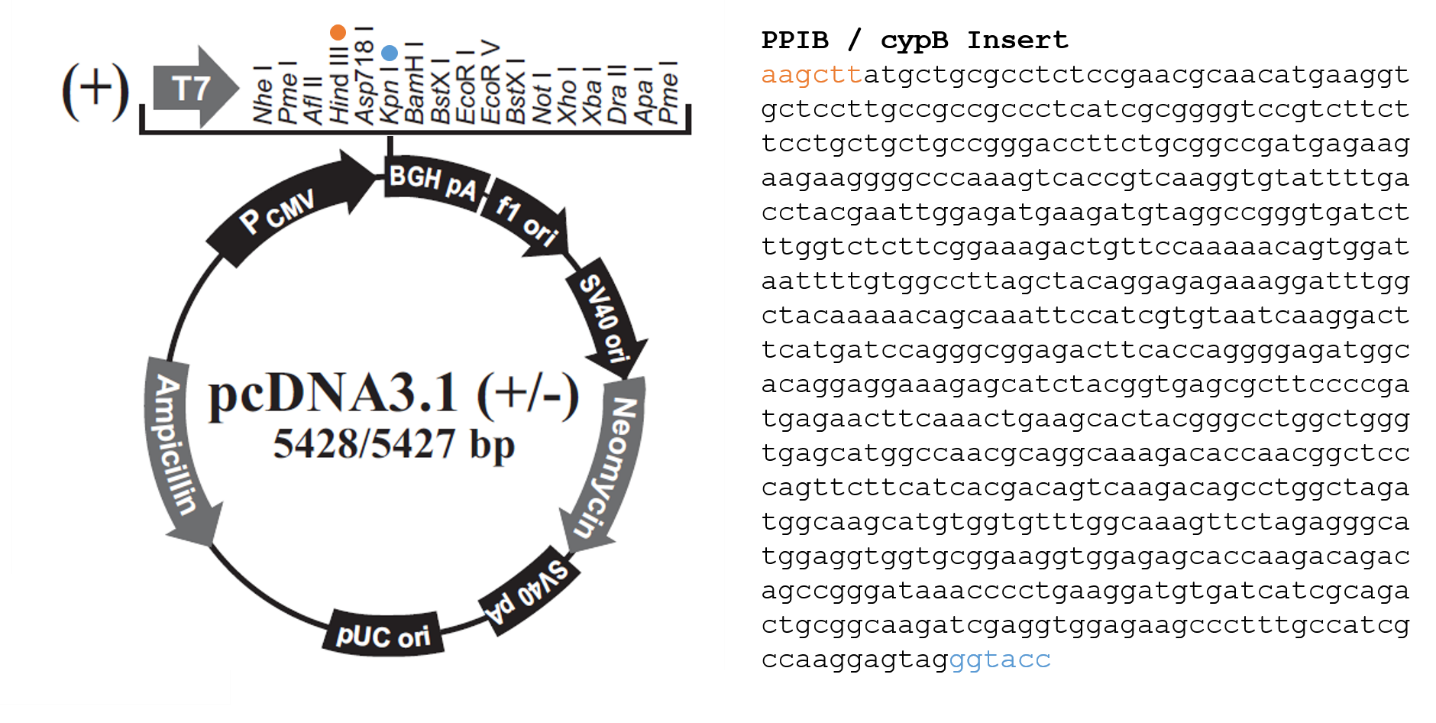


**Figure S2: pcDNA 3.1 (+)-cypB vector map.** The cyclophilinB (cypB) insert is shown right and restriction sites used to transfer the insert into pcDNA plasmid are marked in orange (HindIII) and blue (KpnI).

## Figure S3: Survival of INA-6 cells depends on IL-6-induced STAT3 activation


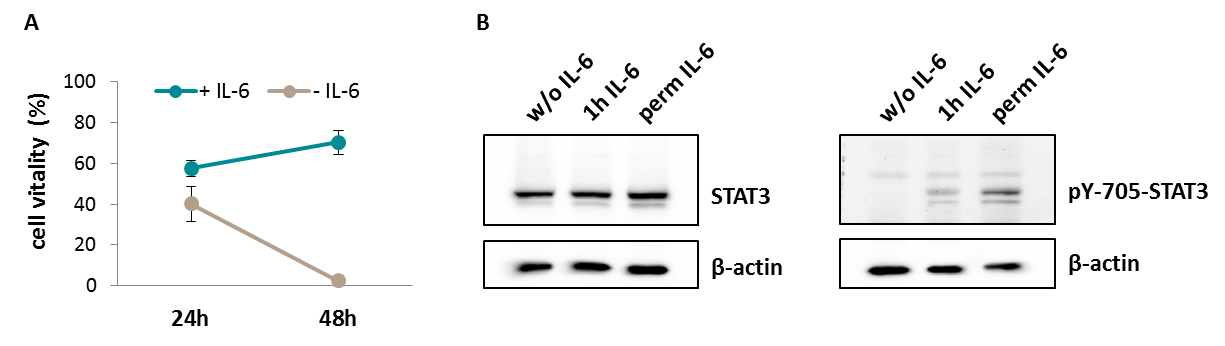


**Figure S3: Survival of INA-6 cells depends on IL-6-induced STAT3 activation.** (A) Survival of INA-6 multiple myeloma cells is IL-6-dependent. INA-6 cells were withdrawn from IL-6 for 48 h (- IL-6) or treated with IL-6 permanently (+ IL-6). Cell vitality was analyzed by the determination of Annexin-V- and PI-stained cell populations by flow cytometry after 24 h and 48 h (n = 3). (B) IL-6-induced STAT3 activation by phosphorylation at tyrosine residue 705. INA-6 cells were permanently cultured with IL-6 (perm IL-6), withdrawn from IL-6 for 12 h (w/o IL-6), and restimulated with IL-6 for 1 h (1 h IL-6). Cells were lysed and protein lysates separated by SDS-PAGE. Phosphorylated STAT3 (pY-705-STAT3), total STAT3, and β-actin were detected by immunoblotting using specific antibodies.

## Figure S4: Determination of CyclophillinB copy number


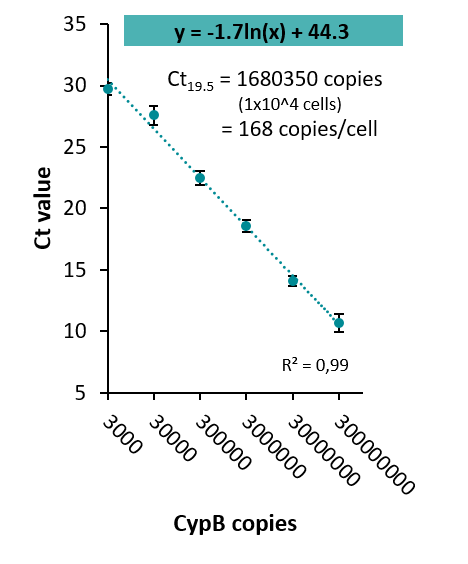


**Figure S4: Determination of CyclophillinB copy number per cell.** A plasmid-based absolute quantification of copy number was conducted. Therefore, Cyclophillin-expressing plasmid (pcDNA_CypB) was serial diluted to copy numbers ranging from 3,000 to 300,000,000 copies. Also, RNA was prepared from 1x10^4^ permanently IL-6 treated INA-6 cells and reverse transcribed into cDNA. Both, the plasmid dilutions and the INA-6 RNA were subjected to qPCR using an intron-spanning CyclophillinB primer pair. Data are expressed as mean ± SD (n = 4). A logarithmic regression was added, enabling the determination of STAiR18 copy number by Ct value.

## Figure S5: INA-6 cell vitality after ActinomycinD treatment


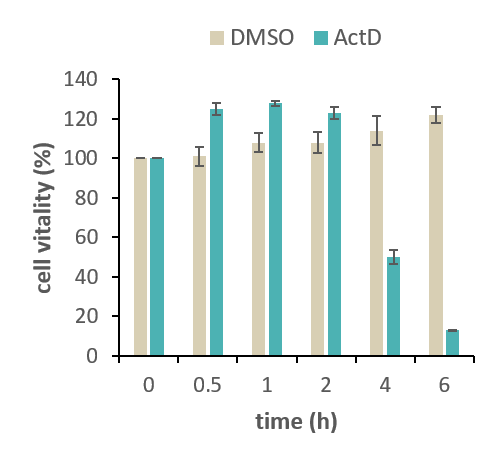


**Figure S5: INA-6 cell vitality after DMSO and ActinomycinD treatment.** 2x10^4^ IL-6-treated INA-6 cells were cultured per well in the presence of 5 µg/ml ActinomycinD in DMSO or plain DMSO for 0 h, 0.5 h, 1 h, 2 h, 4 h and 6 h H . Cell vitality was then determined by Cell Titer Glo Kit (Promega), on the basis of intracellular ATP detected by spectroscopy. Values were normalized to the 0h control and data are expressed as mean ± SD (n = 3).

## Figure S6: Identification of STAiR18 isoforms by capture RNA-sequencing


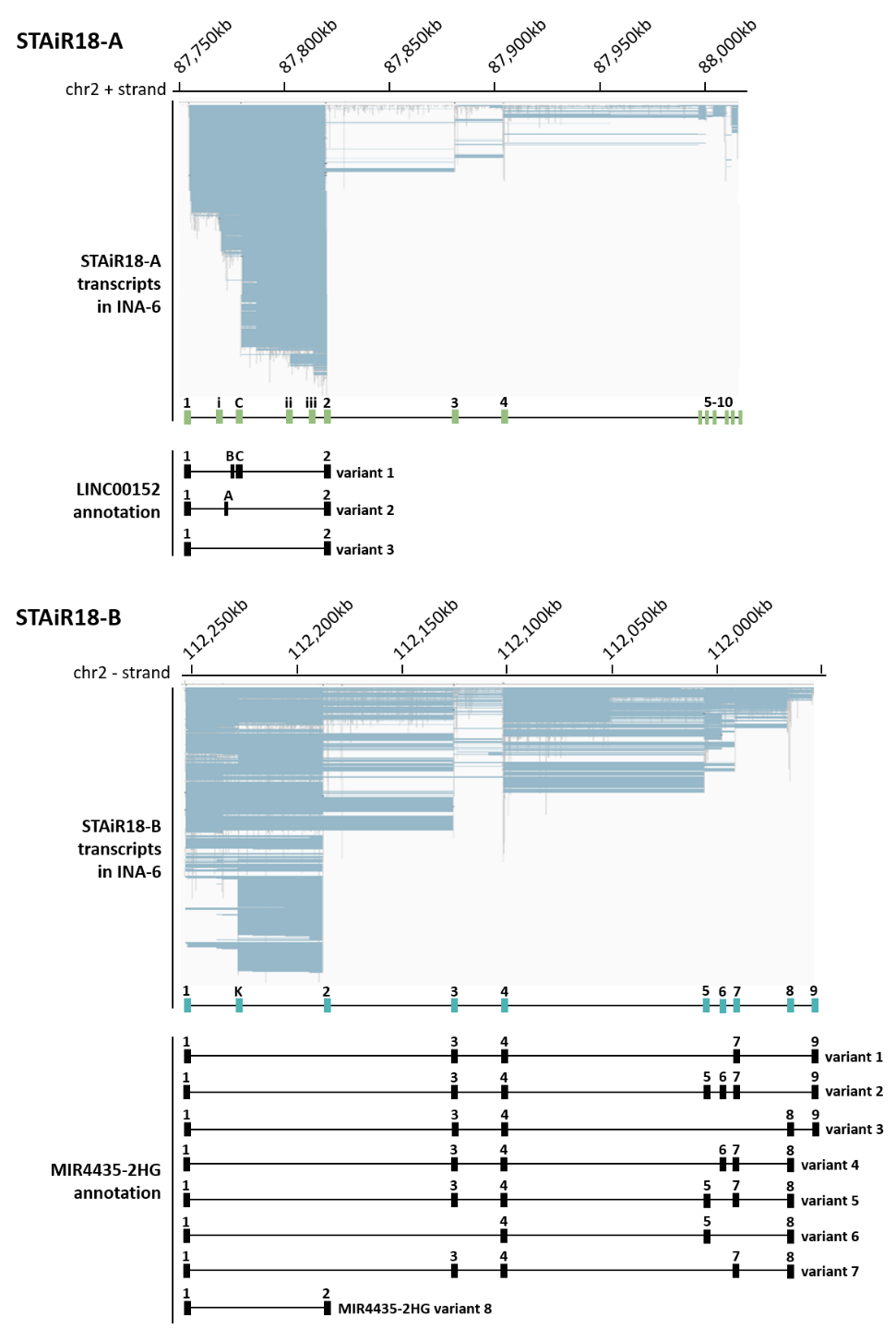


**Figure S6: STAiR18 splice variants in INA-6 cells identified by capture RNA-sequencing.** RNA was isolated from INA-6 cells grown in the presence of IL-6, and was incubated with biotinylated oligonucleotides targeting both STAiR18-A and -B. STAiR18 transcripts were isolated using magnetic streptavidin beads and delivered to next-generation sequencing. Reads were mapped to the human genome in Hg19 by TopHat(21), and visualized using the Integrative genomics viewer.(22) Reads containing splice junctions are shown as blue lines (junctions) flanked by gray boxes (exons). Capture RNA-sequencing data revealed different STAiR18 splice variants in INA-6 cells, schematically shown in blue below the mapped transcripts for both loci separately. Additionally, the annotated transcripts for both, LINC00152 (STAiR18-A) and LOC541471 alias MIR4435‑1HG (STAiR18-B) are shown in black at the bottom.

## Figure S7: STAiR18 polyadenylation

**Figure S7: STAiR18 is polyadenylated.** Total RNA of permanently IL-6 treated (1 ng/ml) INA-6 cells was isolated and DNase-digested. 1 µg RNA each was used for both, a reverse transcription into cDNA with random hexamers and oligo-d(T) primers. cDNAs were subjected to qPCR using specific primers for spliced STAiR18. Expression values were normalized to U6 RNA and compared to the pool reverse transcribed with random hexamers. Data are expressed as mean ± SD (n = 3).

## Figure S8: Survival of INA-6 myeloma cells depends on STAiR18


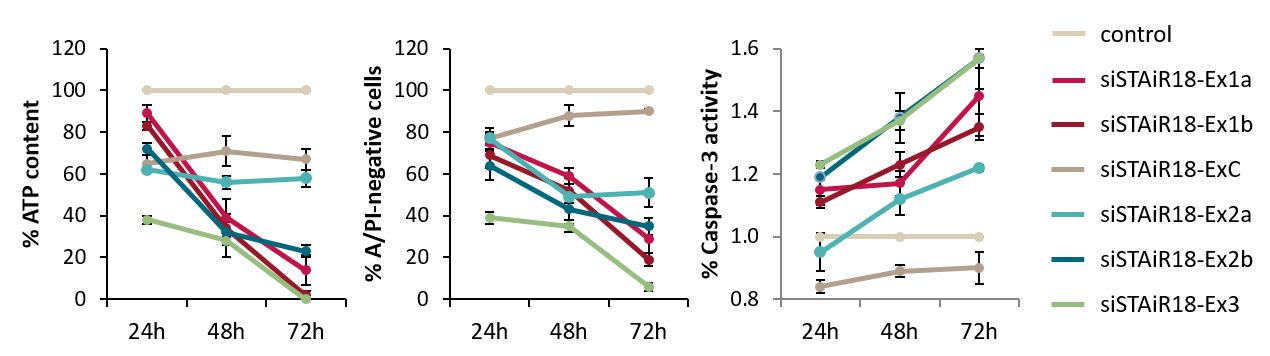


**Figure S8: Survival of INA-6 myeloma cells depends on STAiR18.** Permanently IL-6-treated INA-6 cells were transfected with siRNAs targeting each STAiR18 exon 1 and 2 in two to three different positions as well as targeting exon C and 3 to exclude off target effects. A negative control siRNA was used to compare knockdown effects. Cell vitality was analyzed by determining Annexin‑V- and PI-stained cell populations via flow cytometry (first panel), cellular ATP content using the Glo-kit (middle panel) and caspase3-assay (third panel) after 24 h, 48 h and 72 h (n=3).

## Figure S9: STAiR18 regulates survival in various multiple myeloma cell lines


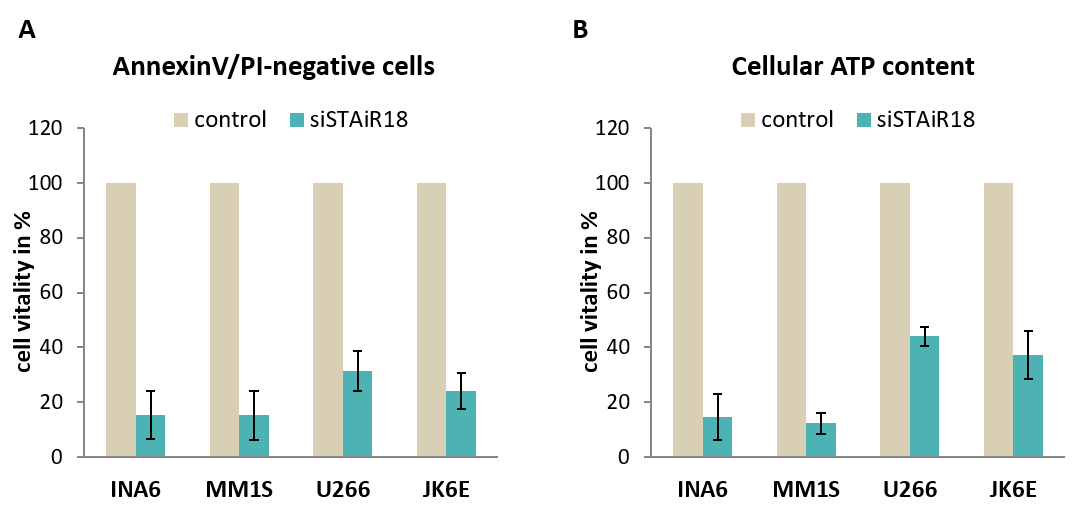


**Figure S9: Vitality of four multiple myeloma cell lines after STAiR18 knockdown.** The multiple myeloma cell lines INA-6 (IL-6-dependent), MM1S (IL-6-independent), U266 (IL-6-independent) and JK6E (IL-6-dependent) were transfected with an siRNA targeting STAiR18 exon 1. A negative-control siRNA was used to normalize knockdown effects. Cell vitality was analyzed by determining Annexin‑V- and PI-stained cell populations via flow cytometry (A) and cellular ATP content using the CellTiter-Glo-kit (B) after 48 h (n = 4).

## Figure S10: Comparison of genes regulated by STAT3 and STAiR18 knockdown

**
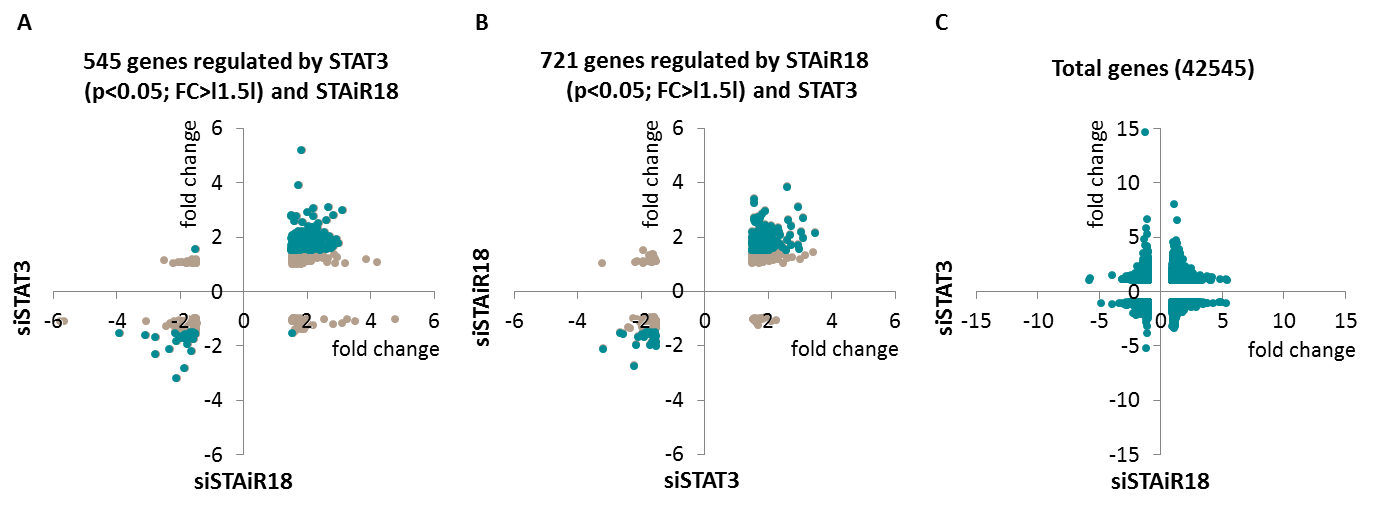
**

**Figure S10: Comparison of genes regulated by the knockdown of STAT3 and STAiR18.** (A) Fold changes (FCs) of 545 genes significantly regulated by STAT3 knockdown with a minimal fold change of 1.5 and a maximum p-value of 0.05 (see Additional Table 5) were plotted against the corresponding FC values obtained after STAiR18 knockdown. Genes showing a minimum fold change of 1.5 in response to STAiR18 knockdown are depicted in blue, all others in beige. (B) Fold changes (FCs) of 721 genes significantly regulated by STAiR18 knockdown with a minimal fold change of 1.5 and a maximum p-value of 0.05 (see Additional Table 6) were plotted against the corresponding FC values obtained after STAT3 knockdown. Genes showing a minimum fold change of 1.5 in response to STAT3 knockdown are depicted in blue, all others in beige. (C) All fold changes (FCs) of genes covered by the array (42,545 protein-coding and non-coding genes) were plotted against each other without applying any cutoff criteria. All FC values obtained by STAT3 and STAiR18 knockdowns were normalized to the negative control siRNA.

## Figure S11: STAT3 protein levels are STAiR18 regulated

**
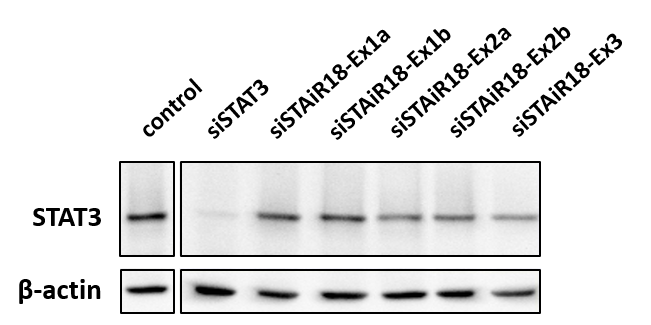
**

**Figure S11: STAT3 protein levels are regulated by STAiR18.** Permanently IL‑6 treated INA‑6 cells were transfected with siRNAs targeting STAiR18 exon 1 (in two positions a+b), exon 2 (in two positions a+b), and exon 3 or transfected with a negative control siRNA. Protein was isolated 48 h after transfection, separated by SDS-PAGE, and detected by immunoblotting (IB) using specific antibodies for STAT3 and β-actin.

## Figure S12: STAiR18 associates with specific RNA (A) and DNA (B) targets.


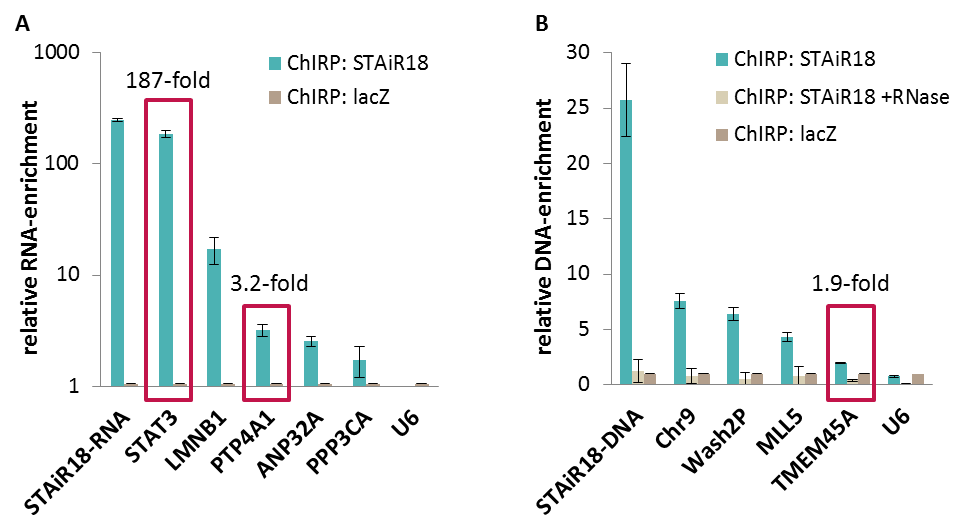


**Figure S12: STAiR18 associates with specific targets.** STAiR18-pulldown and analysis of its binding partners was carried out by ChIRP-RNA- and DNA-seq. LacZ-pulldown served as a negative control. Target genes were validated by qPCR. RNase was added to one STAiR18-RNA-pulldown sample as a control for specific enrichment. RNA (A) and DNA (B) were isolated after pulldown and analyzed by qPCR using specific primers. Primers for U6 were used as a control for unspecific binding. Values were normalized to lacZ and are expressed as mean ± StDev (n=3). PTP4A1 and TMEM45A, which were found here as STAiR18 binding partners on RNA and DNA level, respectively, were also regulated at the transcript levels upon STAiR18 knockdown and hence, like STAT3, are STAiR18 target genes (red boxes).

# Additional tables

## Table S1: (q) PCR primers

**Additional Table 1: Listing of used (q) PCR primers complementary to the Methods section of the manuscript.** The following PCR primers were designed using Primer3 software and provided by Eurofins Scientific, Luxembourg, Luxembourg.

| Primer | Sequence |
| --- | --- |
| STAiR18-Ex1_for (both) | 5'-CGTGCCTGTCTTCAGATCTTC-3' |
| STAiR18-Ex2_rev (both) | 5'-GGGAATCTTTCAGCTGCATT-3' |
| STAiR18-ExK_for | 5'-ACTCATGCCCAAAGTTACGG-3' |
| STAiR18-ExK_rev | 5'-CCGTAACTTTGGGCATGAGT-3' |
| STAiR18_In1_rev | 5'-AGAAACGCAGTGTCCTCACC-3' |
| STAiR18-Ex3_rev | 5ˈ-CACCAGCATCTTTTCCAACC-3ˈ |
| STAiR18-Ex4_for | 5ˈ-TTTTCCCTGCTCTGCTCCTC-3ˈ |
| STAiR18-Ex4_rev | 5ˈ-CCCTGCTCTGCTCCTCTAAA-3ˈ |
| STAiR18-Ex5_rev | 5ˈ-ACCCAGGGAGGATAGCACAT-3ˈ |
| STAiR18-Ex8_rev | 5ˈ-AGTCAAGGAGTCAGGCAGAG-3ˈ |
| MALAT1_for | 5'-CTACTGGGCTGACATTAACT-3' |
| MALAT1_rev | 5'-ACAAAAGCTACCATCAGAAG-3' |
| GAPDH_for | 5'-GTCAGTGGTGGACCTGACCT-3' |
| GAPDH_rev | 5'-AGGGGAGATTCAGTGTGGTG-3' |
| HOTAIR_for | 5ˈ-GGGAGTTCCACAGACCAACA-3ˈ |
| HOTAIR_rev | 5ˈ-TTAGGGACCTGAGGGTCTAAGTC-3ˈ |
| STAiR18-A_for | 5'-CGTAGGAAGGCTTGGGGTTA-3' |
| STAiR18-A_rev | 5'-TTAGCAGAGGGGGTCTCTCA-3' |
| STAiR18-B_for | 5'-AAGGCTTGGCTGGTGGAT-3' |
| STAiR18-B_rev | 5'-ATCTTCCCACGCTACACGTC-3' |
| U6_for | 5'-CTCGCTTCGGCAGCACA-3' |
| U6_rev | 5'-AACGCTTCACGAATTTGCGT-3' |
| STAT3_for | 5'-CTGGCCTTTGGTGTTGAAAT-3' |
| STAT3_rev | 5'-AAGGCACCCACAGAAACAAC-3' |
| STAT3_pomoter_for | 5'-CAGGAGGGAGCTGTATCAGG-3' |
| STAT3_promoter_rev | 5'-AATTCATGAAAGGCCAGCTC-3' |
| STAT3-RNA-BS1_for | 5'-GAAACAGGCTGGGTGTGTTG-3' |
| STAT3-RNA-BS1_rev | 5'-GGGATAAAGATGCTCTGGGGA-3' |
| STAT3-RNA-BS2_for | 5'-CCACGATCGCACCTTTCATT-3' |
| STAT3-RNA-BS2_rev | 5'-TGAGAGACTGAACACACGCA-3' |
| PTP4A1_for | 5ˈ-ccagctcctgtggaagtcac-3ˈ |
| PTP4A1_rev | 5ˈ-ccatcatcaaaaggccaatc-3ˈ |
| TMEM45A_for | 5'-GGAGAACAGCTGGCTAAGGA-3' |
| TMEM45A_rev | 5'- TTCATAGTGTGGGCATCCAA-3' |
| loc101927865_for | 5'-GGGTGAGTCTGATGCAGTGA-3' |
| loc101927865_rev | 5'-TGCTTTGGAATTTGGCATTT-3' |

## Table S2: Stealth siRNAs

**Additional Table 2: Listing of stealth siRNAs used for knockdown complementary to the Methods section of the manuscript.** The following stealth siRNAs were designed using the Block-iT™ RNAi Designer and provided by LIFE Technologies, Carlsbad, USA.

| SiRNA | Sequence |
| --- | --- |
| siSTAT3 (Ex9) | 5'-tttgttgacgggtctgaagttgaga-3' |
| siSTAiR18-Ex1.1 | 5'-tgcctgtcttcagatcttcacagca-3' |
| siSTAiR18-Ex1.2* | 5'-AGTTTCAAATTGACATTCCAGACAA-3' |
| siSTAiR18-ExK | 5'-catgcccaaagttacggaggaccca-3' |
| siSTAiR18-Ex2.1 | 5'-tgtgGactctgaggcctctgcattt-3' |
| siSTAiR18-Ex2.2* | 5'-TGCGGGTGGTCTGCCTGTGATATTT-3' |
| siSTAiR18-Ex3 | 5'-CCAACCTTAATGAACTGTATCCTCA-3' |
| siSTAiR18-Ex8 | 5'-CCACGACTAGGTCACTACTGCTTTA-3' |

*siRNAs indicated with an asterisk were used to exclude possible off target effects and resulted in comparable results

## Table S3: Antibodies

**Additional Table 3: Listing of primary and secondary antibodies used for ímmunoblotting complementary to the Methods section of the manuscript.**

| Primary antibody | Catalog no. | Dilution | Manufacturer |
| --- | --- | --- | --- |
| anti-STAT3 | S21320 | 1:2500 | BD Transduction Laboratories, Franklin Lakes, New Jersey, USA |
| anti-pY705-STAT3 | 9145S | 1:2000 | Cell Signaling |
| anti-SETD2 | STJ25493 | 1:1000 | St. Johns Laboratory |
| anti-β-actin | A5316 | 1:5000 | Sigma-Aldrich |
| Secondary antibody | **Catalog no.** | **Dilution** | **Manufacturer** |
| rabbit-anti-mouse-IgG-HRP | P0260 | 1:5000 | DAKO (Glostrup, Denmark) |
| swine-anti-rabbit-IgG-HRP | P0217 | 1:5000 | (DAKO) |

## Table S4: ChIRP oligonucleotides

**Additional Table 4: Listing of used ChIRP oligonucleotides complementary to the Methods section of the manuscript.** The following 3ˈ-biotinylated DNA oligonucleotides (= BITEG; with a TEG spacer between the oligo and biotin) were designed using [www.singlemoleculefish.com](http://www.singlemoleculefish.com) and provided by Eurofins Scientific, Luxembourg, Luxembourg.

| Oligo | Sequence |
| --- | --- |
| lacZ1 | 5'-ccagctttcatcaacattaaatgtg-3'-BITEG |
| lacZ2 | 5'-ataaagaaactgttacccgtaggta-3'-BITEG |
| lacZ3 | 5'-cgttaaagttgttctgcttcatcag-3'-BITEG |
| lacZ4 | 5'-gccgttttcatcatatttaatcagc-3'-BITEG |
| lacZ5 | 5'-agttcaatcaactgtttaccttgtg-3'-BITEG |
| lacZ6 | 5'-tgataaataaggttttcccctgatg-3'-BITEG |
| STAiR18.1 | 5'-ttctcattggaacgagatgactcat-3'-BITEG |
| STAiR18.2 | 5'-tccattcccaatgatgtacacacga-3'-BITEG |
| STAiR18.3 | 5'-tcaggcaccgcttgtctggaatgtc-3'-BITEG |
| STAiR18.4 | 5'-gaactgtgctgtgaagatctgaaga-3'-BITEG |
| STAiR18.5 | 5'-cctgtttcatctcccagttattcag-3'-BITEG |
| STAiR18.6 | 5'-atattcgatcaagtgtgtcatagag-3'-BITEG |
| STAiR18.7 | 5'-tcagctgcattccggctgtgatcgg-3'-BITEG |
| STAiR18.8 | 5'-ggttggaaccaggccccagggaatc-3'-BITEG |
| STAiR18.9 | 5'-caaatgcagaggcctcagagtccac-3'-BITEG |
| STAiR18.10 | 5'-catgaccaaaatatcacaggcagac-3'-BITEG |
| STAiR18.11 | 5'-cagacaaatgggaaaccgaccagac-3'-BITEG |
| STAiR18.12 | 5'- aatgaaggacaagggattaagacac-3'-BITEG |

## Table S5: Duplication of STAiR18 in the Neandertal and Denisova genomes

The presence of the duplicated locus in the Neandertal and Denisova genomes was investigated using the DNA-sequencing read data displayed in the UCSC genome browser (1, 2, 3). According to the available BAM files, all mapped read positions are 'primary'. The reported coverage of the two STAiR18 copies is nearly identical in all cases (Additional Table 1), and even slightly higher than the coverage of the ENCODE pilot regions. Thus, both loci are clearly present in the Neandertal and Denisova genomes. The sequence divergence between the two loci, 0.0066 ± 0.0008 (estimated as the length-weighted average of sequence identities of blat alignments), is consistent with a timing of the duplication before the divergence of Denisova and Modern Human. Using the same method for estimating the Human/Chimp divergence we obtain 0.0132 ± 0.0010, a value consistent with the published genome-wide divergence of 0.0122 (4). The Human/Denisova divergence is estimated at about 13 % relative to the split of the human and chimpanzee lineages (3). Our estimate places the STAiR18 duplication at 50 ± 10 % of the divergence time between Human and Chimp, which is clearly after the Human/Chimp and long before the Human/Denisova divergence events. Consequently, we do not expect to see the duplication of the STAiR18 locus in any other primate genome.

**Table S5: Comparison of human STAiR18 loci with Neandertal and Denisova genomes.** Coverage of DNA-sequencing data (measured as total number of mapped nucleotides) of STAiR18 loci, the ENCODE pilot regions for comparison, and a close-by 60 k region (chr2:100,000,001-100,060,000) in 6 Neandertal (blue) and 1 Denisova (yellow) specimen identified by their UCSC Hg19 track names. Relative coverage (Rel) compared to the ENCODE regions is shown except for the first three Neandertal samples, where the sequencing was too sparse for accurate estimates of local coverage. If the locus were not duplicated, we would see a relative coverage to 0.5 for both loci.

| Track / region | ENCODE | LINC00152 | Rel | LOC541471 | Rel | 60k | Rel |
| --- | --- | --- | --- | --- | --- | --- | --- |
| Region size | 29,955,196 | 66,061 |  | 66,029 |  | 60,000 |  |
| BamSLFeld1 | 26,049 | 36 | - | 51 | - | 98 | - |
| BamSLMez1 | 601,210 | 2,191 | - | 1,380 | - | 968 | - |
| BamSLSid1253 | 24,878 | 0 | - | 69 | - | 118 | - |
| BamSLVi33dot16 | 15,214,101 | 41,336 | 1.23 | 41,582 | 1.24 | 27,835 | 0.92 |
| BamSLVi33dot25 | 12,893,416 | 34,211 | 1.20 | 30,889 | 1.08 | 24,032 | 0.93 |
| BamSLVi33dot26 | 12,429,640 | 32,920 | 1.20 | 32,776 | 1.20 | 23,315 | 0.94 |
| DhcBamDenisova | 914,207,162 | 2,093,630 | 1.04 | 2,023,039 | 1.00 | 1,918,750 | 1.05 |

(1) Kent WJ, Sugnet CW, Furey TS, Roskin KM, Pringle TH, Zahler AM, et al. The Human Genome Browser at UCSC. Genome Res. 2002;12:996–1006.

(2) Green RE, Krause J, Briggs AW, Maricic T, Stenzel U, Kircher M, et al. A Draft Sequence of the Neandertal Genome. Science. 2010;328:710–23.

(3) Meyer M, Kircher M, Gansauge M, Li H, Racimo F, Mallick S, et al. A High-Coverage Genome Sequence from an Archaic Denisovan Individual. Science. 2012;338:222–27.

(4) Chen F, Li W. Genomic Divergences between Humans and Other Hominoids and the Effective Population Size of the Common Ancestor of Humans and Chimpanzees. Am J Hum Genet. 2001;68:444–56.

## Table S6: Absence of STAiR18 duplication in other primate genomes

A comparison of the human STAiR18 sequences with the genome assemblies of other primates shows that a duplicated STAiR18 region is indeed absent in all genomes with the sole exception of the Gorilla genome assembly gorGor3.1 (Additional Table 2). A closer inspection of this duplication in Gorilla strongly suggests that this is an artifact: first, both assembled loci are riddled with large intervals of non-determined sequence and the actual amount of apparently duplicated sequence is conspicuously short, amounting to only about 15 kb, which is less than a quarter of the supposedly duplicated region. This low value cannot be explained to be the overall coverage and quality of this genome assembly. Furthermore, these duplicated sequence regions are nearly identical (99.6-99.9 % sequence identity, compared to 99.1-99.6 % identity between the two human loci). This small divergence is inconsistent with the hypothesis that the STAiR18 duplication predates the Human/Gorilla divergence: this would imply a sequence divergence at 5-10 times larger (4). Finally, the Gorilla genome assembly has been guided by the human genome (5). In summary, we conclude that the apparent duplication of the STAiR18 locus in GorGor3.1 is an assembly artifact.

**Table S6:** **BLAT results of mapping human STAiR18 loci**

**(Hg19) to primate genomes.**

| UCSC genome | Locus |
| --- | --- |
| Human | chr2:87,754,919-87,821,549  chr2:112,187,112-112,253,383 |
| Chimp | chr2A:111,535,765-111,602,906 |
| Bonobo (GENBANK) | scf1120388622829:912727-847206(-) |
| Gorilla | chr2A:88,894,973-88,951,887  chr2A:109,254,996-109,304,706 |
| Orang Utan | chr2a:19,413,005-19,479,678 |
| Gibbon | chr14:74,165,566-74,232,288 |
| Rhesus | chr13:110,608,519-110,674,072 |
| Baboon | chr13:102,203,286-102,268,840 |
| Squirrel Monkey | JH378124: 404,991-472,534 |

(4) Chen F, Li W. Genomic Divergences between Humans and Other Hominoids and the Effective Population Size of the Common Ancestor of Humans and Chimpanzees. Am J Hum Genet. 2001;68:444–56.

(5) Scally A, Dutheil JY, Hillier LW, Jordan GE, Goodhead I, Herrero J, et al. Insights into hominid evolution from the gorilla genome sequence. Nature. 2012;483(7388):169–75.

## Table S7: Genes regulated by STAT3 knockdown

**Table S7: Genes regulated by the knockdown of STAT3.** Permanently IL-6-stimulated INA-6 cells were transfected with an siRNA targeting STAT3 mRNA and a negative control siRNA. 40 h posttransfection RNA was isolated, DNase-digested, followed by library preparation and expression analysis by microarrays. Regulated genes were identified and mapped to the human genome using GeneSpring software. 545 differentially regulated candidates (fold change of more than 1.5 compared to the controls and a p-value of less than 0.05) are listed. Candidates shown in bold italic letters were chosen for validation by qPCR.

| Gene name | Fold change | p-value | Probe |
| --- | --- | --- | --- |
| *STAT3* | ***-5.79*** | ***0.0002*** | ***A_24_P116805*** |
| SOCS3 | -5.66 | 0.0003 | A_23_P207058 |
| chr14:107,131,060-107,131,119 | 4.76 | 0.0364 | A_33_P3235876 |
| GPR18 | 4.18 | 0.0019 | A_23_P14165 |
| PDZRN4 | -3.90 | 0.0025 | A_23_P105651 |
| S1PR1 | 3.83 | 0.0005 | A_23_P404481 |
| FAM65B | 3.48 | 0.0147 | A_23_P358394 |
| FAM65B | 3.25 | 0.0014 | A_24_P941359 |
| TLR4 | 3.19 | 0.0078 | A_32_P66881 |
| chr13:43,406,188-43,406,247 | 3.11 | 0.0402 | A_19_P00325780 |
| C6orf89 | -3.09 | 0.0068 | A_24_P612446 |
| LOC100509763 | -3.08 | 0.0276 | A_33_P3343967 |
| KLF2 | 3.07 | 0.0014 | A_23_P119196 |
| chr7:142,008,722-142,008,781 | 2.99 | 0.0022 | A_24_P59053 |
| EFCAB5 | 2.93 | 0.0319 | A_33_P3341399 |
| CCL2 | 2.92 | 0.0013 | A_23_P89431 |
| chr22:23,040,847-23,040,906 | 2.88 | 0.0167 | A_33_P3364959 |
| BMPR1A | 2.84 | 0.0014 | A_33_P3219256 |
| *loc101927865* | ***2.84*** | ***0.0268*** | ***A_19_P00321038*** |
| THNSL1 | 2.83 | 0.0364 | A_33_P3331601 |
| LOC100129894 | 2.82 | 0.0094 | A_33_P3416398 |
| chrX:27,850,970-27,851,029 | 2.81 | 0.0304 | A_33_P3366396 |
| chr5:67,093,235-67,093,294 | 2.79 | 0.0378 | A_19_P00316166 |
| chr3:106,096,985-106,097,044 | 2.77 | 0.0494 | A_33_P3255135 |
| FLJ43390 | -2.77 | 0.0124 | A_24_P840042 |
| chr19:52,577,082-52,577,141 | -2.76 | 0.0419 | A_19_P00329538 |
| XLOC_003303 | 2.71 | 0.0393 | A_19_P00317501 |
| chr22:23,090,349-23,090,408 | 2.70 | 0.0028 | A_33_P3304696 |
| HTR3D | 2.69 | 0.0384 | A_24_P14974 |
| KRTAP9-8 | 2.67 | 0.0230 | A_33_P3307457 |
| MOBKL2B | 2.65 | 0.0074 | A_33_P3284883 |
| PRAMEF4 | 2.62 | 0.0290 | A_33_P3319896 |
| chr5:127,405,968-127,406,027 | 2.62 | 0.0369 | A_19_P00329019 |
| chr3:171,506,466-171,506,525 | 2.62 | 0.0233 | A_19_P00319324 |
| VAX1 | 2.60 | 0.0414 | A_33_P3381398 |
| SGK1 | -2.54 | 0.0016 | A_23_P19673 |
| TTTY1 | 2.53 | 0.0401 | A_24_P323131 |
| FLJ25758 | 2.53 | 0.0132 | A_24_P110284 |
| FBLN5 | 2.53 | 0.0032 | A_23_P151805 |
| TTBK1 | 2.53 | 0.0311 | A_33_P3215282 |
| chr3:80,803,970-80,804,029 | 2.49 | 0.0257 | A_19_P00809122 |
| C6orf145 | 2.49 | 0.0188 | A_24_P272290 |
| TSPEAR | 2.48 | 0.0409 | A_32_P7581 |
| C6orf176 | 2.47 | 0.0195 | A_32_P8546 |
| BHLHE40 | -2.46 | 0.0002 | A_24_P268676 |
| ULBP3 | 2.43 | 0.0452 | A_23_P134100 |
| RAB37 | 2.43 | 0.0257 | A_23_P414654 |
| ZNF879 | 2.42 | 0.0216 | A_24_P791669 |
| LOC100509924 | 2.37 | 0.0426 | A_23_P11980 |
| C10orf67 | 2.36 | 0.0392 | A_32_P337442 |
| chr12:5,635,075-5,635,134 | 2.36 | 0.0178 | A_19_P00321608 |
| VSX2 | 2.35 | 0.0435 | A_33_P3244906 |
| L1CAM | 2.34 | 0.0208 | A_24_P207995 |
| DKFZP434H168 | 2.34 | 0.0496 | A_24_P936014 |
| AGTR1 | 2.34 | 0.0323 | A_23_P166616 |
| IL13RA1 | -2.33 | 0.0075 | A_24_P280113 |
| chr8:101,513,196-101,513,255 | -2.32 | 0.0314 | A_19_P00322509 |
| chr2:38,408,930-38,408,989 | 2.32 | 0.0451 | A_19_P00316135 |
| TLR4 | 2.31 | 0.0011 | A_24_P69538 |
| FAM194B | 2.28 | 0.0336 | A_32_P470868 |
| chr9:16,017,052-16,017,111 | 2.26 | 0.0310 | A_19_P00809243 |
| LRRC2 | 2.25 | 0.0165 | A_23_P334798 |
| OR6C4 | 2.25 | 0.0494 | A_33_P3379190 |
| ASS1 | -2.25 | 0.0186 | A_23_P31921 |
| LMF1 | 2.25 | 0.0333 | A_33_P3883985 |
| OLFML3 | -2.23 | 0.0031 | A_24_P11315 |
| C6orf89 | -2.23 | 0.0023 | A_33_P3215028 |
| chr13:114,621,136-114,621,195 | 2.22 | 0.0235 | A_19_P00322160 |
| chr2:8,027,131-8,027,190 | 2.20 | 0.0233 | A_19_P00319044 |
| chr5:124,494,393-124,494,452 | 2.20 | 0.0095 | A_19_P00316884 |
| SIT1 | 2.19 | 0.0042 | A_23_P43369 |
| LOC100131096 | 2.19 | 0.0094 | A_24_P857624 |
| HEG1 | -2.19 | 0.0016 | A_32_P166693 |
| chr2:67,402,661-67,402,720 | 2.19 | 0.0318 | A_33_P3299791 |
| chr22:23,063,581-23,063,640 | 2.17 | 0.0224 | A_33_P3279861 |
| LCE5A | 2.17 | 0.0090 | A_33_P3403018 |
| MT1F | -2.17 | 0.0262 | A_23_P15174 |
| TK2 | 2.17 | 0.0118 | A_33_P3392537 |
| LOC100510454 | 2.17 | 0.0049 | A_33_P3213169 |
| ARHGEF10L | 2.17 | 0.0117 | A_33_P3799936 |
| chr2:238,330,063-238,330,122 | 2.16 | 0.0367 | A_33_P3258627 |
| chr3:18,567,952-18,568,011 | 2.16 | 0.0212 | A_19_P00321549 |
| ITGA6 | -2.16 | 0.0018 | A_23_P210176 |
| chr13:62,902,180-62,902,239 | 2.15 | 0.0325 | A_33_P3215166 |
| SYN3 | -2.15 | 0.0401 | A_23_P80295 |
| ITIH5 | -2.14 | 0.0381 | A_24_P292253 |
| XLOC_000918 | 2.14 | 0.0447 | A_19_P00331853 |
| *PTP4A1* | ***-2.13*** | ***0.0177*** | ***A_19_P00315634*** |
| PHF20L1 | -2.12 | 0.0205 | A_33_P3278916 |
| CTTN | -2.12 | 0.0262 | A_33_P3310780 |
| CCL1 | 2.11 | 0.0063 | A_23_P49759 |
| SIRPG | 2.11 | 0.0086 | A_33_P3343873 |
| chr5:86,263,280-86,263,339 | 2.10 | 0.0152 | A_19_P00317839 |
| PLA2G4D | 2.09 | 0.0208 | A_33_P3361611 |
| PDZD7 | 2.09 | 0.0206 | A_23_P75088 |
| chr11:107,047,965-107,048,024 | -2.08 | 0.0070 | A_24_P178444 |
| HOXA11 | 2.08 | 0.0284 | A_33_P3264528 |
| NUMBL | -2.08 | 0.0287 | A_33_P3225091 |
| HBEGF | -2.07 | 0.0049 | A_24_P140608 |
| LOC128322 | 2.07 | 0.0270 | A_33_P3399713 |
| SLC25A24 | -2.07 | 0.0276 | A_23_P74799 |
| chr5:88,393,882-88,393,941 | 2.07 | 0.0092 | A_19_P00805097 |
| chr7:26,535,442-26,535,501 | 2.07 | 0.0310 | A_19_P00316602 |
| chr12:122,031,364-122,031,423 | 2.06 | 0.0139 | A_19_P00812235 |
| DSC1 | 2.06 | 0.0314 | A_23_P38696 |
| DCLK1 | -2.06 | 0.0061 | A_33_P3302777 |
| CD38 | 2.05 | 0.0099 | A_23_P167328 |
| FLJ37201 | 2.05 | 0.0471 | A_24_P246091 |
| RAB3IP | -2.04 | 0.0422 | A_33_P3322450 |
| TWSG1 | -2.04 | 0.0129 | A_24_P202497 |
| PTPRZ1 | -2.04 | 0.0019 | A_23_P168761 |
| HIVEP3 | 2.04 | 0.0274 | A_33_P3216694 |
| SENP1 | -2.04 | 0.0003 | A_23_P204536 |
| STRA6 | 2.03 | 0.0048 | A_33_P3307500 |
| LOC100128126 | -2.02 | 0.0336 | A_33_P3355408 |
| chr15:31,745,355-31,745,414 | 2.02 | 0.0065 | A_33_P3357232 |
| TRIM29 | 2.02 | 0.0010 | A_23_P203267 |
| LOC100288902 | 2.01 | 0.0375 | A_33_P3391316 |
| FHDC1 | -2.01 | 0.0006 | A_23_P328259 |
| COL8A2 | 2.01 | 0.0025 | A_24_P365975 |
| CALHM2 | 2.00 | 0.0181 | A_33_P3343045 |
| MT1G | -1.99 | 0.0074 | A_33_P3233645 |
| LOC728978 | 1.99 | 0.0496 | A_32_P52153 |
| TLE1 | 1.99 | 0.0414 | A_33_P3217731 |
| chr7:19,959,528-19,959,587 | 1.99 | 0.0115 | A_19_P00321301 |
| KRTAP19-4 | 1.99 | 0.0062 | A_33_P3311258 |
| ANKRD30B | 1.99 | 0.0085 | A_33_P3277659 |
| ZNF236 | -1.98 | 0.0112 | A_23_P55601 |
| BCL6 | -1.98 | 0.0030 | A_23_P57856 |
| CD83 | 1.98 | 0.0027 | A_33_P3310415 |
| NPY1R | 1.97 | 0.0160 | A_23_P69699 |
| CHL1 | 1.97 | 0.0339 | A_23_P212241 |
| chr4:49,292-49,351 | 1.97 | 0.0380 | A_33_P3310567 |
| PALM2 | 1.97 | 0.0250 | A_23_P216579 |
| chr3:53,155,207-53,155,266 | -1.96 | 0.0377 | A_33_P3311001 |
| TRIM34 | 1.96 | 0.0201 | A_23_P124190 |
| MT1H | -1.96 | 0.0231 | A_33_P3368313 |
| HEPHL1 | 1.95 | 0.0418 | A_33_P3215043 |
| SLC7A11 | -1.95 | 0.0018 | A_33_P3242623 |
| PTP4A3 | -1.95 | 0.0003 | A_33_P3315906 |
| CDKN2D | 1.94 | 0.0073 | A_23_P89941 |
| SNN | 1.94 | 0.0275 | A_24_P30923 |
| OR9G1 | 1.94 | 0.0465 | A_33_P3348564 |
| CPEB4 | -1.94 | 0.0115 | A_23_P251937 |
| LOC286059 | 1.93 | 0.0341 | A_33_P3630785 |
| OR8B2 | 1.93 | 0.0095 | A_33_P3253578 |
| THAP2 | -1.93 | 0.0233 | A_24_P350437 |
| PARD6B | -1.93 | 0.0186 | A_32_P205637 |
| LOC643962 | -1.93 | 0.0475 | A_33_P3353757 |
| chr8:16,113,842-16,113,901 | 1.93 | 0.0307 | A_19_P00319993 |
| chr6:22,146,831-22,146,890 | 1.92 | 0.0269 | A_19_P00322855 |
| GPRC5D | 1.91 | 0.0007 | A_23_P105691 |
| SLC17A8 | 1.90 | 0.0114 | A_24_P124647 |
| OR51E1 | 1.90 | 0.0374 | A_33_P3424491 |
| IL24 | 1.90 | 0.0398 | A_33_P3290780 |
| TEPP | 1.90 | 0.0213 | A_33_P3415888 |
| SLC39A12 | -1.89 | 0.0226 | A_23_P24149 |
| ZFYVE28 | -1.89 | 0.0068 | A_32_P104746 |
| OR4A5 | 1.89 | 0.0337 | A_33_P3214412 |
| chr7:30,589,552-30,589,611 | 1.88 | 0.0471 | A_19_P00319177 |
| BCAS4 | 1.88 | 0.0032 | A_24_P143492 |
| chr3:37,224,092-37,224,151 | -1.88 | 0.0377 | A_19_P00801109 |
| chr18:77,514,715-77,514,774 | 1.88 | 0.0471 | A_33_P3423315 |
| HTR5A | 1.87 | 0.0295 | A_23_P42565 |
| LOC400756 | -1.87 | 0.0005 | A_33_P3620087 |
| GUCY2C | 1.87 | 0.0094 | A_23_P76312 |
| GLI2 | 1.87 | 0.0322 | A_23_P209246 |
| ABCA6 | 1.87 | 0.0280 | A_23_P500400 |
| chr14:101,295,525-101,295,584 | 1.87 | 0.0107 | A_33_P3362536 |
| PRSS55 | 1.87 | 0.0490 | A_33_P3336944 |
| AHR | -1.86 | 0.0070 | A_23_P215566 |
| SERPINA10 | 1.86 | 0.0310 | A_23_P128759 |
| PTP4A3 | -1.86 | 0.0026 | A_23_P112774 |
| chr1:116,627,442-116,627,501 | 1.86 | 0.0349 | A_19_P00809224 |
| NBPF11 | -1.86 | 0.0451 | A_32_P149492 |
| APOBR | 1.86 | 0.0092 | A_23_P54770 |
| ARHGEF17 | -1.85 | 0.0030 | A_23_P76015 |
| PAPD5 | -1.85 | 0.0039 | A_33_P3377459 |
| chr6:109,676,155-109,676,214 | -1.85 | 0.0493 | A_33_P3333232 |
| FAM162B | 1.85 | 0.0372 | A_23_P145054 |
| DDI1 | 1.84 | 0.0370 | A_33_P3322484 |
| AHNAK | 1.84 | 0.0374 | A_23_P426636 |
| chr5:1,176,270-1,176,329 | 1.84 | 0.0303 | A_19_P00321159 |
| IL2RB | -1.84 | 0.0129 | A_24_P203000 |
| MPP5 | -1.84 | 0.0028 | A_33_P3248967 |
| CDRT15L2 | 1.84 | 0.0265 | A_33_P3356762 |
| C1orf68 | -1.84 | 0.0232 | A_33_P3285456 |
| HLA-DRB6 | 1.84 | 0.0186 | A_24_P169013 |
| chr5:92,899,080-92,899,139 | 1.84 | 0.0388 | A_19_P00318635 |
| PLEKHG1 | 1.84 | 0.0479 | A_33_P3367471 |
| CES1 | 1.83 | 0.0049 | A_33_P3241269 |
| GPR65 | 1.83 | 0.0355 | A_23_P14564 |
| chr1:111,030,303-111,030,362 | 1.83 | 0.0112 | A_33_P3787645 |
| chr22:22,453,563-22,453,622 | 1.83 | 0.0441 | A_33_P3372266 |
| ARHGAP17 | 1.82 | 0.0065 | A_24_P401739 |
| ZNF345 | -1.82 | 0.0209 | A_33_P3318596 |
| ACPP | -1.82 | 0.0220 | A_24_P37589 |
| ADAMTSL4 | -1.82 | 0.0024 | A_23_P115011 |
| chr2:132,056,255-132,056,314 | 1.82 | 0.0072 | A_33_P3317460 |
| HMGA2 | 1.82 | 0.0204 | A_23_P95930 |
| LOC647946 | -1.81 | 0.0263 | A_32_P72181 |
| DEFB107A | -1.81 | 0.0173 | A_33_P3352772 |
| GOLM1 | -1.81 | 0.0048 | A_33_P3231653 |
| CNTNAP3 | 1.81 | 0.0294 | A_24_P418203 |
| chr7:25,988,357-25,988,416 | -1.81 | 0.0002 | A_19_P00803598 |
| chr5:92,855,226-92,855,285 | 1.81 | 0.0304 | A_19_P00324846 |
| C4orf45 | 1.81 | 0.0484 | A_23_P386964 |
| LOC388942 | 1.80 | 0.0111 | A_33_P3350988 |
| MRVI1 | 1.80 | 0.0356 | A_33_P3311403 |
| IL16 | 1.80 | 0.0010 | A_23_P61057 |
| RAPGEF4 | -1.80 | 0.0035 | A_23_P17192 |
| FIGLA | 1.79 | 0.0181 | A_33_P3315944 |
| ITIH5 | -1.79 | 0.0315 | A_33_P3420380 |
| LOC644841 | 1.79 | 0.0417 | A_33_P3339715 |
| STRA6 | 1.79 | 0.0005 | A_33_P3307495 |
| GAL3ST3 | 1.79 | 0.0461 | A_33_P3275390 |
| TUBB3 | -1.79 | 0.0031 | A_23_P77493 |
| ARHGAP21 | 1.79 | 0.0307 | A_23_P115608 |
| CERK | -1.78 | 0.0027 | A_24_P62237 |
| NRXN3 | 1.78 | 0.0253 | A_33_P3851023 |
| AIM2 | 1.77 | 0.0070 | A_32_P44394 |
| CTH | -1.77 | 0.0005 | A_23_P126103 |
| HBCBP | 1.77 | 0.0055 | A_33_P3385882 |
| RALGPS2 | -1.77 | 0.0066 | A_24_P173746 |
| RASGRP2 | 1.77 | 0.0346 | A_33_P3209962 |
| chr20:30,009,242-30,009,301 | 1.76 | 0.0441 | A_33_P3417602 |
| HSPG2 | 1.76 | 0.0065 | A_33_P3380625 |
| CHSY3 | -1.76 | 0.0404 | A_23_P368886 |
| C1orf110 | 1.76 | 0.0319 | A_33_P3250830 |
| XLOC_002977 | 1.76 | 0.0076 | A_19_P00318544 |
| GLYATL1P4 | 1.76 | 0.0113 | A_23_P24688 |
| C7orf46 | -1.76 | 0.0013 | A_32_P59678 |
| IL13RA1 | -1.76 | 0.0018 | A_24_P288685 |
| TAGAP | -1.76 | 0.0124 | A_23_P339588 |
| OR8D4 | 1.75 | 0.0470 | A_33_P3384202 |
| chr4:150,354,549-150,354,608 | 1.75 | 0.0368 | A_33_P3241949 |
| chr11:70,514,004-70,514,063 | 1.75 | 0.0058 | A_19_P00318525 |
| GSTO2 | -1.74 | 0.0295 | A_23_P202206 |
| chrUn_gl000231:13,571-13,630 | 1.74 | 0.0270 | A_33_P3382949 |
| ZNF738 | 1.74 | 0.0284 | A_33_P3231750 |
| LOC219731 | 1.74 | 0.0382 | A_33_P3680789 |
| TP63 | 1.74 | 0.0205 | A_24_P273756 |
| ZNF184 | -1.74 | 0.0053 | A_23_P156620 |
| chr6:168,081,001-168,081,060 | 1.74 | 0.0179 | A_24_P522786 |
| ABHD3 | -1.74 | 0.0048 | A_23_P305759 |
| LOC647323 | 1.74 | 0.0213 | A_24_P376379 |
| C2orf57 | 1.74 | 0.0355 | A_23_P324419 |
| NEK10 | 1.73 | 0.0114 | A_33_P3422213 |
| FCER2 | 1.73 | 0.0094 | A_23_P164773 |
| ACE2 | 1.73 | 0.0269 | A_23_P252981 |
| PPAPDC1B | -1.73 | 0.0101 | A_23_P322845 |
| TDG | -1.73 | 0.0001 | A_23_P204579 |
| CASK | 1.73 | 0.0459 | A_33_P3244096 |
| HHIP | -1.73 | 0.0018 | A_23_P167129 |
| LOC100131129 | 1.73 | 0.0390 | A_33_P3393456 |
| LOC100133857 | 1.73 | 0.0378 | A_33_P3280360 |
| RAB40B | -1.73 | 0.0073 | A_23_P129801 |
| chr1:79,941,982-79,942,041 | 1.73 | 0.0396 | A_19_P00326169 |
| RN5-8S1 | 1.73 | 0.0410 | A_33_P3399064 |
| LOC100132686 | -1.72 | 0.0354 | A_33_P3212769 |
| MBNL2 | -1.72 | 0.0133 | A_24_P56317 |
| RALGPS2 | -1.72 | 0.0150 | A_33_P3291998 |
| CCR3 | 1.72 | 0.0149 | A_23_P250302 |
| chr11:8,008,836-8,008,895 | 1.72 | 0.0013 | A_33_P3248948 |
| chr5:54,207,289-54,207,348 | 1.71 | 0.0179 | A_19_P00324742 |
| CSNK1G3 | -1.71 | 0.0399 | A_24_P234792 |
| XLOC_006828 | -1.71 | 0.0314 | A_19_P00809335 |
| LOC728573 | 1.71 | 0.0461 | A_33_P3269626 |
| HES7 | -1.71 | 0.0180 | A_33_P3368695 |
| HYDIN | 1.71 | 0.0418 | A_33_P3236137 |
| TRAF3IP3 | 1.70 | 0.0081 | A_33_P3281403 |
| SPP1 | -1.70 | 0.0058 | A_23_P7313 |
| RBMY3AP | 1.70 | 0.0462 | A_33_P3393121 |
| COL14A1 | 1.70 | 0.0496 | A_23_P216361 |
| ARL11 | 1.70 | 0.0155 | A_32_P902957 |
| MXRA7 | -1.70 | 0.0019 | A_33_P3239587 |
| ZNF135 | 1.70 | 0.0230 | A_23_P348227 |
| MARVELD3 | 1.70 | 0.0492 | A_23_P152428 |
| FLJ40039 | -1.70 | 0.0345 | A_33_P3212764 |
| CERS3 | 1.69 | 0.0376 | A_24_P943017 |
| PWWP2A | 1.69 | 0.0101 | A_33_P3275943 |
| LOC280665 | 1.69 | 0.0446 | A_33_P3626360 |
| MSMB | 1.69 | 0.0420 | A_24_P146683 |
| TRPM8 | 1.69 | 0.0282 | A_33_P3317327 |
| chr1:151,683,323-151,683,382 | 1.69 | 0.0225 | A_33_P3255939 |
| LOC339894 | 1.69 | 0.0373 | A_33_P3459365 |
| ATP2B4 | -1.69 | 0.0058 | A_33_P3327200 |
| POU2AF1 | -1.69 | 0.0001 | A_23_P312920 |
| ATP2B4 | -1.68 | 0.0011 | A_24_P405205 |
| TMEM2 | 1.68 | 0.0471 | A_33_P3390713 |
| chrX:64,628,302-64,628,361 | 1.68 | 0.0024 | A_19_P00804020 |
| CCDC120 | -1.68 | 0.0183 | A_23_P73540 |
| chr22:21,583,027-21,583,086 | 1.68 | 0.0122 | A_33_P3383075 |
| PSMG4 | -1.68 | 0.0046 | A_33_P3390960 |
| YPEL2 | 1.68 | 0.0174 | A_24_P787947 |
| LOC728084 | 1.67 | 0.0318 | A_33_P3213665 |
| chr2:112,475,427-112,475,486 | 1.67 | 0.0096 | A_33_P3423210 |
| BTBD8 | 1.67 | 0.0454 | A_24_P564030 |
| TNKS1BP1 | -1.67 | 0.0464 | A_33_P3365732 |
| DIP2C | 1.67 | 0.0339 | A_24_P360722 |
| GOLM1 | -1.67 | 0.0019 | A_23_P146512 |
| chr20:31,108,619-31,108,678 | 1.67 | 0.0147 | A_23_P303548 |
| chr19:37,119,685-37,119,744 | 1.66 | 0.0400 | A_33_P3315724 |
| SPIB | 1.66 | 0.0255 | A_23_P39067 |
| chrX:97,834,009-97,834,068 | 1.66 | 0.0052 | A_19_P00321586 |
| PLS1 | -1.66 | 0.0184 | A_23_P211909 |
| chr17:75,441,984-75,442,043 | 1.66 | 0.0086 | A_33_P3355493 |
| ITGA8 | -1.66 | 0.0202 | A_23_P46781 |
| BCL2A1 | 1.66 | 0.0434 | A_23_P152002 |
| ATG2B | -1.66 | 0.0186 | A_33_P3342160 |
| chr9:476,688-476,747 | 1.66 | 0.0462 | A_19_P00317621 |
| HOXA13 | 1.65 | 0.0384 | A_23_P389281 |
| ZNF584 | 1.65 | 0.0436 | A_33_P3228637 |
| chr7:26,411,639-26,411,698 | 1.65 | 0.0309 | A_33_P3344521 |
| SECISBP2L | -1.65 | 0.0062 | A_24_P82135 |
| chr2:129,357,276-129,357,335 | 1.65 | 0.0260 | A_19_P00315658 |
| OR5M3 | 1.65 | 0.0423 | A_33_P3343506 |
| ISG15 | 1.65 | 0.0023 | A_23_P819 |
| PMP22 | 1.65 | 0.0054 | A_33_P3274930 |
| HHEX | 1.65 | 0.0175 | A_23_P47034 |
| PNPLA7 | 1.65 | 0.0088 | A_33_P3338047 |
| SVEP1 | 1.65 | 0.0498 | A_24_P187774 |
| NUP62CL | -1.65 | 0.0155 | A_23_P33914 |
| LOC148189 | -1.65 | 0.0287 | A_33_P3705884 |
| CDCA7 | 1.65 | 0.0014 | A_23_P251421 |
| TRAF3IP3 | 1.64 | 0.0385 | A_23_P323761 |
| COPA | -1.64 | 0.0371 | A_32_P20454 |
| C11orf16 | 1.64 | 0.0157 | A_23_P13486 |
| MT1E | -1.64 | 0.0395 | A_23_P206724 |
| FBN1 | 1.64 | 0.0229 | A_33_P3348239 |
| KCNJ10 | -1.64 | 0.0036 | A_24_P387875 |
| EIF5 | -1.64 | 0.0080 | A_24_P398810 |
| AP3S1 | -1.64 | 0.0059 | A_33_P3286254 |
| chr2:173,808,267-173,808,326 | -1.64 | 0.0109 | A_33_P3316261 |
| LOC732275 | 1.64 | 0.0337 | A_23_P302302 |
| LOC729911 | 1.64 | 0.0139 | A_33_P3405204 |
| LOC100131096 | 1.64 | 0.0062 | A_32_P19000 |
| chr19:53,367,158-53,367,217 | 1.63 | 0.0070 | A_33_P3323059 |
| ATP8B1 | 1.63 | 0.0314 | A_33_P3415191 |
| GPR148 | 1.63 | 0.0425 | A_33_P3317508 |
| RCN2 | -1.63 | 0.0057 | A_32_P175539 |
| chrX:37,326,609-37,326,668 | 1.63 | 0.0438 | A_19_P00800124 |
| FBXO15 | -1.63 | 0.0353 | A_23_P342709 |
| FCRL5 | -1.63 | 0.0299 | A_33_P3335506 |
| chr2:114,581,392-114,581,451 | 1.63 | 0.0023 | A_19_P00808794 |
| chr19:12,114,654-12,114,713 | 1.63 | 0.0245 | A_19_P00811229 |
| PPME1 | -1.62 | 0.0004 | A_24_P4705 |
| chr17:79,390,391-79,390,450 | 1.62 | 0.0410 | A_33_P3379056 |
| chr1:219,391,957-219,392,016 | -1.62 | 0.0066 | A_19_P00802094 |
| MLF1 | -1.62 | 0.0230 | A_24_P345679 |
| BEST3 | 1.62 | 0.0151 | A_33_P3249888 |
| LOC729444 | 1.62 | 0.0178 | A_33_P3307536 |
| chr18:39,096,115-39,096,174 | 1.62 | 0.0305 | A_19_P00318981 |
| ZNF697 | 1.62 | 0.0268 | A_33_P3240727 |
| C17orf87 | 1.62 | 0.0301 | A_24_P272451 |
| SF3B3 | -1.62 | 0.0102 | A_23_P135914 |
| FAIM3 | 1.62 | 0.0073 | A_23_P138125 |
| CSDA | -1.62 | 0.0068 | A_24_P625382 |
| DEFB116 | 1.61 | 0.0189 | A_33_P3278689 |
| GRIA4 | 1.61 | 0.0212 | A_33_P3497352 |
| chr20:37,055,065-37,055,124 | 1.61 | 0.0338 | A_19_P00318304 |
| C14orf165 | 1.61 | 0.0303 | A_33_P3232032 |
| C9orf95 | -1.61 | 0.0182 | A_23_P32036 |
| chr22:27,189,260-27,189,319 | -1.61 | 0.0245 | A_19_P00322461 |
| IRS1 | -1.61 | 0.0459 | A_24_P802145 |
| SEL1L | -1.61 | 0.0048 | A_24_P219114 |
| DEK | 1.61 | 0.0340 | A_23_P254702 |
| HAVCR1 | 1.61 | 0.0075 | A_23_P347610 |
| DCLK1 | -1.61 | 0.0060 | A_23_P369994 |
| CCL22 | 1.60 | 0.0039 | A_24_P313418 |
| chr3:44,159,204-44,159,263 | -1.60 | 0.0013 | A_19_P00326494 |
| PTH | 1.60 | 0.0264 | A_23_P24835 |
| MYO1B | -1.60 | 0.0244 | A_23_P361049 |
| PIM2 | -1.60 | 0.0021 | A_24_P379104 |
| SH3RF3 | -1.60 | 0.0072 | A_24_P589266 |
| C16orf57 | -1.60 | 0.0052 | A_24_P107941 |
| UHRF1BP1 | -1.60 | 0.0102 | A_33_P3313421 |
| LOC100271722 | 1.60 | 0.0362 | A_33_P3323779 |
| chrX:45,707,376-45,707,435 | 1.59 | 0.0038 | A_19_P00317904 |
| PCDHGB4 | -1.59 | 0.0384 | A_23_P359588 |
| MOBKL2B | 1.59 | 0.0446 | A_23_P146551 |
| chr8:108,569,479-108,569,537 | -1.59 | 0.0411 | A_33_P3345821 |
| SEC24D | -1.59 | 0.0240 | A_23_P159382 |
| TUBAL3 | 1.59 | 0.0158 | A_33_P3332252 |
| JUB | -1.59 | 0.0251 | A_23_P54055 |
| LOC254028 | 1.59 | 0.0146 | A_33_P3362306 |
| SLC12A8 | -1.59 | 0.0347 | A_33_P3298861 |
| FCRL2 | -1.59 | 0.0277 | A_24_P319647 |
| COPZ2 | -1.59 | 0.0329 | A_23_P101093 |
| TIE1 | 1.59 | 0.0193 | A_23_P126416 |
| PDGFRA | -1.59 | 0.0107 | A_23_P300033 |
| chr2:90,260,191-90,260,250 | -1.59 | 0.0168 | A_33_P3281435 |
| AP3S1 | -1.59 | 0.0019 | A_23_P69958 |
| HNRNPA1 | -1.58 | 0.0187 | A_33_P3384260 |
| LOC645591 | 1.58 | 0.0293 | A_32_P394491 |
| EIF2AK3 | -1.58 | 0.0253 | A_23_P135857 |
| chr18:53,772,337-53,772,396 | 1.58 | 0.0005 | A_19_P00318677 |
| OR2AT4 | 1.58 | 0.0234 | A_33_P3365621 |
| IRX3 | 1.58 | 0.0485 | A_23_P152235 |
| chr16:57,807,720-57,807,779 | 1.58 | 0.0047 | A_33_P3333995 |
| XLOC_014192 | -1.58 | 0.0020 | A_19_P00322900 |
| FADS1 | -1.58 | 0.0032 | A_24_P192994 |
| C15orf29 | -1.58 | 0.0452 | A_23_P77286 |
| FAS | -1.57 | 0.0408 | A_33_P3332112 |
| BACE1 | 1.57 | 0.0153 | A_33_P3240941 |
| RNF166 | -1.57 | 0.0065 | A_23_P385500 |
| TLE1 | 1.57 | 0.0025 | A_23_P135239 |
| BCL3 | -1.57 | 0.0153 | A_23_P4662 |
| chr6:94,516,803-94,516,862 | 1.57 | 0.0350 | A_19_P00328034 |
| chrX:108,871,460-108,871,519 | 1.57 | 0.0213 | A_19_P00326531 |
| GNA12 | -1.57 | 0.0064 | A_23_P215265 |
| chrX:73,167,273-73,167,332 | 1.57 | 0.0290 | A_19_P00802989 |
| SLAMF7 | -1.57 | 0.0089 | A_24_P353638 |
| SPN | 1.57 | 0.0006 | A_33_P3404706 |
| IZUMO1 | 1.57 | 0.0201 | A_24_P340247 |
| chr8:107,371,779-107,371,838 | -1.57 | 0.0193 | A_19_P00322418 |
| chr2:132,122,430-132,122,489 | 1.57 | 0.0438 | A_33_P3393088 |
| IL16 | 1.57 | 0.0084 | A_24_P73599 |
| ATP2B4 | -1.57 | 0.0000 | A_23_P11841 |
| CHPF | -1.57 | 0.0222 | A_33_P3262012 |
| KSR1 | 1.57 | 0.0276 | A_23_P207774 |
| ALDH2 | -1.56 | 0.0154 | A_23_P36753 |
| VANGL1 | 1.56 | 0.0042 | A_24_P199655 |
| LYL1 | 1.56 | 0.0004 | A_33_P3215422 |
| chr8:42,009,873-42,009,932 | 1.56 | 0.0154 | A_33_P3273752 |
| DRD5 | 1.56 | 0.0388 | A_24_P862886 |
| WFS1 | -1.56 | 0.0017 | A_23_P121499 |
| EMX2 | 1.56 | 0.0262 | A_23_P44264 |
| KLHL24 | 1.56 | 0.0497 | A_24_P521994 |
| LOC285548 | 1.56 | 0.0019 | A_24_P892494 |
| XBP1 | -1.56 | 0.0008 | A_23_P120845 |
| KDM3A | -1.56 | 0.0140 | A_23_P395075 |
| KRTAP9-9 | 1.56 | 0.0232 | A_23_P311585 |
| CYP4F2 | 1.56 | 0.0133 | A_33_P3359017 |
| ATP1B3 | -1.56 | 0.0009 | A_23_P68007 |
| MAP3K8 | -1.56 | 0.0029 | A_23_P23947 |
| CD69 | 1.56 | 0.0016 | A_33_P3241021 |
| RMND5A | -1.56 | 0.0148 | A_33_P3237699 |
| chr1:226,006,301-226,006,360 | 1.56 | 0.0336 | A_19_P00803678 |
| CSDA | -1.56 | 0.0072 | A_23_P25224 |
| chr11:13,848,066-13,848,125 | 1.56 | 0.0253 | A_33_P3328564 |
| chr9:32,946,029-32,946,088 | -1.56 | 0.0072 | A_19_P00810599 |
| APOF | 1.55 | 0.0139 | A_23_P87491 |
| C1orf168 | 1.55 | 0.0249 | A_32_P182299 |
| LIMS3L | 1.55 | 0.0010 | A_33_P3268310 |
| chr2:30,575,235-30,575,294 | 1.55 | 0.0375 | A_33_P3546033 |
| DNAJB9 | -1.55 | 0.0003 | A_23_P258944 |
| PRC1 | -1.55 | 0.0418 | A_23_P206059 |
| FCRLA | 1.55 | 0.0220 | A_24_P276576 |
| chr19:24,229,020-24,229,079 | 1.55 | 0.0207 | A_19_P00322737 |
| TPPP | 1.55 | 0.0014 | A_33_P3371999 |
| chr11:70,866,098-70,866,157 | 1.55 | 0.0321 | A_19_P00812777 |
| FGF8 | 1.55 | 0.0164 | A_23_P46829 |
| NRK | -1.55 | 0.0119 | A_33_P3275220 |
| chr9:43,608,283-43,608,342 | 1.55 | 0.0274 | A_33_P3366412 |
| SURF4 | -1.55 | 0.0019 | A_33_P3222783 |
| chr9:2,744,749-2,744,808 | 1.55 | 0.0450 | A_19_P00804590 |
| TRIM37 | -1.55 | 0.0226 | A_23_P21230 |
| IRF9 | 1.55 | 0.0062 | A_23_P65442 |
| OSTBETA | 1.55 | 0.0452 | A_23_P436284 |
| VAT1L | 1.54 | 0.0407 | A_23_P400449 |
| CHRM1 | 1.54 | 0.0239 | A_33_P3367860 |
| chr17:21,910,349-21,910,408 | 1.54 | 0.0383 | A_33_P3299125 |
| HSD3B1 | 1.54 | 0.0451 | A_24_P350397 |
| LOC646626 | 1.54 | 0.0050 | A_32_P703 |
| MTIF2 | 1.54 | 0.0235 | A_33_P3357143 |
| chr4:84,771,772-84,771,831 | 1.54 | 0.0007 | A_19_P00322146 |
| TLE1 | 1.54 | 0.0084 | A_33_P3210180 |
| PHGDH | -1.54 | 0.0179 | A_23_P85783 |
| IFI27 | 1.53 | 0.0447 | A_24_P270460 |
| TCP1 | -1.53 | 0.0357 | A_33_P3651282 |
| CXCR4 | 1.53 | 0.0077 | A_23_P102000 |
| chr8:55,306,146-55,306,205 | 1.53 | 0.0093 | A_19_P00800792 |
| PGM3 | -1.53 | 0.0014 | A_23_P19592 |
| chr19:21,665,785-21,665,844 | 1.53 | 0.0468 | A_19_P00317340 |
| ANG | -1.53 | 0.0032 | A_33_P3236177 |
| PAIP2B | -1.53 | 0.0133 | A_24_P860797 |
| SLC47A1 | -1.53 | 0.0003 | A_33_P3403399 |
| PSMD7 | -1.53 | 0.0005 | A_23_P106741 |
| TRAF3IP3 | 1.53 | 0.0119 | A_33_P3421351 |
| HOXC9 | -1.53 | 0.0014 | A_23_P25150 |
| chr7:12,804,857-12,804,916 | 1.53 | 0.0145 | A_19_P00805315 |
| XLOC_002779 | -1.53 | 0.0264 | A_19_P00802417 |
| LAPTM4A | -1.53 | 0.0107 | A_23_P90659 |
| SP110 | 1.52 | 0.0185 | A_23_P120002 |
| chr8:118,762,935-118,762,994 | 1.52 | 0.0046 | A_19_P00807638 |
| chr15:102,467,188-102,467,247 | -1.52 | 0.0476 | A_33_P3321263 |
| RGS20 | -1.52 | 0.0272 | A_23_P73097 |
| CD8B | -1.52 | 0.0351 | A_23_P159335 |
| chr11:61,129,035-61,129,094 | 1.52 | 0.0078 | A_33_P3284039 |
| TAS2R7 | 1.52 | 0.0009 | A_23_P36624 |
| FUT8 | -1.52 | 0.0199 | A_23_P313632 |
| CNTLN | 1.52 | 0.0280 | A_33_P3296240 |
| SERPINI1 | -1.52 | 0.0304 | A_23_P166929 |
| TBX5 | 1.52 | 0.0149 | A_24_P30557 |
| NLRP5 | 1.52 | 0.0290 | A_23_P4962 |
| chr15:69,383,452-69,383,511 | 1.52 | 0.0436 | A_19_P00322669 |
| chr14:96,523,149-96,523,208 | 1.52 | 0.0451 | A_19_P00327941 |
| TMEM38B | -1.52 | 0.0002 | A_23_P60259 |
| ST8SIA4 | 1.52 | 0.0468 | A_23_P435601 |
| GRAMD1B | 1.52 | 0.0177 | A_24_P58620 |
| ANTXR2 | 1.52 | 0.0253 | A_23_P170733 |
| LOC286114 | 1.52 | 0.0442 | A_33_P3857091 |
| ASNS | -1.52 | 0.0140 | A_23_P145694 |
| TMEM146 | 1.52 | 0.0082 | A_33_P3335262 |
| chr15:67,223,877-67,223,936 | 1.52 | 0.0468 | A_33_P3323887 |
| FCRL2 | -1.52 | 0.0376 | A_23_P160751 |
| C1QL1 | 1.52 | 0.0302 | A_23_P77993 |
| PRDM1 | -1.52 | 0.0261 | A_33_P3342081 |
| TMED5 | -1.51 | 0.0047 | A_24_P54178 |
| SIRT5 | 1.51 | 0.0374 | A_32_P151366 |
| STAM | -1.51 | 0.0020 | A_33_P3287840 |
| TAGAP | -1.51 | 0.0009 | A_24_P354724 |
| ITGA6 | -1.51 | 0.0490 | A_33_P3231447 |
| STAT3 | -1.51 | 0.0064 | A_23_P100795 |
| PDK1 | -1.51 | 0.0026 | A_24_P37441 |
| CD8B | -1.51 | 0.0401 | A_23_P357881 |
| SURF4 | -1.51 | 0.0475 | A_24_P89971 |
| CCDC34 | -1.51 | 0.0242 | A_23_P203773 |
| SRPK2 | -1.51 | 0.0037 | A_23_P406438 |
| chr8:49,627,717-49,627,776 | -1.51 | 0.0399 | A_19_P00322059 |
| PAQR9 | -1.51 | 0.0236 | A_32_P196193 |
| MT1L | -1.51 | 0.0482 | A_23_P427703 |
| BTG2 | -1.51 | 0.0139 | A_23_P62901 |
| MMRN2 | 1.51 | 0.0178 | A_33_P3278211 |
| chr4:185,742,395-185,742,454 | 1.51 | 0.0276 | A_33_P3400023 |
| RAB9B | 1.51 | 0.0458 | A_23_P406448 |
| chr3:104,571,791-104,571,850 | 1.51 | 0.0478 | A_19_P00322205 |
| BCL11A | 1.51 | 0.0108 | A_33_P3249589 |
| RUNX1 | -1.51 | 0.0033 | A_33_P3211804 |
| C6orf89 | -1.51 | 0.0111 | A_33_P3215023 |
| PRAMEF19 | 1.51 | 0.0133 | A_33_P3232778 |
| C1orf138 | -1.50 | 0.0036 | A_33_P3275412 |
| CCL14 | 1.50 | 0.0211 | A_23_P218369 |
| SSR1 | -1.50 | 0.0037 | A_33_P3396692 |
| chr1:178,715,500-178,715,559 | -1.50 | 0.0167 | A_23_P207940 |
| ZSCAN5B | 1.50 | 0.0366 | A_33_P3406651 |
| MAOB | 1.50 | 0.0369 | A_23_P85015 |
| XLOC_005539 | -1.50 | 0.0247 | A_19_P00318255 |
| CHAC1 | -1.50 | 0.0009 | A_33_P3376965 |
| CEACAM3 | 1.50 | 0.0412 | A_33_P3301331 |
| LBP | 1.50 | 0.0383 | A_23_P143178 |
| chr1:149,287,151-149,287,210 | 1.50 | 0.0112 | A_33_P3281728 |
| ST6GAL1 | -1.50 | 0.0103 | A_24_P388528 |
| chr9:21,567,075-21,567,134 | 1.50 | 0.0107 | A_19_P00330641 |

## Table S8: Genes regulated by STAiR18 knockdown

**Table S8: Genes regulated by the knockdown of STAiR18.** Permanently IL-6-stimulated INA-6 cells were transfected with an siRNA targeting STAiR18 exon 1 and a negative control siRNA. 40 h posttransfection RNA was isolated, DNase-digested, followed by library preparation and expression analysis by microarrays. Regulated genes were identified and mapped to the human genome using GeneSpring software. 721 differentially regulated candidates (fold change of more than 1.5 compared to the controls and a p-value of less than 0.05) are listed. Candidates shown in bold italic letters were chosen for validation by qPCR.

| Gene name | Fold change | p-value | Probe |
| --- | --- | --- | --- |
| SLC5A9 | 3.50 | 0.0167 | A_24_P242581 |
| RGL1 | 3.42 | 0.0152 | A_33_P3240532 |
| LOC100131673 | -3.20 | 0.0005 | A_33_P3380076 |
| *PTP4A1* | ***-3.19*** | ***0.0273*** | ***A_19_P00315634*** |
| RBM20 | 3.17 | 0.0339 | A_24_P453497 |
| HTR3D | 3.10 | 0.0317 | A_24_P14974 |
| chr17:39,784,998-39,785,057 | 3.10 | 0.0110 | A_33_P3213522 |
| chr2:67,402,661-67,402,720 | 3.06 | 0.0028 | A_33_P3299791 |
| PTPN21 | 3.01 | 0.0251 | A_33_P3235990 |
| chr3:95,373,431-95,373,490 | 2.98 | 0.0075 | A_33_P3771741 |
| chr13:43,406,188-43,406,247 | 2.97 | 0.0130 | A_19_P00325780 |
| chr1:166,445,171-166,445,230 | 2.96 | 0.0356 | A_19_P00808280 |
| chr7:105,564,368-105,564,427 | 2.78 | 0.0179 | A_19_P00322776 |
| chr22:21,583,027-21,583,086 | 2.75 | 0.0056 | A_33_P3383075 |
| chr2:216,617,438-216,617,497 | 2.72 | 0.0406 | A_19_P00801749 |
| NCRNA00336 | 2.72 | 0.0244 | A_33_P3227736 |
| chr14:22,111,732-22,111,791 | -2.66 | 0.0077 | A_33_P3247890 |
| PRAMEF4 | 2.63 | 0.0144 | A_33_P3319896 |
| C6orf195 | 2.61 | 0.0030 | A_23_P308042 |
| C9orf47 | 2.60 | 0.0180 | A_33_P3248638 |
| MPP2 | 2.60 | 0.0418 | A_33_P3360611 |
| XLOC_001824 | 2.59 | 0.0044 | A_19_P00321039 |
| PIRT | 2.57 | 0.0351 | A_33_P3246663 |
| LOC285370 | 2.55 | 0.0435 | A_33_P3798268 |
| chr10:131,980,948-131,981,007 | -2.55 | 0.0469 | A_19_P00810261 |
| ZNF703 | 2.54 | 0.0233 | A_33_P3385775 |
| chr5:82,202,272-82,202,331 | 2.48 | 0.0354 | A_19_P00810737 |
| TRIM50 | 2.47 | 0.0072 | A_33_P3293748 |
| chr11:89,721,863-89,721,922 | 2.43 | 0.0143 | A_33_P3408162 |
| chr16:69,069,620-69,069,679 | -2.40 | 0.0047 | A_33_P3604069 |
| FLJ37201 | 2.40 | 0.0138 | A_24_P246091 |
| DKFZp686L13185 | 2.38 | 0.0292 | A_33_P3886737 |
| LCE5A | 2.35 | 0.0321 | A_33_P3403018 |
| LOC284950 | 2.33 | 0.0296 | A_33_P3791552 |
| chr6:155,163,249-155,163,308 | -2.33 | 0.0476 | A_19_P00322455 |
| chr21:10,215,446-10,215,505 | 2.33 | 0.0215 | A_33_P3417472 |
| chr2:38,408,930-38,408,989 | 2.33 | 0.0459 | A_19_P00316135 |
| C1orf146 | 2.29 | 0.0469 | A_33_P3361023 |
| MAGEB18 | 2.28 | 0.0072 | A_23_P370054 |
| LYPD6 | 2.25 | 0.0025 | A_33_P3359047 |
| SHISA9 | 2.25 | 0.0189 | A_33_P3280729 |
| PCDHGC3 | 2.25 | 0.0083 | A_23_P218937 |
| chr8:23,744,170-23,744,229 | 2.24 | 0.0437 | A_33_P3292158 |
| chr3:128,563,028-128,563,087 | 2.24 | 0.0185 | A_33_P3250974 |
| MPP2 | -2.23 | 0.0010 | A_23_P394259 |
| chr5:294,315-294,374 | 2.22 | 0.0055 | A_33_P3328543 |
| chr1:116,627,442-116,627,501 | 2.21 | 0.0301 | A_19_P00809224 |
| *TMEM45A* | ***-2.20*** | ***0.0043*** | ***A_33_P3344831*** |
| SESTD1 | -2.19 | 0.0186 | A_33_P3378047 |
| CD93 | 2.19 | 0.0040 | A_32_P56001 |
| DOCK6 | 2.19 | 0.0471 | A_23_P67299 |
| chr10:80,608,065-80,608,124 | 2.19 | 0.0081 | A_19_P00811486 |
| MRAP2 | 2.18 | 0.0437 | A_23_P357207 |
| IL16 | 2.16 | 0.0311 | A_24_P299423 |
| NID1 | 2.16 | 0.0052 | A_33_P3281191 |
| LRRC14B | 2.16 | 0.0400 | A_33_P3253538 |
| SPRY2 | 2.15 | 0.0216 | A_23_P128698 |
| CXorf57 | -2.15 | 0.0405 | A_23_P96369 |
| CHL1 | 2.15 | 0.0251 | A_23_P212241 |
| TOB2P1 | 2.14 | 0.0455 | A_33_P3348011 |
| chr20:42,987,796-42,987,855 | 2.13 | 0.0066 | A_33_P3808435 |
| chr3:162,917,666-162,917,725 | 2.13 | 0.0170 | A_19_P00317648 |
| C17orf87 | 2.13 | 0.0117 | A_24_P272451 |
| NPBWR2 | 2.12 | 0.0343 | A_33_P3269791 |
| BMPER | 2.12 | 0.0165 | A_24_P942904 |
| FRMD3 | 2.12 | 0.0475 | A_33_P3406072 |
| P2RY1 | 2.12 | 0.0185 | A_33_P3417150 |
| CNTNAP3 | 2.11 | 0.0460 | A_24_P418203 |
| DAOA | 2.11 | 0.0011 | A_24_P155617 |
| LOC100130627 | 2.11 | 0.0010 | A_33_P3378534 |
| chrUn_gl000214:51,115-51,174 | -2.11 | 0.0264 | A_19_P00800061 |
| SERPINB11 | 2.11 | 0.0051 | A_33_P3233040 |
| LOC339975 | -2.10 | 0.0328 | A_33_P3723580 |
| KIAA1671 | -2.09 | 0.0459 | A_33_P3415062 |
| chr11:32,459,967-32,460,026 | 2.09 | 0.0211 | A_33_P3248997 |
| ASB9 | 2.08 | 0.0035 | A_23_P125643 |
| chr6:139,015,570-139,015,629 | 2.08 | 0.0217 | A_19_P00320229 |
| OR51E2 | 2.07 | 0.0495 | A_24_P235756 |
| LOC100128420 | 2.07 | 0.0059 | A_32_P77102 |
| C1QTNF9 | 2.06 | 0.0232 | A_33_P3357002 |
| OR56A5 | 2.06 | 0.0080 | A_33_P3210798 |
| chr4:84,771,772-84,771,831 | 2.05 | 0.0032 | A_19_P00322146 |
| LRRC32 | 2.05 | 0.0194 | A_24_P389916 |
| chr15:62,538,649-62,538,708 | 2.04 | 0.0261 | A_33_P3249736 |
| FLJ26245 | 2.03 | 0.0293 | A_33_P3255314 |
| LOC100132919 | 2.03 | 0.0366 | A_33_P3234482 |
| C11orf70 | 2.03 | 0.0127 | A_33_P3333156 |
| chr19:38,314,006-38,314,065 | 2.02 | 0.0334 | A_33_P3347020 |
| chr2:105,481,865-105,481,924 | 2.02 | 0.0036 | A_33_P3274372 |
| CYP26C1 | 2.01 | 0.0266 | A_33_P3264672 |
| EEF1A2 | 2.01 | 0.0375 | A_33_P3229953 |
| LOC440386 | 2.01 | 0.0297 | A_33_P3270271 |
| chr3:63,099,298-63,099,357 | 2.01 | 0.0036 | A_19_P00322164 |
| GPR27 | 2.01 | 0.0251 | A_23_P385835 |
| IL33 | -2.01 | 0.0275 | A_23_P31945 |
| chr6:105,171,394-105,171,453 | 2.00 | 0.0082 | A_19_P00808063 |
| GDEP | -2.00 | 0.0313 | A_33_P3320643 |
| chr4:81,311,765-81,311,824 | 2.00 | 0.0216 | A_33_P3218258 |
| LOC644841 | 1.99 | 0.0103 | A_33_P3339720 |
| ZNF549 | 1.98 | 0.0233 | A_23_P399146 |
| chr14:44,913,950-44,914,009 | 1.98 | 0.0130 | A_33_P3252316 |
| chr5:758,827-758,886 | 1.98 | 0.0347 | A_19_P00803675 |
| SRRM4 | 1.98 | 0.0004 | A_33_P3393200 |
| chr14:95,426,672-95,426,731 | 1.97 | 0.0047 | A_33_P3239190 |
| NBPF7 | 1.97 | 0.0174 | A_33_P3279167 |
| MMP20 | 1.97 | 0.0061 | A_23_P47292 |
| ANGPTL5 | 1.97 | 0.0024 | A_23_P64161 |
| LOC100508383 | -1.97 | 0.0398 | A_33_P3234550 |
| ANO6 | -1.97 | 0.0374 | A_33_P3286804 |
| DUOXA2 | 1.96 | 0.0231 | A_33_P3250443 |
| chr10:101,572,696-101,572,755 | -1.96 | 0.0193 | A_33_P3365644 |
| EPG5 | -1.96 | 0.0262 | A_33_P3261505 |
| XLOC_005849 | 1.95 | 0.0183 | A_19_P00803039 |
| chr12:52,229,894-52,229,953 | -1.95 | 0.0417 | A_19_P00325394 |
| RNF222 | 1.95 | 0.0417 | A_33_P3318449 |
| chr2:168,270,855-168,270,880 | 1.95 | 0.0061 | A_33_P3212432 |
| C11orf86 | 1.95 | 0.0358 | A_32_P524904 |
| TMPRSS12 | -1.94 | 0.0156 | A_32_P44534 |
| TBX5 | 1.94 | 0.0092 | A_24_P30557 |
| chr10:49,944,098-49,944,157 | 1.94 | 0.0372 | A_33_P3395289 |
| chr2:65,623,070-65,623,129 | -1.93 | 0.0263 | A_33_P3215367 |
| chr12:122,031,364-122,031,423 | 1.93 | 0.0453 | A_19_P00812235 |
| LPPR5 | 1.93 | 0.0031 | A_33_P3303319 |
| chr2:86,899,105-86,899,164 | 1.93 | 0.0330 | A_19_P00812239 |
| PLEKHH2 | -1.93 | 0.0140 | A_33_P3315715 |
| NCRNA00239 | -1.92 | 0.0355 | A_32_P176911 |
| KIF3A | -1.92 | 0.0246 | A_33_P3382217 |
| AGTR1 | 1.92 | 0.0247 | A_23_P166616 |
| TLE1 | -1.92 | 0.0346 | A_33_P3379775 |
| EFCAB5 | 1.92 | 0.0463 | A_33_P3341399 |
| FAM198A | 1.92 | 0.0393 | A_23_P69154 |
| chr7:116,211,206-116,211,265 | 1.91 | 0.0202 | A_19_P00318415 |
| chr14:107,259,430-107,259,489 | 1.91 | 0.0289 | A_23_P343900 |
| LOC100129148 | -1.91 | 0.0462 | A_33_P3331021 |
| ADAM18 | 1.91 | 0.0461 | A_23_P72527 |
| SOX13 | 1.91 | 0.0366 | A_23_P85703 |
| chr19:56,774,853-56,774,912 | -1.91 | 0.0175 | A_19_P00318354 |
| chr14:20,228,888-20,228,947 | 1.91 | 0.0258 | A_33_P3250223 |
| XLOC_005397 | 1.91 | 0.0129 | A_19_P00319072 |
| LOC727915 | 1.90 | 0.0461 | A_33_P3237257 |
| PRSS55 | 1.90 | 0.0197 | A_33_P3336944 |
| IFIT2 | 1.90 | 0.0486 | A_24_P304071 |
| PSPC1 | -1.90 | 0.0124 | A_33_P3232173 |
| GPR31 | -1.90 | 0.0178 | A_33_P3375358 |
| LOC285375 | 1.90 | 0.0316 | A_33_P3792370 |
| MGC40069 | 1.90 | 0.0045 | A_33_P3715843 |
| chr18:23,979,510-23,979,569 | 1.89 | 0.0330 | A_19_P00810499 |
| chr18:20,479,106-20,479,165 | 1.89 | 0.0130 | A_19_P00806215 |
| ETV1 | 1.88 | 0.0241 | A_33_P3357949 |
| CALD1 | 1.88 | 0.0455 | A_24_P921366 |
| MCTP1 | 1.88 | 0.0395 | A_24_P212481 |
| PCDHB10 | 1.88 | 0.0027 | A_23_P7397 |
| GNA14 | 1.88 | 0.0031 | A_24_P415150 |
| DHH | 1.88 | 0.0141 | A_33_P3338740 |
| DOK7 | 1.88 | 0.0228 | A_23_P398854 |
| AP1S2 | -1.88 | 0.0365 | A_33_P3397507 |
| SPRYD7 | 1.88 | 0.0220 | A_23_P368718 |
| PPAPDC1A | -1.88 | 0.0340 | A_24_P810290 |
| LOC100131208 | 1.87 | 0.0443 | A_33_P3398578 |
| ATP4B | 1.87 | 0.0106 | A_23_P32308 |
| C6orf118 | 1.87 | 0.0033 | A_32_P85106 |
| KBTBD10 | 1.87 | 0.0111 | A_23_P17190 |
| KCNK2 | 1.87 | 0.0090 | A_33_P3213822 |
| chr7:116,211,028-116,211,087 | 1.87 | 0.0044 | A_19_P00320387 |
| PNMAL1 | 1.87 | 0.0105 | A_24_P296808 |
| ALPK2 | 1.87 | 0.0020 | A_23_P15876 |
| NHLH1 | 1.87 | 0.0438 | A_24_P336474 |
| C10orf112 | -1.87 | 0.0097 | A_33_P3292646 |
| MYO3A | 1.86 | 0.0091 | A_33_P3351622 |
| C14orf182 | 1.86 | 0.0264 | A_33_P3355703 |
| FLJ13197 | 1.86 | 0.0082 | A_23_P30163 |
| OLFM3 | 1.86 | 0.0056 | A_33_P3791263 |
| PRKG1 | 1.86 | 0.0184 | A_33_P3351087 |
| WNK2 | 1.86 | 0.0273 | A_24_P942600 |
| C14orf166B | 1.86 | 0.0416 | A_33_P3471466 |
| AHNAK | 1.86 | 0.0356 | A_24_P943393 |
| DSCR6 | -1.86 | 0.0073 | A_33_P3254956 |
| ZNF713 | 1.85 | 0.0072 | A_33_P3409710 |
| MPPED1 | 1.85 | 0.0074 | A_33_P3263459 |
| chr20:30,009,242-30,009,301 | 1.85 | 0.0344 | A_33_P3417602 |
| C8orf45 | -1.85 | 0.0377 | A_33_P3405384 |
| KCNMA1 | 1.85 | 0.0237 | A_23_P60727 |
| C3orf46 | 1.85 | 0.0418 | A_32_P375062 |
| GCM2 | -1.85 | 0.0004 | A_23_P59285 |
| DACT1 | 1.84 | 0.0454 | A_23_P65518 |
| C11orf44 | 1.84 | 0.0222 | A_33_P3268863 |
| GIMAP5 | 1.84 | 0.0144 | A_23_P42588 |
| chr19:12,114,654-12,114,713 | 1.84 | 0.0409 | A_19_P00811229 |
| chr1:86,095,316-86,095,375 | 1.84 | 0.0042 | A_19_P00807717 |
| ETV7 | 1.84 | 0.0120 | A_23_P42353 |
| chr5:95,657,879-95,657,938 | 1.84 | 0.0004 | A_19_P00802335 |
| chr6:22,083,815-22,083,874 | 1.84 | 0.0363 | A_19_P00318271 |
| EBF3 | 1.83 | 0.0157 | A_33_P3386067 |
| chr18:2,508,887-2,508,946 | 1.83 | 0.0381 | A_19_P00807801 |
| LOC100129894 | 1.83 | 0.0004 | A_33_P3416398 |
| MS4A8B | 1.83 | 0.0008 | A_23_P139146 |
| SLC7A2 | 1.83 | 0.0264 | A_33_P3316539 |
| CCDC108 | 1.83 | 0.0001 | A_33_P3261586 |
| MGC16075 | 1.83 | 0.0252 | A_24_P120734 |
| LOC100133612 | 1.83 | 0.0076 | A_33_P3349036 |
| chr2:104,240,202-104,240,261 | 1.83 | 0.0276 | A_33_P3393617 |
| GPC5 | 1.83 | 0.0289 | A_33_P3368855 |
| chr3:156,478,566-156,478,625 | 1.83 | 0.0304 | A_19_P00315662 |
| LOC100128346 | 1.82 | 0.0011 | A_33_P3333058 |
| LOC100189589 | 1.82 | 0.0003 | A_24_P772436 |
| chr7:12,581,456-12,581,515 | -1.82 | 0.0060 | A_33_P3335458 |
| PKD1L2 | 1.82 | 0.0060 | A_23_P129332 |
| TMSB15A | 1.81 | 0.0496 | A_23_P137173 |
| C15orf52 | 1.81 | 0.0478 | A_23_P163467 |
| ARSD | 1.81 | 0.0008 | A_23_P357760 |
| LSAMP-AS1 | 1.81 | 0.0154 | A_33_P3324114 |
| GPR153 | 1.81 | 0.0370 | A_33_P3302586 |
| TRIM9 | 1.81 | 0.0100 | A_24_P321709 |
| chr7:106,416,157-106,416,216 | 1.81 | 0.0314 | A_19_P00813327 |
| LOC647107 | 1.81 | 0.0033 | A_33_P3659992 |
| chrX:138,266,102-138,266,161 | 1.81 | 0.0063 | A_19_P00813591 |
| TNRC18 | 1.81 | 0.0388 | A_23_P377434 |
| chr8:16,113,842-16,113,901 | 1.80 | 0.0173 | A_19_P00319993 |
| GUCY2F | 1.80 | 0.0404 | A_23_P360302 |
| XKR7 | 1.80 | 0.0221 | A_33_P3304948 |
| OR6C4 | 1.80 | 0.0429 | A_33_P3379190 |
| FABP2 | 1.80 | 0.0285 | A_23_P391711 |
| chr8:130,363,993-130,364,052 | 1.80 | 0.0220 | A_33_P3451508 |
| chr13:44,033,524-44,033,583 | 1.80 | 0.0094 | A_33_P3305920 |
| OR51B6 | 1.80 | 0.0221 | A_33_P3310154 |
| chr2:129,357,671-129,357,730 | 1.79 | 0.0250 | A_19_P00315656 |
| C10orf118 | -1.79 | 0.0205 | A_24_P68031 |
| chr3:177,286,880-177,286,939 | 1.79 | 0.0487 | A_19_P00317883 |
| chr6:86,445,508-86,445,567 | 1.79 | 0.0296 | A_33_P3288047 |
| chr3:13,787,925-13,787,984 | -1.79 | 0.0177 | A_19_P00317431 |
| GSTTP1 | 1.79 | 0.0054 | A_33_P3213265 |
| chr1:152,784,449-152,784,508 | 1.79 | 0.0068 | A_33_P3415047 |
| TSPAN19 | 1.79 | 0.0391 | A_23_P2322 |
| C5AR1 | 1.79 | 0.0389 | A_23_P153562 |
| MICALL2 | 1.79 | 0.0473 | A_24_P303524 |
| chr19:6,412,290-6,412,349 | 1.79 | 0.0095 | A_33_P3219230 |
| OR8H3 | 1.79 | 0.0145 | A_33_P3300858 |
| chrX:110,812,609-110,812,668 | 1.78 | 0.0182 | A_19_P00321176 |
| FRY | 1.78 | 0.0204 | A_33_P3223495 |
| UBTFL1 | 1.78 | 0.0007 | A_33_P3303982 |
| GIMAP6 | 1.78 | 0.0298 | A_23_P145631 |
| chr6:147,183,034-147,183,093 | 1.78 | 0.0428 | A_19_P00319196 |
| GPR137C | 1.78 | 0.0249 | A_33_P3237110 |
| chr6_ssto_hap7:571,469-571,528 | 1.78 | 0.0018 | A_33_P3343350 |
| MERTK | 1.78 | 0.0111 | A_33_P3402091 |
| TCHHL1 | 1.78 | 0.0017 | A_33_P3424122 |
| chr10:988,620-988,679 | 1.78 | 0.0198 | A_32_P120183 |
| chr8:20,311,243-20,311,302 | 1.78 | 0.0204 | A_19_P00808641 |
| SCN1B | 1.78 | 0.0379 | A_23_P79015 |
| OR5B3 | 1.78 | 0.0147 | A_33_P3352263 |
| KIR2DS3 | 1.77 | 0.0494 | A_33_P3311073 |
| MED12L | 1.77 | 0.0001 | A_32_P701268 |
| DSG3 | 1.77 | 0.0348 | A_33_P3348747 |
| chr17:68,855,011-68,855,070 | -1.77 | 0.0314 | A_33_P3414494 |
| FRMPD4 | 1.77 | 0.0097 | A_24_P940275 |
| XLOC_006756 | 1.77 | 0.0126 | A_19_P00317087 |
| chr3:170,410,462-170,410,521 | 1.77 | 0.0069 | A_19_P00804611 |
| CLCN4 | -1.77 | 0.0085 | A_33_P3415097 |
| chr15:25,259,618-25,259,677 | -1.77 | 0.0041 | A_19_P00807732 |
| LOC100128529 | 1.77 | 0.0015 | A_33_P3423595 |
| CERS3 | 1.76 | 0.0383 | A_24_P943017 |
| chr14:86,481,136-86,481,195 | 1.76 | 0.0458 | A_33_P3251906 |
| TRAPPC2 | -1.76 | 0.0099 | A_33_P3366824 |
| SPPL2B | 1.76 | 0.0137 | A_33_P3389678 |
| chr8:108,569,479-108,569,537 | -1.76 | 0.0494 | A_33_P3345821 |
| chr14:106,621,927-106,621,986 | 1.76 | 0.0241 | A_33_P3331178 |
| DLC1 | 1.76 | 0.0164 | A_24_P940115 |
| chr16:2,389,233-2,389,292 | 1.76 | 0.0317 | A_33_P3295932 |
| CRAT | -1.76 | 0.0004 | A_33_P3415843 |
| ALCAM | 1.76 | 0.0059 | A_33_P3713357 |
| chr9:109,366,892-109,366,951 | 1.75 | 0.0254 | A_19_P00315900 |
| KLRD1 | 1.75 | 0.0072 | A_23_P204208 |
| TPTE2P3 | 1.75 | 0.0326 | A_33_P3223182 |
| chr12:2,851,960-2,852,019 | 1.75 | 0.0138 | A_33_P3243268 |
| DHFRL1 | -1.75 | 0.0191 | A_24_P186065 |
| TDH | 1.75 | 0.0084 | A_24_P334845 |
| CHIT1 | 1.75 | 0.0093 | A_23_P126278 |
| LOC221122 | 1.75 | 0.0015 | A_33_P3347887 |
| chr2:96,190,712-96,190,771 | 1.74 | 0.0274 | A_33_P3357227 |
| PLEKHA5 | 1.74 | 0.0345 | A_23_P218068 |
| LMF1 | 1.74 | 0.0166 | A_33_P3883985 |
| ACSM2A | 1.74 | 0.0289 | A_33_P3265129 |
| PKN2 | -1.74 | 0.0255 | A_33_P3242829 |
| TMEM213 | 1.74 | 0.0220 | A_24_P706953 |
| chr14:107,095,171-107,095,230 | -1.74 | 0.0383 | A_24_P813550 |
| KLHL24 | 1.74 | 0.0463 | A_24_P521994 |
| chr6:107,218,600-107,218,659 | 1.74 | 0.0156 | A_19_P00316480 |
| NRP2 | 1.74 | 0.0254 | A_33_P3297415 |
| LIPJ | 1.74 | 0.0218 | A_33_P3215550 |
| BAAT | 1.73 | 0.0365 | A_33_P3249224 |
| C20orf166 | 1.73 | 0.0288 | A_23_P316637 |
| chr19:21,740,046-21,740,105 | 1.73 | 0.0046 | A_19_P00801693 |
| RAB5B | -1.73 | 0.0173 | A_23_P410998 |
| chr14:86,473,705-86,473,764 | 1.73 | 0.0273 | A_19_P00810935 |
| ITGA1 | 1.73 | 0.0008 | A_33_P3353791 |
| LOC100130894 | 1.73 | 0.0061 | A_33_P3355921 |
| ZNF518B | -1.72 | 0.0362 | A_32_P144421 |
| OR3A1 | 1.72 | 0.0476 | A_23_P50031 |
| CCL18 | 1.72 | 0.0357 | A_23_P55270 |
| FAM200B | 1.72 | 0.0433 | A_33_P3411145 |
| chr15:33,442,032-33,442,091 | 1.72 | 0.0326 | A_33_P3379881 |
| CTSL2 | -1.72 | 0.0104 | A_23_P146456 |
| MALL | 1.72 | 0.0170 | A_24_P80204 |
| ZAK | 1.72 | 0.0025 | A_23_P366394 |
| HRH4 | 1.72 | 0.0097 | A_23_P386310 |
| AKD1 | 1.72 | 0.0468 | A_33_P3250979 |
| OR1A2 | 1.72 | 0.0121 | A_23_P130281 |
| NOX3 | 1.71 | 0.0412 | A_23_P82099 |
| HTR5A | 1.71 | 0.0278 | A_23_P42565 |
| CHRNA1 | 1.71 | 0.0035 | A_23_P90888 |
| RAB37 | 1.71 | 0.0007 | A_23_P414654 |
| ZNF236 | -1.71 | 0.0110 | A_23_P55601 |
| NME5 | 1.71 | 0.0465 | A_23_P156402 |
| SLC19A3 | 1.71 | 0.0005 | A_24_P182892 |
| CNTLN | 1.71 | 0.0134 | A_33_P3296240 |
| NR2F2 | 1.71 | 0.0322 | A_23_P88589 |
| PIP5K1P1 | 1.71 | 0.0028 | A_33_P3884610 |
| GPR88 | 1.71 | 0.0323 | A_33_P3380457 |
| LOC399939 | 1.71 | 0.0031 | A_33_P3374970 |
| chr22:32,758,840-32,758,899 | 1.71 | 0.0010 | A_33_P3328274 |
| UNC13A | 1.71 | 0.0206 | A_24_P472007 |
| chr7:105,517,644-105,517,703 | 1.71 | 0.0023 | A_19_P00811717 |
| IRX5 | 1.71 | 0.0279 | A_24_P48057 |
| SELP | 1.71 | 0.0008 | A_23_P137697 |
| chr2:61,158,515-61,158,574 | 1.70 | 0.0201 | A_19_P00315755 |
| chr7:79,100,247-79,100,306 | 1.70 | 0.0476 | A_19_P00320041 |
| AUTS2 | 1.70 | 0.0368 | A_33_P3330209 |
| SHISA2 | 1.70 | 0.0075 | A_32_P55241 |
| PRRX1 | 1.70 | 0.0009 | A_33_P3263890 |
| PXDN | 1.70 | 0.0151 | A_33_P3238166 |
| NTRK2 | 1.70 | 0.0168 | A_33_P3322804 |
| S100B | 1.70 | 0.0016 | A_23_P143526 |
| TTC16 | 1.70 | 0.0278 | A_23_P375419 |
| chr21:27,592,136-27,592,195 | 1.70 | 0.0098 | A_33_P3221223 |
| BTN2A1 | -1.70 | 0.0052 | A_23_P501634 |
| chr18:37,115,461-37,115,520 | 1.70 | 0.0385 | A_19_P00322051 |
| C7orf16 | 1.70 | 0.0366 | A_23_P145724 |
| PDE1C | 1.70 | 0.0241 | A_33_P3331853 |
| RBMY3AP | 1.69 | 0.0265 | A_33_P3393121 |
| RGS22 | 1.69 | 0.0488 | A_32_P125771 |
| PLEK | 1.69 | 0.0014 | A_23_P209678 |
| LOC286370 | 1.69 | 0.0019 | A_33_P3738498 |
| EHHADH | 1.69 | 0.0296 | A_24_P123119 |
| MYLK4 | 1.69 | 0.0455 | A_33_P3252939 |
| chr10:97,114,707-97,114,766 | 1.69 | 0.0051 | A_33_P3316440 |
| LOC641365 | 1.69 | 0.0014 | A_33_P3801085 |
| chr5:92,831,130-92,831,189 | 1.69 | 0.0210 | A_19_P00330734 |
| LOC286149 | 1.69 | 0.0252 | A_33_P3562810 |
| LOC727924 | 1.69 | 0.0390 | A_33_P3414027 |
| LOC286299 | 1.69 | 0.0331 | A_33_P3866448 |
| chr8:134,377,998-134,378,057 | 1.69 | 0.0334 | A_19_P00318036 |
| SASH1 | 1.68 | 0.0042 | A_33_P3209283 |
| BCL10 | 1.68 | 0.0013 | A_33_P3392447 |
| LOC727710 | 1.68 | 0.0083 | A_33_P3872115 |
| C12orf68 | -1.68 | 0.0366 | A_23_P344194 |
| UGT2A3 | 1.68 | 0.0327 | A_24_P334378 |
| chr21:19,299,091-19,299,150 | 1.68 | 0.0030 | A_19_P00328226 |
| CSMD2 | 1.68 | 0.0310 | A_24_P263284 |
| LPPR4 | 1.68 | 0.0272 | A_32_P148538 |
| SLC17A8 | 1.68 | 0.0173 | A_24_P124647 |
| chr5:102,001,097-102,001,156 | -1.68 | 0.0104 | A_19_P00808517 |
| chr1:205,525,515-205,525,567 | 1.68 | 0.0298 | A_33_P3578399 |
| chr12:123,616,671-123,616,730 | 1.68 | 0.0050 | A_19_P00805631 |
| RBPJL | 1.68 | 0.0174 | A_33_P3398609 |
| SYT4 | 1.68 | 0.0023 | A_23_P208030 |
| TMEM146 | 1.68 | 0.0142 | A_23_P340822 |
| C14orf82 | -1.68 | 0.0383 | A_33_P3296772 |
| KCNA1 | 1.67 | 0.0166 | A_24_P498652 |
| chr2:102,034,066-102,034,125 | 1.67 | 0.0485 | A_19_P00318769 |
| chr7:142,119,994-142,120,053 | 1.67 | 0.0118 | A_33_P3257428 |
| MCHR1 | 1.67 | 0.0499 | A_23_P211543 |
| NBLA00301 | 1.67 | 0.0436 | A_33_P3270514 |
| PRL | 1.67 | 0.0380 | A_32_P65616 |
| chr9:471,526-471,585 | 1.67 | 0.0107 | A_19_P00322170 |
| chr10:125,017,843-125,017,902 | 1.67 | 0.0032 | A_19_P00805565 |
| OR8B2 | 1.67 | 0.0248 | A_33_P3253578 |
| chr15:31,745,355-31,745,414 | 1.67 | 0.0414 | A_33_P3357232 |
| chr22:42,898,502-42,898,561 | -1.67 | 0.0449 | A_24_P365679 |
| USP25 | -1.67 | 0.0243 | A_24_P139208 |
| chr2:145,342,761-145,342,820 | 1.66 | 0.0312 | A_19_P00808648 |
| HYDIN | 1.66 | 0.0110 | A_33_P3300232 |
| ZNF709 | 1.66 | 0.0045 | A_24_P365322 |
| SPSB1 | 1.66 | 0.0405 | A_24_P96961 |
| chr7:78,806,467-78,806,526 | 1.66 | 0.0441 | A_33_P3338379 |
| SIRPG | 1.66 | 0.0089 | A_33_P3343873 |
| OR13C5 | 1.66 | 0.0459 | A_33_P3244215 |
| - | 1.66 | 0.0031 | A_33_P3364358 |
| chr2:165,516,847-165,516,906 | -1.66 | 0.0215 | A_19_P00327030 |
| chr11:124,029,515-124,029,574 | -1.66 | 0.0129 | A_23_P104865 |
| LEUTX | 1.66 | 0.0338 | A_24_P796321 |
| OR5J2 | 1.66 | 0.0078 | A_33_P3242064 |
| SCUBE2 | 1.66 | 0.0024 | A_23_P105144 |
| chr12:48,161,040-48,161,099 | 1.66 | 0.0312 | A_19_P00331192 |
| SPANXN5 | 1.66 | 0.0135 | A_33_P3358363 |
| LOC553137 | 1.66 | 0.0022 | A_33_P3275973 |
| SLC46A3 | 1.65 | 0.0320 | A_23_P205074 |
| F13A1 | 1.65 | 0.0394 | A_33_P3416097 |
| LOC286059 | 1.65 | 0.0370 | A_33_P3630785 |
| BDNF | 1.65 | 0.0367 | A_23_P127891 |
| FLJ46257 | 1.65 | 0.0223 | A_33_P3280080 |
| NUDT10 | 1.65 | 0.0238 | A_24_P400044 |
| F2RL1 | 1.65 | 0.0050 | A_33_P3232945 |
| MYBPC1 | 1.65 | 0.0479 | A_33_P3278053 |
| C9orf171 | -1.65 | 0.0225 | A_33_P3354847 |
| NLRP10 | 1.65 | 0.0412 | A_23_P403488 |
| chr9:476,688-476,747 | 1.65 | 0.0119 | A_19_P00317621 |
| AMPD3 | 1.65 | 0.0141 | A_24_P304154 |
| MMP24 | -1.65 | 0.0370 | A_33_P3398331 |
| LOC389300 | 1.65 | 0.0256 | A_33_P3298930 |
| LOC441204 | 1.65 | 0.0426 | A_33_P3318530 |
| DNASE2B | 1.65 | 0.0075 | A_23_P126677 |
| chr2:157,101,400-157,101,459 | 1.65 | 0.0057 | A_19_P00813533 |
| LOC100131000 | -1.65 | 0.0361 | A_33_P3245484 |
| GAB3 | -1.65 | 0.0179 | A_33_P3251369 |
| SFRP2 | 1.65 | 0.0417 | A_23_P81103 |
| TAF7 | -1.64 | 0.0056 | A_23_P81248 |
| PTPRQ | 1.64 | 0.0280 | A_33_P3309471 |
| FRMD3 | 1.64 | 0.0187 | A_33_P3363425 |
| ZNF165 | -1.64 | 0.0292 | A_23_P93269 |
| RFPL2 | 1.64 | 0.0043 | A_23_P254434 |
| CYP4V2 | 1.64 | 0.0371 | A_24_P945228 |
| LOC100507084 | 1.64 | 0.0330 | A_33_P3331916 |
| ARHGEF10L | 1.64 | 0.0061 | A_33_P3799936 |
| AHR | -1.64 | 0.0432 | A_23_P215566 |
| TTC25 | 1.63 | 0.0309 | A_23_P73150 |
| KIAA0776 | -1.63 | 0.0210 | A_23_P30956 |
| RPS6KA2 | 1.63 | 0.0488 | A_33_P3399943 |
| chr6:525,463-525,522 | 1.63 | 0.0005 | A_33_P3268853 |
| USP43 | -1.63 | 0.0264 | A_24_P310009 |
| DSEL | 1.63 | 0.0394 | A_24_P365180 |
| FAM81B | 1.63 | 0.0484 | A_23_P360354 |
| LOC100506464 | 1.63 | 0.0399 | A_33_P3256470 |
| chr6:107,165,355-107,165,414 | 1.63 | 0.0386 | A_19_P00321390 |
| OSTBETA | 1.63 | 0.0344 | A_23_P436284 |
| C1orf61 | 1.63 | 0.0110 | A_32_P138032 |
| PRKCA | 1.63 | 0.0371 | A_24_P916496 |
| STAC | 1.63 | 0.0004 | A_24_P234415 |
| CD28 | 1.63 | 0.0366 | A_23_P91095 |
| chr1:229,387,104-229,387,163 | 1.63 | 0.0443 | A_33_P3226142 |
| LRRC7 | 1.63 | 0.0455 | A_33_P3307840 |
| GJB7 | 1.63 | 0.0237 | A_33_P3273777 |
| HMGA2 | 1.63 | 0.0129 | A_23_P95930 |
| CTBP2 | 1.63 | 0.0166 | A_23_P63897 |
| NKX2-1 | 1.63 | 0.0500 | A_24_P61490 |
| chr6:141,167,677-141,167,736 | 1.63 | 0.0246 | A_19_P00806783 |
| LOC286063 | 1.63 | 0.0190 | A_33_P3773374 |
| SH3PXD2B | -1.63 | 0.0038 | A_32_P24585 |
| LOC100507605 | 1.63 | 0.0318 | A_33_P3648553 |
| FAM129C | 1.63 | 0.0163 | A_23_P345799 |
| PLSCR2 | 1.62 | 0.0214 | A_23_P80508 |
| chr1:111,030,303-111,030,362 | 1.62 | 0.0403 | A_33_P3787645 |
| chr6:72,034,813-72,034,872 | -1.62 | 0.0381 | A_19_P00326525 |
| chr3:171,506,466-171,506,525 | 1.62 | 0.0354 | A_19_P00319324 |
| chr6_cox_hap2:1,222,405-1,222,464 | 1.62 | 0.0497 | A_19_P00316376 |
| chr14:61,530,207-61,530,266 | 1.62 | 0.0386 | A_19_P00812729 |
| chr13:74,161,960-74,162,019 | 1.62 | 0.0202 | A_33_P3335157 |
| chr11:86,621,003-86,621,062 | 1.62 | 0.0262 | A_19_P00803777 |
| chr14:22,180,943-22,181,002 | 1.62 | 0.0218 | A_33_P3321085 |
| chrX:113,010,762-113,010,821 | -1.62 | 0.0223 | A_19_P00324769 |
| LAPTM4B | 1.62 | 0.0264 | A_24_P414999 |
| PER3 | 1.62 | 0.0363 | A_24_P291231 |
| chr10:47,680,137-47,680,196 | 1.62 | 0.0010 | A_33_P3334803 |
| chr12:31,201,937-31,201,986 | -1.62 | 0.0085 | A_33_P3385912 |
| chr21:26,429,973-26,430,032 | 1.62 | 0.0253 | A_33_P3574391 |
| chr3:109,529,579-109,529,638 | 1.62 | 0.0167 | A_19_P00801247 |
| LOC100506241 | 1.62 | 0.0373 | A_33_P3364235 |
| chr3:119,829,634-119,829,693 | 1.62 | 0.0151 | A_19_P00328940 |
| SNX22 | -1.62 | 0.0306 | A_33_P3212172 |
| PDYN | 1.61 | 0.0234 | A_24_P279870 |
| C2orf27A | 1.61 | 0.0285 | A_32_P45974 |
| HRASLS2 | -1.61 | 0.0079 | A_23_P105012 |
| LOC401134 | 1.61 | 0.0298 | A_33_P3433258 |
| LPAR4 | 1.61 | 0.0394 | A_23_P33868 |
| PHF21B | 1.61 | 0.0224 | A_24_P113572 |
| chrX:134,525,774-134,525,833 | 1.61 | 0.0013 | A_33_P3407999 |
| MASP1 | 1.61 | 0.0358 | A_24_P41850 |
| chr16:1,482,854-1,482,913 | 1.61 | 0.0089 | A_33_P3315748 |
| TBC1D30 | 1.61 | 0.0382 | A_32_P206050 |
| FGF20 | 1.61 | 0.0286 | A_33_P3400248 |
| chr6:21,884,211-21,884,270 | 1.61 | 0.0249 | A_19_P00319377 |
| FLJ37638 | 1.61 | 0.0244 | A_33_P3434239 |
| LOC441601 | 1.61 | 0.0468 | A_32_P174164 |
| chr6_mcf_hap5:4,023,052-4,023,111 | 1.61 | 0.0393 | A_33_P3376518 |
| RGS3 | -1.61 | 0.0356 | A_24_P377775 |
| ERG | 1.61 | 0.0005 | A_23_P301414 |
| chr14:32,395,067-32,395,126 | 1.60 | 0.0302 | A_19_P00322507 |
| NR4A3 | 1.60 | 0.0332 | A_23_P306867 |
| chrX:138,266,286-138,266,345 | 1.60 | 0.0444 | A_19_P00805364 |
| SLC2A13 | -1.60 | 0.0060 | A_33_P3354514 |
| CHIC1 | -1.60 | 0.0444 | A_32_P25737 |
| MS4A14 | -1.60 | 0.0062 | A_33_P3290577 |
| chr2:151,618,957-151,619,016 | 1.60 | 0.0430 | A_19_P00805907 |
| chr1:158,778,215-158,778,274 | 1.60 | 0.0370 | A_33_P3358844 |
| GPR56 | 1.60 | 0.0245 | A_23_P206280 |
| PDGFC | 1.60 | 0.0154 | A_23_P58396 |
| IFNA8 | 1.60 | 0.0407 | A_23_P146539 |
| LOC283484 | 1.60 | 0.0198 | A_33_P3679941 |
| C5orf47 | 1.60 | 0.0425 | A_24_P410721 |
| TNKS1BP1 | -1.60 | 0.0361 | A_33_P3365732 |
| chr12:46,915,476-46,915,535 | 1.60 | 0.0430 | A_19_P00317077 |
| CDH4 | 1.60 | 0.0184 | A_23_P17593 |
| ZNF818P | 1.60 | 0.0284 | A_33_P3217594 |
| SPOCK1 | 1.60 | 0.0314 | A_24_P354689 |
| THAP10 | 1.60 | 0.0041 | A_23_P106391 |
| ZNF180 | -1.60 | 0.0262 | A_23_P89921 |
| EVC | 1.60 | 0.0227 | A_24_P60441 |
| chrX:148,138,377-148,138,436 | 1.60 | 0.0164 | A_33_P3404685 |
| SHANK2 | 1.60 | 0.0156 | A_33_P3374010 |
| GAD1 | 1.60 | 0.0388 | A_33_P3365142 |
| C22orf33 | 1.59 | 0.0076 | A_23_P353149 |
| TRIM29 | 1.59 | 0.0288 | A_23_P203267 |
| FLJ31715 | 1.59 | 0.0259 | A_33_P3777165 |
| ZSCAN5B | 1.59 | 0.0323 | A_33_P3406651 |
| IGFBP6 | 1.59 | 0.0144 | A_23_P139912 |
| ABCG4 | 1.59 | 0.0152 | A_24_P355626 |
| chr13:44,813,543-44,813,602 | 1.59 | 0.0318 | A_19_P00813008 |
| LOC440346 | -1.59 | 0.0398 | A_33_P3865403 |
| chr1:220,373,839-220,373,898 | 1.59 | 0.0287 | A_33_P3613605 |
| KIR2DL2 | 1.59 | 0.0045 | A_33_P3313920 |
| OR2T11 | 1.58 | 0.0120 | A_33_P3230886 |
| chr6_qbl_hap6:4,330,986-4,331,045 | 1.58 | 0.0259 | A_19_P00321882 |
| PROS1 | -1.58 | 0.0497 | A_33_P3212555 |
| KCNJ2 | 1.58 | 0.0242 | A_23_P329261 |
| TP53I11 | 1.58 | 0.0147 | A_24_P160969 |
| CSTT | 1.58 | 0.0333 | A_24_P194670 |
| KLRC3 | -1.58 | 0.0106 | A_23_P128281 |
| MSH4 | 1.58 | 0.0374 | A_23_P74068 |
| SMPX | 1.58 | 0.0114 | A_23_P253542 |
| PLXNA4 | 1.58 | 0.0201 | A_33_P3279959 |
| SLC17A6 | 1.58 | 0.0200 | A_23_P24294 |
| DSC1 | 1.58 | 0.0391 | A_23_P38696 |
| KCNK18 | 1.58 | 0.0137 | A_23_P115932 |
| MCHR2 | 1.58 | 0.0111 | A_23_P156784 |
| CA5B | -1.58 | 0.0485 | A_33_P3316052 |
| chr18:74,322,863-74,322,922 | 1.58 | 0.0081 | A_33_P3355946 |
| AKR1C4 | 1.58 | 0.0251 | A_23_P149926 |
| CAMP | 1.57 | 0.0448 | A_23_P253791 |
| C6 | 1.57 | 0.0229 | A_23_P92928 |
| LOC100131822 | 1.57 | 0.0192 | A_33_P3241244 |
| PTH2R | 1.57 | 0.0318 | A_23_P380901 |
| VIT | 1.57 | 0.0121 | A_33_P3300262 |
| KRTAP9-8 | 1.57 | 0.0132 | A_33_P3307457 |
| chrX:145,904,232-145,904,291 | 1.57 | 0.0007 | A_33_P3366739 |
| MICALCL | 1.57 | 0.0281 | A_23_P2041 |
| FGFBP3 | -1.57 | 0.0485 | A_24_P201381 |
| BTNL9 | -1.57 | 0.0026 | A_33_P3312466 |
| LOC401433 | -1.57 | 0.0443 | A_33_P3831099 |
| DNAH7 | 1.57 | 0.0289 | A_23_P33583 |
| chr6:136,391,367-136,391,426 | 1.57 | 0.0343 | A_33_P3378962 |
| chr17:75,846,787-75,846,846 | -1.57 | 0.0093 | A_33_P3294730 |
| PAG1 | 1.57 | 0.0400 | A_32_P61684 |
| METTL21C | 1.57 | 0.0134 | A_24_P212074 |
| ZPBP2 | 1.57 | 0.0146 | A_23_P77852 |
| OSBPL2 | 1.57 | 0.0115 | A_33_P3381827 |
| KCNB1 | 1.57 | 0.0419 | A_24_P31627 |
| chr6:21,668,795-21,668,854 | 1.56 | 0.0458 | A_19_P00316960 |
| chr8:29,444,627-29,444,686 | 1.56 | 0.0043 | A_19_P00318663 |
| FLJ42709 | 1.56 | 0.0386 | A_24_P51080 |
| chr18:67,669,766-67,669,825 | -1.56 | 0.0349 | A_19_P00326844 |
| chr18:33,198,301-33,198,360 | 1.56 | 0.0484 | A_19_P00319402 |
| chr19:57,815,923-57,815,982 | 1.56 | 0.0135 | A_19_P00810972 |
| LMLN | -1.56 | 0.0325 | A_23_P170959 |
| chr4:109,621,972-109,622,031 | 1.56 | 0.0069 | A_19_P00810897 |
| APCDD1L | 1.56 | 0.0269 | A_32_P300427 |
| LPAR3 | 1.56 | 0.0019 | A_23_P436048 |
| KRTAP1-1 | 1.56 | 0.0154 | A_23_P141624 |
| LOC401442 | 1.56 | 0.0358 | A_33_P3342797 |
| BEX1 | 1.56 | 0.0377 | A_23_P159952 |
| CDH20 | 1.56 | 0.0497 | A_23_P55586 |
| LOC286002 | 1.56 | 0.0460 | A_33_P3223417 |
| RSPO4 | 1.56 | 0.0276 | A_33_P3251552 |
| DEPDC5 | -1.56 | 0.0192 | A_33_P3409665 |
| GALC | -1.56 | 0.0495 | A_23_P25964 |
| TNFAIP3 | -1.56 | 0.0414 | A_24_P157926 |
| chr17:8,870,308-8,870,367 | 1.56 | 0.0302 | A_33_P3389802 |
| MBD2 | -1.56 | 0.0308 | A_24_P119201 |
| VAX1 | 1.56 | 0.0288 | A_33_P3381398 |
| CD207 | 1.56 | 0.0253 | A_23_P39790 |
| CCR3 | 1.56 | 0.0034 | A_23_P250302 |
| chr13:109,883,991-109,884,050 | 1.56 | 0.0296 | A_19_P00806436 |
| chr8:91,686,471-91,686,530 | 1.56 | 0.0275 | A_19_P00811156 |
| C1orf110 | 1.56 | 0.0070 | A_24_P6370 |
| chr12:97,919,103-97,919,162 | 1.56 | 0.0458 | A_19_P00323672 |
| chr5:153,476,694-153,476,753 | 1.56 | 0.0018 | A_19_P00808611 |
| LOC646851 | 1.56 | 0.0405 | A_33_P3825317 |
| ANKRD30B | 1.56 | 0.0286 | A_33_P3277659 |
| GUCY2C | 1.55 | 0.0332 | A_23_P76312 |
| GLB1 | -1.55 | 0.0318 | A_23_P61531 |
| SDC2 | 1.55 | 0.0261 | A_33_P3371219 |
| DSC3 | 1.55 | 0.0413 | A_33_P3266898 |
| CDC5L | 1.55 | 0.0413 | A_32_P55804 |
| FREM1 | -1.55 | 0.0295 | A_23_P43337 |
| DPY19L2P1 | 1.55 | 0.0432 | A_33_P3235816 |
| chr19:20,999,241-20,999,300 | -1.55 | 0.0486 | A_19_P00805588 |
| chr18:29,267,215-29,267,274 | 1.55 | 0.0215 | A_33_P3218013 |
| C9orf150 | 1.55 | 0.0294 | A_33_P3375145 |
| SLC4A4 | 1.55 | 0.0153 | A_32_P358887 |
| WHAMMP3 | 1.55 | 0.0174 | A_33_P3317558 |
| MARK1 | 1.55 | 0.0322 | A_24_P179585 |
| PCDHA10 | 1.55 | 0.0489 | A_33_P3281299 |
| KRT84 | 1.55 | 0.0275 | A_33_P3273524 |
| chr18:56,660,972-56,661,031 | 1.55 | 0.0387 | A_19_P00326332 |
| LOC100133857 | 1.55 | 0.0044 | A_33_P3280360 |
| ZNF100 | 1.55 | 0.0440 | A_32_P48244 |
| chr7:148,393,589-148,393,648 | 1.55 | 0.0248 | A_33_P3210358 |
| chr22:20,714,986-20,715,045 | 1.55 | 0.0182 | A_33_P3389926 |
| SASH1 | 1.55 | 0.0184 | A_33_P3209279 |
| chr18:22,567,444-22,567,503 | -1.55 | 0.0500 | A_19_P00315917 |
| XLOC_002241 | 1.55 | 0.0331 | A_19_P00810259 |
| ZIC1 | 1.55 | 0.0247 | A_32_P86067 |
| HRH3 | 1.55 | 0.0010 | A_23_P412990 |
| AATK | 1.55 | 0.0101 | A_23_P10559 |
| GC | 1.55 | 0.0239 | A_23_P167349 |
| chr10:124,585,905-124,585,964 | 1.55 | 0.0493 | A_33_P3327381 |
| chr4:167,017,587-167,017,646 | 1.55 | 0.0392 | A_33_P3242104 |
| TRO | 1.55 | 0.0450 | A_33_P3340014 |
| PLEKHD1 | 1.55 | 0.0469 | A_33_P3353767 |
| chr10:23,462,122-23,462,181 | 1.55 | 0.0424 | A_33_P3312342 |
| DLL1 | 1.55 | 0.0353 | A_23_P167920 |
| chr6:3,978,834-3,978,894 | 1.54 | 0.0248 | A_24_P273043 |
| TMEM130 | 1.54 | 0.0429 | A_23_P349966 |
| CAPS2 | 1.54 | 0.0241 | A_32_P209208 |
| NEK5 | 1.54 | 0.0260 | A_33_P3250521 |
| chr16:75,728,328-75,728,387 | 1.54 | 0.0496 | A_33_P3209185 |
| LUZP2 | 1.54 | 0.0254 | A_24_P649282 |
| DEFB114 | 1.54 | 0.0182 | A_33_P3354201 |
| WDFY4 | 1.54 | 0.0344 | A_32_P321996 |
| chr10:119,345,606-119,345,665 | 1.54 | 0.0254 | A_19_P00324107 |
| chr9:133,624,713-133,624,746 | 1.54 | 0.0270 | A_33_P3299972 |
| LYST | -1.54 | 0.0016 | A_23_P354074 |
| SLITRK3 | 1.54 | 0.0472 | A_23_P18362 |
| LOC100510591 | 1.54 | 0.0191 | A_33_P3286694 |
| OR2A14 | 1.54 | 0.0221 | A_23_P59783 |
| FCAR | 1.54 | 0.0123 | A_24_P348265 |
| OR4D1 | 1.54 | 0.0289 | A_33_P3420605 |
| PRR9 | 1.54 | 0.0319 | A_33_P3210146 |
| GABRA4 | 1.54 | 0.0441 | A_32_P204137 |
| C7orf58 | -1.54 | 0.0401 | A_24_P187799 |
| chr3:177,955,772-177,955,831 | 1.54 | 0.0331 | A_19_P00812006 |
| TDRD6 | 1.54 | 0.0277 | A_33_P3232960 |
| PLEKHA1 | 1.54 | 0.0047 | A_24_P269814 |
| chr10:88,689,283-88,689,342 | 1.54 | 0.0290 | A_19_P00809528 |
| SVEP1 | 1.53 | 0.0359 | A_24_P187774 |
| AR | 1.53 | 0.0124 | A_23_P113111 |
| ADD2 | 1.53 | 0.0286 | A_33_P3241786 |
| CACNB2 | 1.53 | 0.0303 | A_33_P3285275 |
| WIF1 | 1.53 | 0.0208 | A_32_P216520 |
| TCTEX1D1 | 1.53 | 0.0438 | A_32_P160045 |
| TRPM6 | 1.53 | 0.0402 | A_24_P26792 |
| chr5:134,474,940-134,474,999 | 1.53 | 0.0411 | A_19_P00321312 |
| HPN | 1.53 | 0.0223 | A_23_P406782 |
| QPCT | 1.53 | 0.0100 | A_24_P71468 |
| chr11:115,810,869-115,810,928 | 1.53 | 0.0278 | A_33_P3384265 |
| EFCAB5 | 1.53 | 0.0258 | A_33_P3507270 |
| LOC100192378 | 1.53 | 0.0306 | A_32_P114003 |
| THEMIS | -1.53 | 0.0134 | A_33_P3462422 |
| ZIC1 | 1.53 | 0.0401 | A_33_P3384362 |
| PLS1 | -1.53 | 0.0010 | A_23_P211909 |
| NEUROG2 | 1.53 | 0.0297 | A_32_P438767 |
| chr3:170,410,704-170,410,763 | 1.52 | 0.0156 | A_19_P00317736 |
| WIPF3 | 1.52 | 0.0075 | A_33_P3397840 |
| PKHD1 | 1.52 | 0.0422 | A_23_P402187 |
| FIGF | 1.52 | 0.0294 | A_23_P45185 |
| RAMP2 | 1.52 | 0.0229 | A_24_P116710 |
| chr3:5,806,312-5,806,371 | 1.52 | 0.0040 | A_19_P00810974 |
| WWC1 | -1.52 | 0.0236 | A_23_P81392 |
| EHF | 1.52 | 0.0481 | A_23_P203540 |
| PAX2 | 1.52 | 0.0304 | A_33_P3343066 |
| PRH2 | 1.52 | 0.0121 | A_33_P3422777 |
| LOC285484 | 1.52 | 0.0376 | A_33_P3239233 |
| PSME4 | -1.52 | 0.0371 | A_33_P3361472 |
| REG1P | 1.52 | 0.0460 | A_23_P108546 |
| FAT3 | 1.52 | 0.0393 | A_24_P16833 |
| XLOC_008703 | 1.52 | 0.0036 | A_19_P00316846 |
| chr13:19,761,342-19,761,401 | 1.52 | 0.0480 | A_33_P3269817 |
| - | 1.52 | 0.0390 | A_33_P3244651 |
| MPDZ | 1.52 | 0.0115 | A_23_P396328 |
| MCOLN3 | 1.52 | 0.0292 | A_23_P12241 |
| KRT77 | 1.52 | 0.0497 | A_33_P3315288 |
| TMPRSS3 | 1.52 | 0.0485 | A_23_P80162 |
| chr15:69,383,452-69,383,511 | 1.52 | 0.0468 | A_19_P00322669 |
| ERO1LB | -1.52 | 0.0143 | A_33_P3350703 |
| FABP4 | 1.52 | 0.0047 | A_23_P8820 |
| PLA2G12A | -1.52 | 0.0078 | A_24_P706752 |
| LOC100216001 | 1.52 | 0.0311 | A_33_P3339070 |
| PLA2R1 | 1.52 | 0.0331 | A_23_P142830 |
| chr1:89,387,086-89,387,145 | 1.52 | 0.0487 | A_19_P00810726 |
| SLFN12 | 1.51 | 0.0343 | A_23_P118536 |
| XLOC_009766 | -1.51 | 0.0144 | A_19_P00320804 |
| LOC90834 | -1.51 | 0.0126 | A_24_P213715 |
| APOBEC3A | 1.51 | 0.0454 | A_32_P9543 |
| chr1:89,387,086-89,387,145 | 1.51 | 0.0472 | A_33_P3363680 |
| ZNF71 | 1.51 | 0.0212 | A_23_P345674 |
| KALRN | 1.51 | 0.0358 | A_23_P307563 |
| chr1:1,002,000-1,002,059 | 1.51 | 0.0078 | A_32_P157213 |
| KIAA0825 | 1.51 | 0.0415 | A_23_P422245 |
| XLOC_004018 | 1.51 | 0.0388 | A_19_P00317287 |
| CTXN3 | 1.51 | 0.0236 | A_32_P189413 |
| chr9:14,589,556-14,589,615 | 1.51 | 0.0021 | A_19_P00319961 |
| chr9:95,650,851-95,650,910 | -1.51 | 0.0458 | A_33_P3336023 |
| FST | 1.51 | 0.0417 | A_23_P110531 |
| chr8:16,418,390-16,418,449 | 1.51 | 0.0414 | A_19_P00808546 |
| ST8SIA4 | 1.51 | 0.0433 | A_23_P435601 |
| chr10:91,454,466-91,454,525 | 1.51 | 0.0253 | A_19_P00811930 |
| chr10:80,529,296-80,529,355 | 1.51 | 0.0210 | A_19_P00330580 |
| chr8:37,270,167-37,270,226 | 1.51 | 0.0372 | A_19_P00316483 |
| chr7:7,288,141-7,288,200 | -1.51 | 0.0396 | A_19_P00805702 |
| LOC100131802 | 1.51 | 0.0405 | A_33_P3292269 |
| CABS1 | 1.51 | 0.0212 | A_24_P388662 |
| chr12:59,994,691-59,994,750 | -1.50 | 0.0432 | A_19_P00326456 |
| FIGN | 1.50 | 0.0139 | A_33_P3354731 |
| NCAM1 | 1.50 | 0.0064 | A_33_P3363804 |
| chr8:127,121,369-127,121,428 | 1.50 | 0.0367 | A_19_P00807556 |
| DNAH10 | 1.50 | 0.0294 | A_33_P3250333 |
| OR51A2 | 1.50 | 0.0481 | A_33_P3268060 |
| DKFZP547J0410 | 1.50 | 0.0422 | A_33_P3567892 |
| FAM59B | 1.50 | 0.0448 | A_23_P325924 |
| chr14:34,082,035-34,082,094 | 1.50 | 0.0421 | A_33_P3390678 |
| HTN3 | 1.50 | 0.0461 | A_23_P212945 |

## Table S9: Genes regulated by STAT3 and STAiR18 knockdown

**Table S9: Genes regulated by the knockdown of STAT3 and STAiR18.** Permanently IL-6-stimulated INA-6 cells were transfected with siRNAs targeting the STAT3 mRNA, STAiR18 exon 1 and a negative control siRNA. 40 h posttransfection RNA was isolated, DNase-digested, followed by library preparation and expression analysis by microarrays. Regulated genes were identified and mapped to the human genome using GeneSpring software. 58 differentially regulated candidates (fold change of more than 1.5 compared to the controls and a p-value of less than 0.05) are listed. Candidates shown in bold italic letters were chosen for validation by qPCR.

| Gene name | Fold change  siSTAT3 | p-value  siSTAT3 | Fold change  siSTAiR18 | p-value  siSTAiR18 | Probe |
| --- | --- | --- | --- | --- | --- |
| *PTP4A1* | ***-2.13*** | ***0.0177*** | ***-3.19*** | ***0.0273*** | ***A_19_P00315634*** |
| chr9:476,688-476,747 | 1.66 | 0.0462 | 1.65 | 0.0119 | A_19_P00317621 |
| chr3:171,506,466-171,506,525 | 2.62 | 0.0233 | 1.62 | 0.0354 | A_19_P00319324 |
| chr8:16,113,842-16,113,901 | 1.93 | 0.0307 | 1.80 | 0.0173 | A_19_P00319993 |
| chr4:84,771,772-84,771,831 | 1.54 | 0.0007 | 2.05 | 0.0032 | A_19_P00322146 |
| chr15:69,383,452-69,383,511 | 1.52 | 0.0436 | 1.52 | 0.0468 | A_19_P00322669 |
| chr13:43,406,188-43,406,247 | 3.11 | 0.0402 | 2.97 | 0.0130 | A_19_P00325780 |
| chr1:116,627,442-116,627,501 | 1.86 | 0.0349 | 2.21 | 0.0301 | A_19_P00809224 |
| chr19:12,114,654-12,114,713 | 1.63 | 0.0245 | 1.84 | 0.0409 | A_19_P00811229 |
| chr12:122,031,364-122,031,423 | 2.06 | 0.0139 | 1.93 | 0.0453 | A_19_P00812235 |
| AGTR1 | 2.34 | 0.0323 | 1.92 | 0.0247 | A_23_P166616 |
| TRIM29 | 2.02 | 0.0010 | 1.59 | 0.0288 | A_23_P203267 |
| PLS1 | -1.66 | 0.0184 | -1.53 | 0.0010 | A_23_P211909 |
| CHL1 | 1.97 | 0.0339 | 2.15 | 0.0251 | A_23_P212241 |
| AHR | -1.86 | 0.0070 | -1.64 | 0.0432 | A_23_P215566 |
| CCR3 | 1.72 | 0.0149 | 1.56 | 0.0034 | A_23_P250302 |
| DSC1 | 2.06 | 0.0314 | 1.58 | 0.0391 | A_23_P38696 |
| RAB37 | 2.43 | 0.0257 | 1.71 | 0.0007 | A_23_P414654 |
| HTR5A | 1.87 | 0.0295 | 1.71 | 0.0278 | A_23_P42565 |
| ST8SIA4 | 1.52 | 0.0468 | 1.51 | 0.0433 | A_23_P435601 |
| OSTBETA | 1.55 | 0.0452 | 1.63 | 0.0344 | A_23_P436284 |
| ZNF236 | -1.98 | 0.0112 | -1.71 | 0.0110 | A_23_P55601 |
| SVEP1 | 1.65 | 0.0498 | 1.53 | 0.0359 | A_24_P187774 |
| FLJ37201 | 2.05 | 0.0471 | 2.40 | 0.0138 | A_24_P246091 |
| C17orf87 | 1.62 | 0.0301 | 2.13 | 0.0117 | A_24_P272451 |
| TBX5 | 1.52 | 0.0149 | 1.94 | 0.0092 | A_24_P30557 |
| CNTNAP3 | 1.81 | 0.0294 | 2.11 | 0.0460 | A_24_P418203 |
| KLHL24 | 1.56 | 0.0497 | 1.74 | 0.0463 | A_24_P521994 |
| CERS3 | 1.69 | 0.0376 | 1.76 | 0.0383 | A_24_P943017 |
| ANKRD30B | 1.99 | 0.0085 | 1.56 | 0.0286 | A_33_P3277659 |
| OR8B2 | 1.93 | 0.0095 | 1.67 | 0.0248 | A_33_P3253578 |
| KRTAP9-8 | 2.67 | 0.0230 | 1.57 | 0.0132 | A_33_P3307457 |
| PRAMEF4 | 2.62 | 0.0290 | 2.63 | 0.0144 | A_33_P3319896 |
| EFCAB5 | 2.93 | 0.0319 | 1.92 | 0.0463 | A_33_P3341399 |
| SIRPG | 2.11 | 0.0086 | 1.66 | 0.0089 | A_33_P3343873 |
| chr8:108,569,479-108,569,537 | -1.59 | 0.0411 | -1.76 | 0.0494 | A_33_P3345821 |
| RBMY3AP | 1.70 | 0.0462 | 1.69 | 0.0265 | A_33_P3393121 |
| chr15:31,745,355-31,745,414 | 2.02 | 0.0065 | 1.67 | 0.0414 | A_33_P3357232 |
| TNKS1BP1 | -1.67 | 0.0464 | -1.60 | 0.0361 | A_33_P3365732 |
| OR6C4 | 2.25 | 0.0494 | 1.80 | 0.0429 | A_33_P3379190 |
| VAX1 | 2.60 | 0.0414 | 1.56 | 0.0288 | A_33_P3381398 |
| chr22:21,583,027-21,583,086 | 1.68 | 0.0122 | 2.75 | 0.0056 | A_33_P3383075 |
| LCE5A | 2.17 | 0.0090 | 2.35 | 0.0321 | A_33_P3403018 |
| ZSCAN5B | 1.50 | 0.0366 | 1.59 | 0.0323 | A_33_P3406651 |
| LOC100129894 | 2.82 | 0.0094 | 1.83 | 0.0004 | A_33_P3416398 |
| chr20:30,009,242-30,009,301 | 1.76 | 0.0441 | 1.85 | 0.0344 | A_33_P3417602 |
| LOC286059 | 1.93 | 0.0341 | 1.65 | 0.0370 | A_33_P3630785 |
| chr1:111,030,303-111,030,362 | 1.83 | 0.0112 | 1.62 | 0.0403 | A_33_P3787645 |
| ARHGEF10L | 2.17 | 0.0117 | 1.64 | 0.0061 | A_33_P3799936 |
| LMF1 | 2.25 | 0.0333 | 1.74 | 0.0166 | A_33_P3883985 |
| GUCY2C | 1.87 | 0.0094 | 1.55 | 0.0332 | A_23_P76312 |
| SLC17A8 | 1.90 | 0.0114 | 1.68 | 0.0173 | A_24_P124647 |
| HTR3D | 2.69 | 0.0384 | 3.10 | 0.0317 | A_24_P14974 |
| LOC100133857 | 1.73 | 0.0378 | 1.55 | 0.0044 | A_33_P3280360 |
| CNTLN | 1.52 | 0.0280 | 1.71 | 0.0134 | A_33_P3296240 |
| PRSS55 | 1.87 | 0.0490 | 1.90 | 0.0197 | A_33_P3336944 |
| chr2:67,402,661-67,402,720 | 2.19 | 0.0318 | 3.06 | 0.0028 | A_33_P3299791 |
| HMGA2 | 1.82 | 0.0204 | 1.63 | 0.0129 | A_23_P95930 |

## Table S10: RNA interaction partners of STAiR18

**Table S10: RNA interaction partners of STAiR18.** STAiR18 RNA interaction partners were identified by ChIRP RNA-sequencing performed with permanently IL-6-treated INA-6 cells. A ChIRP experiment was performed using oligonucleotides targeting STAiR18 RNA at exons 1 and 2, and oligonucleotides targeting bacterial lacZ RNA as a negative control. RNA isolated from the pulldown fractions was DNase-digested, used for library preparation, and subjected to next-generation sequencing. Reads were mapped to the human genome (Hg19) by TopHat (21) software and candidates with a differential expression of more than 10-fold as compared to lacZ were listed.

| Gene name | Fold enrichment | Position | Intragenic position | Repeat masker |
| --- | --- | --- | --- | --- |
| LINC00152 | 12.54 | chr2:87769776-87769800 | Intron | - |
| LMNB1 | 12.33 | chr5:126158551-126158575 | Exon | - |
| UBB | 12.21 | chr17:16285226-16285250 | Exon | - |
| RALGPS2 | 12.14 | chr1:178806351-178806375 | Intron | LINE, SINE |
| NEB | 11.93 | chr2:152463126-152463150 | Exon/ intron | - |
| LOC541471 | 11.88 | chr2:112237501-112237525 | Intron | - |
| SYN3 | 11.8 | chr22:33094376-33094400 | Intron | - |
| AK3 | 11.48 | chr9:4734076-4734100 | Intron | LTR |
| POLA1 | 11.4 | chrX:24761351-24761375 | Exon | - |
| abParts | 11.19 | chr14:106204126-106204150 | Intron | - |
| RC3H1 | 11.08 | chr1:173948151-173948175 | Intron | DNA |
| USP34 | 11.08 | chr2:61450926-61450950 | Intron | SINE |
| RMND5A | 11.02 | chr2:87821776-87821800 | Intron | - |
| FASTKD2 | 11.01 | chr2:207656501-207656525 | Exon | - |
| ARSG | 10.96 | chr17:66399451-66399475 | Intron | - |
| MIR548G, CMSS1, FILIP1L | 10.87 | chr3:99660376-99660400 | Intron | - |
| HNRNPUL2-BSCL2 | 10.76 | chr11:62491426-62491450 | Exon | - |
| ERCC6 | 10.75 | chr10:50708601-50708625 | Exon | - |
| loc256021 | 10.62 | chr12:92514851-92514875 | Intron | LINE |
| ABCC10 | 10.57 | chr6:43411151-43411175 | Exon/intron | - |
| ERC1 | 10.44 | chr12:1525451-1525475 | Intron | - |
| MAGEA4 | 10.43 | chrX:151091926-151091950 | Exon | - |
| PPP3CA | 10.38 | chr4:102151801-102151825 | Intron | - |
| ASB6 | 10.29 | chr9:132400001-132400025 | Exon | - |
| KHSRP | 10.28 | chr19:6419226-6419250 | Exon | - |
| loc10088142, NBPF10, RNF115 | 10.27 | chr1:145688251-145688275 | Intron1+2, exon3 | - |
| ANP32A | 10.18 | chr15:69094876-69094900 | Intron | LINE |
| FOXN3 | 10.15 | chr14:90046001-90046025 | Intron | LINE |
| KIF18A | 10.15 | chr11:28074076-28074100 | Intron | LINE |
| TPCN1 | 10.14 | chr12:113664726-113664750 | Exon | - |
| UBR3 | 10.12 | chr2:170884501-170884525 | Intron | SINE |
| RASA4, POLR2J2 | 10.10 | chr7:102309351-102309375 | Intron1, intron/exon2 | - |
| RMND5A , linc00152 | 10.08 | chr2:87769676-87769700 | Intron | - |
| STAT3 | **10.07** | **chr17:40500701-40500725** | **Intron** | **SINE** |
| PTP4A1 | 6.42 | chr6:64290051-64290075 | Exon | - |
